# Honeybee colonies compensate for pesticide-induced effects on royal jelly composition and brood survival with increased brood production

**DOI:** 10.1038/s41598-020-79660-w

**Published:** 2021-01-08

**Authors:** Matthias Schott, Maximilian Sandmann, James E. Cresswell, Matthias A. Becher, Gerrit Eichner, Dominique Tobias Brandt, Rayko Halitschke, Stephanie Krueger, Gertrud Morlock, Rolf-Alexander Düring, Andreas Vilcinskas, Marina Doris Meixner, Ralph Büchler, Annely Brandt

**Affiliations:** 1 Institute of Insect Biotechnology, Justus-Liebig-University Giessen, Giessen, Germany; 2 Animal Ecology I, University of Bayreuth, Universitaetsstr. 30, 95447 Bayreuth, Germany; 3 LLH Bee Institute, Erlenstr. 9, 35274 Kirchhain, Germany; 4 Biosciences, University of Exeter, Exeter, EX4 4PS UK; 5 Environment and Sustainability Institute, Biosciences, University of Exeter, Penryn Campus, Penryn, TR10 9FE Cornwall UK; 6 Mathematical Institute, Justus-Liebig University of Giessen, Giessen, Germany; 7 Institute of Pharmacology, Biochemical-Pharmacological Centre (BPC), University of Marburg, Karl-von-Frisch-Str. 1, 35032 Marburg, Germany; 8 Department of Molecular Ecology, Max Planck Institute for Chemical Ecology, Hans Knöll Str. 8, 07745 Jena, Germany; 9 Chair of Food Science, Institute of Nutritional Science, Justus Liebig University Giessen, Heinrich-Buff-Ring 26-32, 35392 Giessen, Germany; 10 Institute of Soil Science and Soil Conservation, Justus-Liebig-University of Giessen, Heinrich-Buff-Ring 58, 35392 Giessen, Germany

**Keywords:** Environmental impact, Physiology, Agroecology, Behavioural ecology, Ecophysiology, Molecular ecology

## Abstract

Sublethal doses of pesticides affect individual honeybees, but colony-level effects are less well understood and it is unclear how the two levels integrate. We studied the effect of the neonicotinoid pesticide clothianidin at field realistic concentrations on small colonies. We found that exposure to clothianidin affected worker jelly production of individual workers and created a strong dose-dependent increase in mortality of individual larvae, but strikingly the population size of capped brood remained stable. Thus, hives exhibited short-term resilience. Using a demographic matrix model, we found that the basis of resilience in dosed colonies was a substantive increase in brood initiation rate to compensate for increased brood mortality. However, computer simulation of full size colonies revealed that the increase in brood initiation led to severe reductions in colony reproduction (swarming) and long-term survival. This experiment reveals social regulatory mechanisms on colony-level that enable honeybees to partly compensate for effects on individual level.

## Introduction

Bees provide vital pollination services to crops and wild plants and thus play a key role in global food security and the maintenance of biodiversity^1,2^. However, the number of managed honeybees and wild bees has declined over the last few decades in North America^3–5^ and Europe^5,6^. The substantial environmental impact on honeybee colonies is thought to be driven mainly by pathogens and parasites^7^, but the quality and diversity of the pollen diet^8–10^ as well as exposure to pesticides^5,11^ may also affect bee health and survival. In particular, the widespread use of neonicotinoids is considered a major threat to bee health^5,12–17^. Neonicotinoid insecticides are among the most important crop protection chemicals and they are widely used in seed dressings and spray applications^13,14,18^. Neonicotinoids are neurotoxins that act as nicotinic acetylcholine receptor agonists to disrupt neuronal cholinergic signal transduction, leading to abnormal behavior in target pests, or immobility and death at higher doses^13,19,20^. Non-target insects such as bees also come into contact with these systemic insecticides via pollen, nectar or guttation droplets. Insecticide residues are carried back to the colony by forager bees and remain stored in beebread or honey until they are fed to larvae, workers, drones, or the queen^7,21,22^.

Honeybees are highly eusocial insects, with labor division and intensive brood care, where adult members of a colony care cooperatively for the young^23^. As nurse bees, young workers produce brood food (worker jelly) in the hypopharyngeal gland (HPG). The development of this gland begins soon after hatching, and the lobes of the gland (acini) reach their maximum size and weight when the workers are 8–12 days old^24,25^. Several chemical compounds reduce the size of the HPG in nurse bees: neonicotinoid insecticides, the varroazide coumaphos, the insect growth regulator fenoxycarb, and the fungicide captan^26–29^. The effect of neonicotinoids on brood mortality is a matter of discussion. Laboratory survival tests show that artificially-reared larvae can consume neonicotinoids in quantities larger than would ever be encountered in the field without direct lethal effects^30^. However, a reduced number of brood cell has been observed in some field studies^11^, which may indicate that neonicotinoids affect brood mortality indirectly, for example due to insufficient brood care by nurse bees affected by the pesticides. Recently, Siefert et al.^31^ were able to demonstrate by automated long-term behavioral observations that exposure to neonicotinoids indeed reduces the number and duration of feeding visits of worker larvae. Numerous studies have demonstrated that neonicotinoids affect the behavioral or physiological traits of individual honeybees^21,32–35^. However, the assessment of the impact on colony growth and survival has been divergent, ranging from negative impact^36–39^ to no impact^39–43^, sometimes even in the same study^38^. We therefore investigated this phenomenon using small nucleus colonies rather than full-sized standard colonies, thus reducing the number of individuals substantially. This allowed us to study the sublethal effects of the neonicotinoid clothianidin at the physiological level in individual larvae and nurse bees and also to determine its impact on brood development of an entire colony. Using a demographic modeling approach, we tested for compensatory mechanisms at the colony level and extrapolated the results from small nucleus colonies to full-sized colonies using the agent-based honeybee model BEEHAVE^44^.

## Results

The experimental scheme, climate recordings, clothianidin levels in the spiked feeding solutions, clothianidin residues in worker bees, protein content and antimicrobial activity of worker jelly and the statistical report are presented in the supplementary data (SFigs 1–4).

### Hypopharyngeal gland size

**Figure 1:**
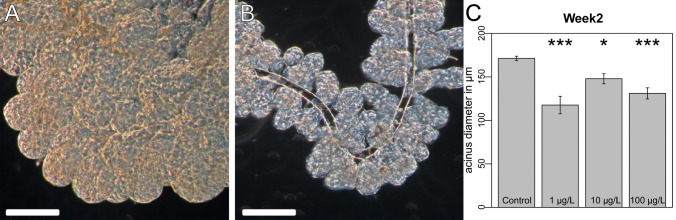
Hypopharyngeal gland size in age-defined worker bees. Age-defined worker bees, which were placed in the colonies and recovered in week 2 (12 days after hatching), were analyzed for the acini size of the HPG. (**A**) HPG of a control worker bee. (**B**) HPG of a worker bee from a colony exposed to 100 µg/L clothianidin (bar = 100 µm). (**C**) Clothianidin exposure reduced the acinus diameter of HPGs. Control individuals had larger acini than workers exposed to clothianidin (control, n = 31; 1 µg/L, n = 22; 10 µg/L, n = 22; 100 µg/L, n = 23). Mixed-effects ANOVA, (*P* = 0.0120; post hoc Dunnett contrasts, control vs. 1 µg/L, *P* < 0.001; control vs. 10 µg/L, *P* = 0.0345; control vesus 100 µg/L, *P* = 0.0027). Raw data and technical details of the analysis are presented in the supplementary statistical report, “Hypopharyngeal gland size” section.

After spending 12 days in the experimental colonies, age-defined workers of the control hives had significantly larger acini than those of colonies exposed to clothianidin (Fig. 1A–C; Mixed-effects ANOVA *P* = 0.01199, post hoc Dunnett contrasts: control vs. 1 µg/L: *P* < 0.001; control vs. 10 µg/L: *P* = 0.03447; control vs. 100 µg/L *P* = 0.00269). The mean diameters of the acini were 171.22 µm (SEM = 2.24, n = 30) for the controls, 129.66 µm (SEM = 8.60, n = 16) in the 1 µg/L group, 147.92 µm (SEM = 5.79, n = 22) in the 10 µg/L group, and 131.06 µm (SEM = 6.39, n = 23) in the 100 µg/L group.

In addition, we measured the acinus diameter of randomly chosen worker bees of undefined age during week 7. The mean diameters of the acini were 144.36 µm (SEM = 4.43, n = 28) for the controls, 133.59 µm (SEM = 7.61, n = 22) in the 1 µg/L group, 138.04 µm (SEM = 5.02, n = 22) in the 10 µg/L group, and 109.39 µm (SEM = 4.83, n = 29) in the 100 µg/L group. Workers in the control hives had significantly larger acini than those exposed to 100 µg/l clothianidin (mixed-effects ANOVA *P* = 0.01379; post hoc Dunnett contrasts: control vs. 100 µg/L: *P* = 0.000699). By utilization of Hive.ID as random effect in the mixed effect model (see statistics in attachment), we acknowledge that hives can have an influence on gland size. This random effect accounted for a possible hive effect on gland size. In this way, each hive got its own baseline so that we were able to analyze the effect of treatment without the effect of the colony diversity.

### HPTLC analysis of worker jelly and larvae

**Figure 2:**
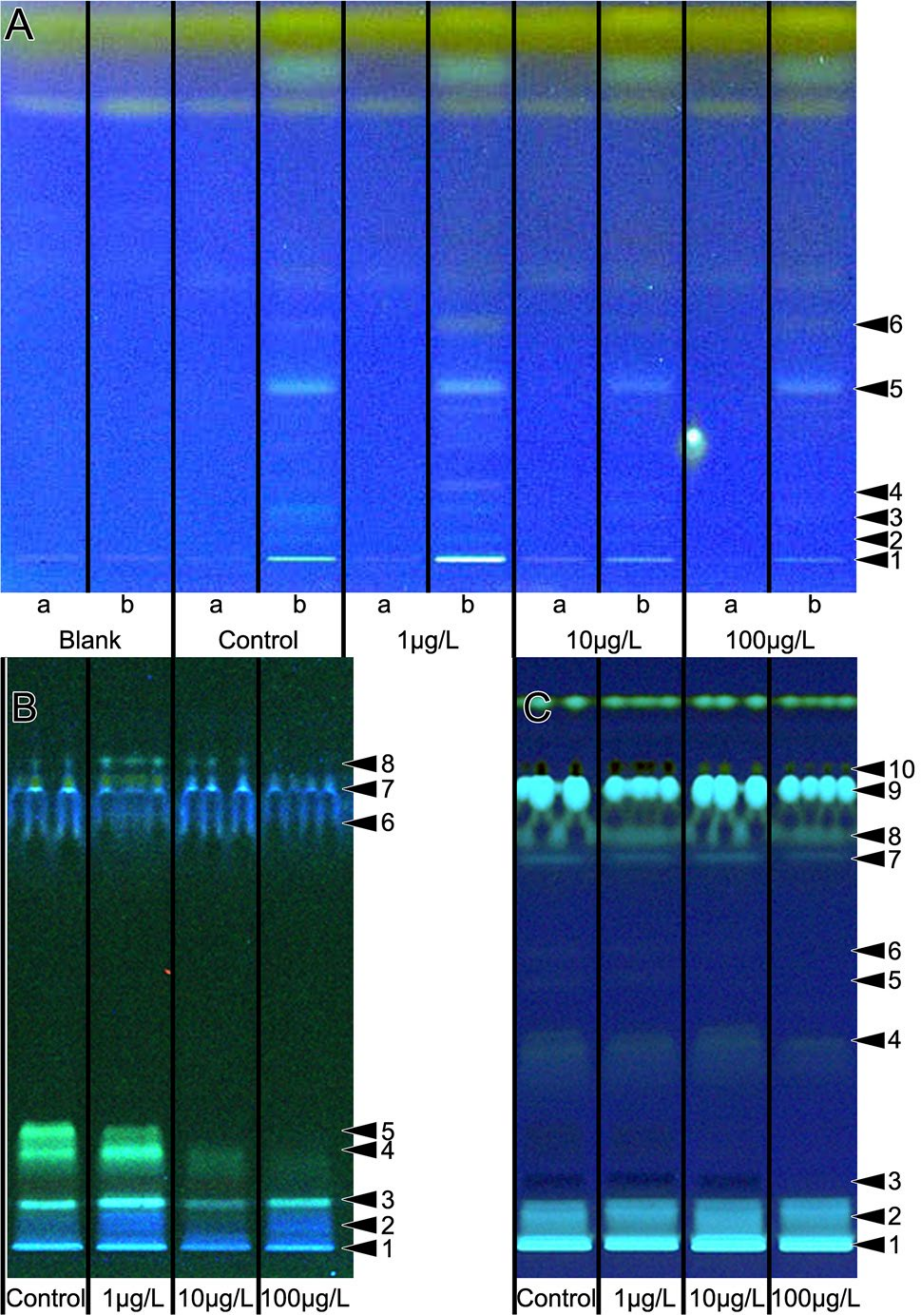
Lipid profiles of worker jelly samples (**A**) and larvae (**B**, **C**) from week 3. Samples of worker jelly (**A**) were taken using absorptive filter strips (Sugi strips), extracted with *n*-hexane and separated by HPTLC. Each Sugi strip was halved: one half without the sample (track a) and the other with absorbed sample (track b). A fresh Sugi strip from the same batch as the strips used in the experiment cut in two equal halves served as the blank. The lanes are labeled to show the dose of clothianidin treatments (control = no clothianidin). The analysis of larvae is shown before (**B**) and after derivatisation (**C**). For worker jelly, the declining quantity of substances was observed for the lipid profiling (UV 366 nm after derivatization). The latter also was evident for the larvae. Numbers on the right side indicate differences, see SFig 3B, F and H.

The lipid composition of worker jelly during weeks 3 and 7 was analyzed using non-targeted High-performance thin-layer chromatography (HPTLC). Semi-quantitative differences in the lipid composition were observed in the 10 and 100 µg/L clothianidin treatment groups compared to the control colonies in week 3. Our robust non target screening HPTLC analysis indicated a reduction of lipids (Fig. 2A) and in antimicrobial activity against the luminescent *A. fischeri* bacteria (SFig 3)^45^. The lipid profiles of larvae also indicated differences between the treatment groups during week 3 (Fig. 2B,C). The blank Sugi strips as well as the part of the Sugi test strip that had not contacted the sample showed no lipid signals. The results of the combined, orthogonal physicochemical analysis and activity-based detection of HPTLC-separated compounds suggest a decline in antimicrobial activity corresponding to the decline in the amount of lipids in the worker jelly. The lipid profiles of larvae also showed semi-quantitative differences between the different treatment groups during week 3 (Fig. 2B,C). The profiling of larval liposoluble components showed a similar decline in the concentration of several compounds in colonies treated with 10 and 100 µg/L clothianidin compared to untreated control larvae. The total amount of protein in the worker jelly did not differ between the treatment groups (SFig. 4).

### Brood survival

**Figure 3:**
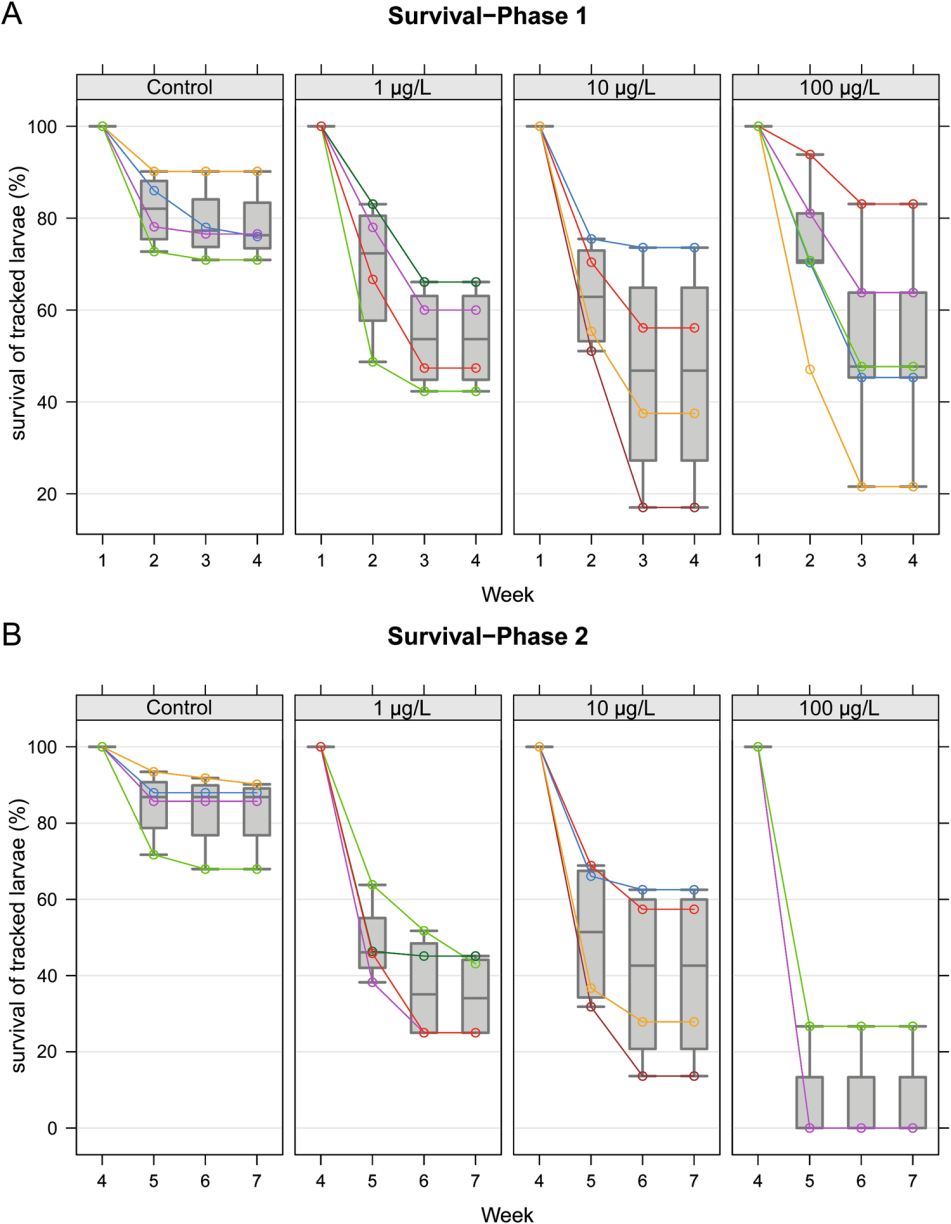
Survival of individually tracked larvae. Individually marked brood cells were tracked from the first larval stage (day 4–5 after egg laying) to emergence. (**A**) The percentage of brood surviving from week 1 to week 4 (survival phase A). Colonies exposed to clothianidin showed a reduced brood survival. Clothianidin exposure significantly affects brood survival (exposure-by-time interaction in mixed-effects ANOVA, *P* = 0.0293). One-sided tests for post hoc Dunnett contrasts yielded no significant *P* values in week 2, but in week 3 all clothianidin treatments showed significant differences (control vs. 1 µg/L, *P* = 0.0414; control vs. 10 µg/L, *P* = 0.0241; control vs. 100 µg/L, *P* = 0.0418). (**B**) The percentage of brood surviving from week 4 to week 7 (survival phase B). Clothianidin exposure reduced the number of surviving brood (exposure-by-time interaction in mixed effects ANOVA, *P* = 0.03776). One-sided tests for post hoc Dunnett contrasts in weeks 5 and 6: each clothianidin treatment has a significantly lower brood survival than the control group (*P* < 0.0001 in all cases). Technical details of the analysis are provided in the supplementary statistical report, “Brood survival” section.

To assess whether clothianidin exposure affected brood survival, we traced the development of individual, newly-hatched larvae. At the beginning of the experiment (brood survival phase A, weeks 1–4) as shown in Fig. 3A, an average of 78% of the tracked larvae in control colonies developed normally to capped brood and the cells were empty within the expected time frame (SD 8.2%, for a total of 230 larvae in week 1 in n = 4 hives), which was considered as survival of the larvae. In contrast, an average of only 54% of the larvae in the 1 µg/L treatment group survived (SD 11%, for a total of 244 larvae in week 1 in n = 4 hives). In colonies exposed to 10 µg/L clothianidin, only 46% of the larvae survived (SD 24%, for a total of 248 larvae in week 1 in n = 4 hives) of the monitored larvae survived. In the 100 µg/L treatment group, only 52% of the larvae survived (SD 23%, for a total of 303 larvae in week 1 in n = 5 hives). Clothianidin exposure significantly affected the brood survival time (exposure-by-time interaction in mixed-effects ANOVA, *P* = 0.02929; one-sided tests for post hoc Dunnett contrasts in weeks 2 and 3). In week 2, none of the clothianidin treatments reduced brood survival significantly, but in week 3 each clothianidin treatment had a significant effect (control vs. 1 µg/L, *P* = 0.0414; control vs. 10 µg/L, *P* = 0.0241; control vs. 100 µg/L, *P* = 0.0418).

In brood survival phase B (weeks 4–7) as shown in Fig. 3B, the average brood survival was 82.9% (SD = 10.2%, for a total of 260 larvae in week 4 in n = 4 hives) in the control group, 34.6% (SD = 11.1%, for a total of 280 larvae in week 4 in n = 4 hives) in the 1 µg/L treatment group, and 40.3% (SD = 23.5%, for a total of 218 larvae in week 4 in n = 4 hives) in the 10 µg/L treatment group. In all colonies exposed to 100 µg/L clothianidin, fewer than 50 larvae were present by week 4 (11–34 larvae per colony, with an average number of larvae of 19.25). The average brood survival was 6.7% (SD = 13.3%, for a total of 77 larvae in week 4 in n = 4 hives) in the 100 µg/L group. Clothianidin exposure significantly affected brood survival over time (exposure-by-time interaction in mixed-effects ANOVA, *P* = 0.03776; one-sided tests for post hoc Dunnett contrasts in weeks 5 and 6 in each clothianidin treatment group revealed a significantly lower brood survival compared to the control group *P* < 0.0001 in all tests). For the detailed analysis, see the supplementary statistical report.

### Brood quantification

**Figure 4:**
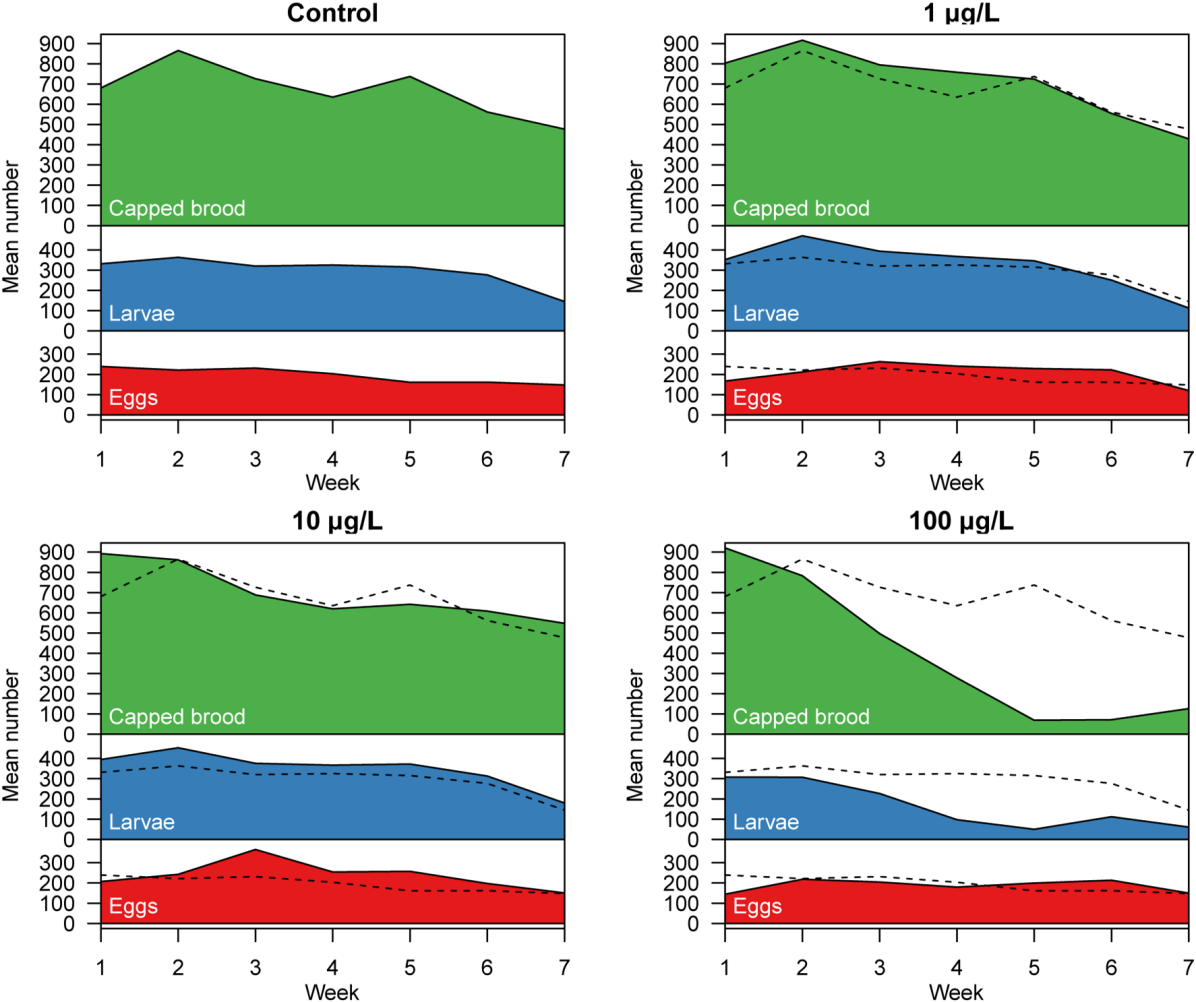
Brood quantification. Every week, the mean number of eggs (red), larvae (blue), and pupae (green) was determined for every colony over a period of 7 weeks. The black dashed line depicts the mean values of the control. In colonies exposed to lower or medium concentrations of clothianidin, the mean number of eggs, larvae or capped brood were similar or tended to exceed the numbers of the control group. The trends for the eggs, larvae, and capped brood cells all differed significantly between the treatment groups (main effects of quadratic time and their interaction with treatment in linear mixed-effects models, *P* < 0.001 in all cases). Pairwise comparisons showed that the temporal profiles of eggs differed significantly from the control for all clothianidin doses (*P* < 0.03 in all cases), and that for larvae and capped brood cells only the 100 µg/L group differed significantly from the control. Weeks 6 and 7 were already in September, thus a seasonally related drop in brood nest size was observed at the end of the experiment. Raw data and technical details of the analysis are provided in the supplementary statistical report, “HPTLC analysis of worker jelly and larvae” section.

To achieve the total quantification of brood cells of a colony, all cells containing eggs, larvae, and pupae were counted at all sampling time points for each colony (Fig. 4). Detailed results and analysis are provided in the supplementary statistical report. The temporal profiles for the mean numbers of eggs differed significantly between the treatment groups (main effect of quadratic time and its interaction with treatment in a linear mixed-effects model, *P* <  < 0.0001). When comparing the parabolic temporal profiles of eggs in the treatment vs. control groups we observed significant differences in both the (local) slope during week 3 and the (global) curvature of the temporal trend in mean egg numbers (*P* < 0.03 in all tests except 100 µg/L group vs. control, which showed no difference in curvature).

Likewise, the temporal profiles of the mean number of larvae differed significantly between the treatment groups (main effect of quadratic time and its interaction with treatment in a linear fixed-effects model, given that the random hive effect was non-significant, *P* < 0.0001). However, when comparing the parabolic temporal profiles of larvae in the treatment groups with the control, we found that only the profile of the 100 µg/L treatment group differed significantly from the control in terms of the (local) slope during week 3 and the (global) curvature (*P* < 0.014 in both cases).

The temporal profiles of the mean numbers of capped brood cells also differed significantly between the treatment groups (main effect of quadratic time and its interaction with treatment in a linear fixed-effects model, given that the random hive effect was non-significant, *P* = 0.00035). And as for the larvae, comparing the parabolic profiles of capped brood cells in the treatment groups with the control, we found that only the profile of the 100 µg/L group differed significantly from the control in terms of the (local) slope during week 3 and the (global) curvature (*P* < 0.01 in both cases).

### Demographic compensation

**Figure 5:**
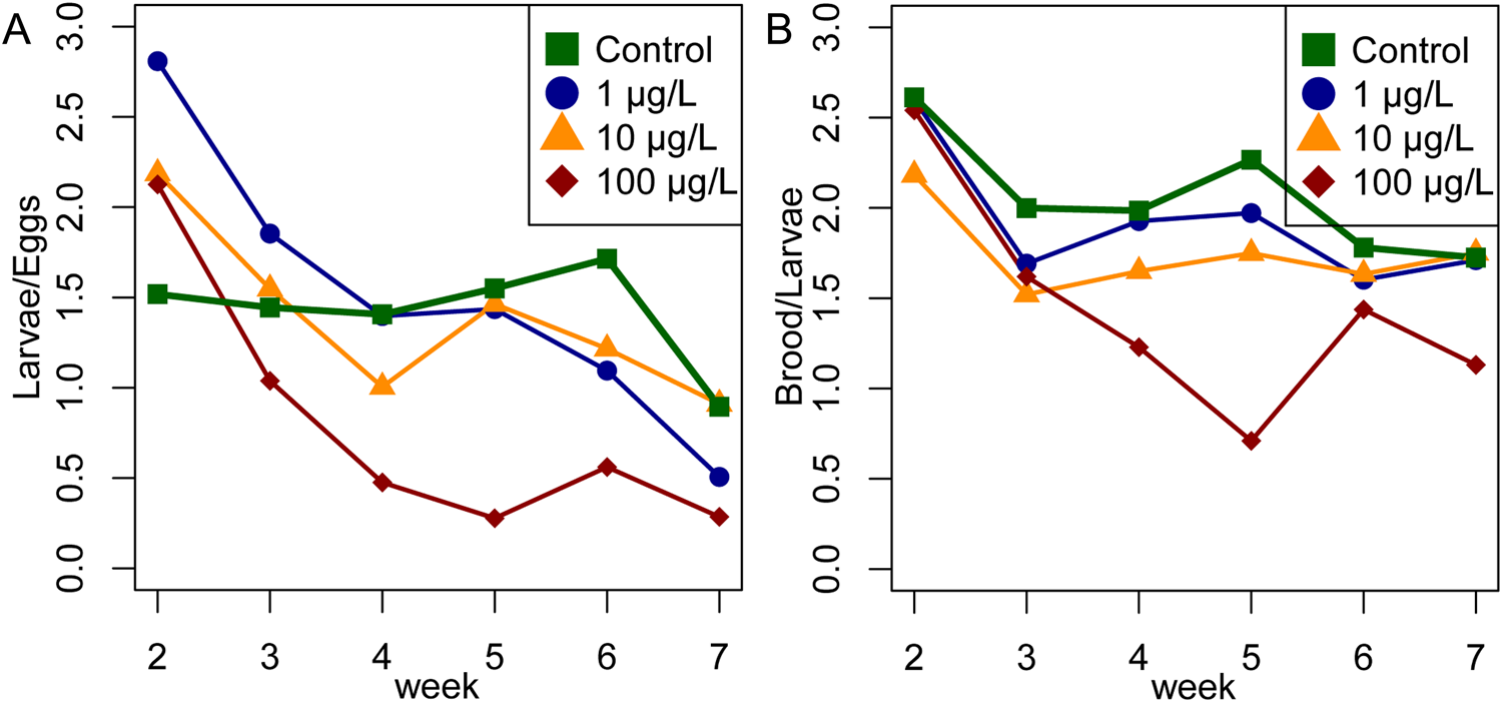
Ratio between developmental stages. (**A**) The ratio of the number of larvae/eggs. The eggs in week 2 become the larvae of week 3, so the number of larvae is shifted by 1 week. The ratio of controls is stable throughout the experiment (mean = 1.5) until week 7, where a seasonally related drop in brood activity was observed. (**B**) The ratio of capped brood/larvae (number of capped brood shifted by 1 week) was stable in the controls (mean = 2.06), and the 1 µg/L (mean = 1.92) and 10 µg/L (mean = 1.7) groups, but dropped in the 100 µ/L treatment group in weeks 4 and 6 (mean = 1.44).

The demographic model was used to estimate the number of new larvae that must hatch each week in order to maintain a stable number of larvae given the survivorship schedule of larvae week by week. In order to maintain a stable larval population, the colonies dosed with 1 µg/L clothianidin needed to hatch 1.57-fold more larvae per week than untreated controls, and this increased to 1.75-fold and 1.91-fold in the colonies dosed with 10 and 100 µg/L clothianidin, respectively (Table 1). In the control colonies, a relatively stable ratio between the brood stages was maintained throughout the experiment. The median ratio of the number of larvae to the number of eggs was 1.57, but this declined to 1.48, 1.38 and 0.60 in the colonies dosed with 1, 10 and 100 µg/L clothianidin, respectively (Fig. 5A). The colonies exposed to clothianidin had to produce more eggs to maintain a stable number of larvae. The larvae-to-eggs ratio temporal profile differed significantly between the treatment groups (treatment-by-time interaction in a linear mixed-effects model, *P* = 0.0076). Pairwise comparisons of the treatment groups with the control revealed significant differences in the temporal profiles for the 1 and 100 µg/L treatment groups (*P* < 0.016 in both cases). The analysis was carried out by omitting an extreme outlier in week 2 in one hive of the 10 µg/L group.

**Table 1:** Modeling of demographic compensation.

Clothianidin	*L*_*t*_ observed	*h*
Control	300	56
1 µg/L	300	88
10 µg/L	300	98
100 µg/L	300	107

In order to maintain a stable larval population (L_t_ observed), colonies exposed to clothianidin would need to hatch more larvae per week than controls (*h* denotes the number of eggs that hatch into larvae each week).

The average ratio of the number of capped brood cells to the number of larvae (from the previous week, for weeks 2–7) was 2.03 (median) but was consistently slightly lower in the colonies exposed to clothianidin at doses of 1 µg/L (median = 1.92) or 10 µg/L (mean = 1.71) throughout the experiment (Fig. 5B). In the 100 µg/L treatment group, the median ratio was 1.28, but the profile was biphasic, with a median ratio of 1.58 up to the end of week 4, falling sharply to 0.17 in week 5. Nevertheless, there was no significant difference in the temporal profiles of the capped brood-to-larvae ratio between the treatment groups. Detailed results and analysis are provided in the supplementary statistical report.

### BEEHAVE simulations

To estimate the impact of our findings on colony development in standard-sized hives we applied two BEEHAVE simulations. To mimic the situation of the experimental nucleus colonies in BEEHAVE (simulation A), we tested a wide range of values for *ProteinNursesModifier_Exposed*, which represents the protein content of jelly fed to the larvae. The best fits are shown in Table 2 based on a comparison of modeled and experimental brood survival. The output of these simulations was the average brood survival when comparing brood cohort sizes at ages of 19 versus 3 days. The modeled brood survival was then compared to the results of the experimental brood survival to determine the values for *ProteinNursesModifier_Exposed* that represent the tested concentrations of clothianidin.

**Table 2:** Simulation of mating nucleus colonies in BEEHAVE.

Clothianidin	Brood survival experiments	Brood survival model	*ProteinNurses Modifier_Exposed*
control	0.8	0.92 ± 0.14	1
1 µg/L	0.4	0.38 ± 0.16	0.82
10 µg/L	0.2	0.20 ± 0.12	0.77
100 µg/L	0.0	0.02 ± 0.03	0

Modification of the factor *ProteinNursesModifier_Exposed* in BEEHAVE simulations resulted in brood survival rates similar to experimental data obtained in field experiments. The factor *ProteinNursesModifier_Exposed* reduces the protein content of the jelly fed to the larvae (*ProteinFactorNurses*) in the model. A wide range of values for *ProteinNursesModifier_Exposed* was tested, and the best fits are shown based on a comparison of modeled and experimental brood survival.

**Figure 6:**
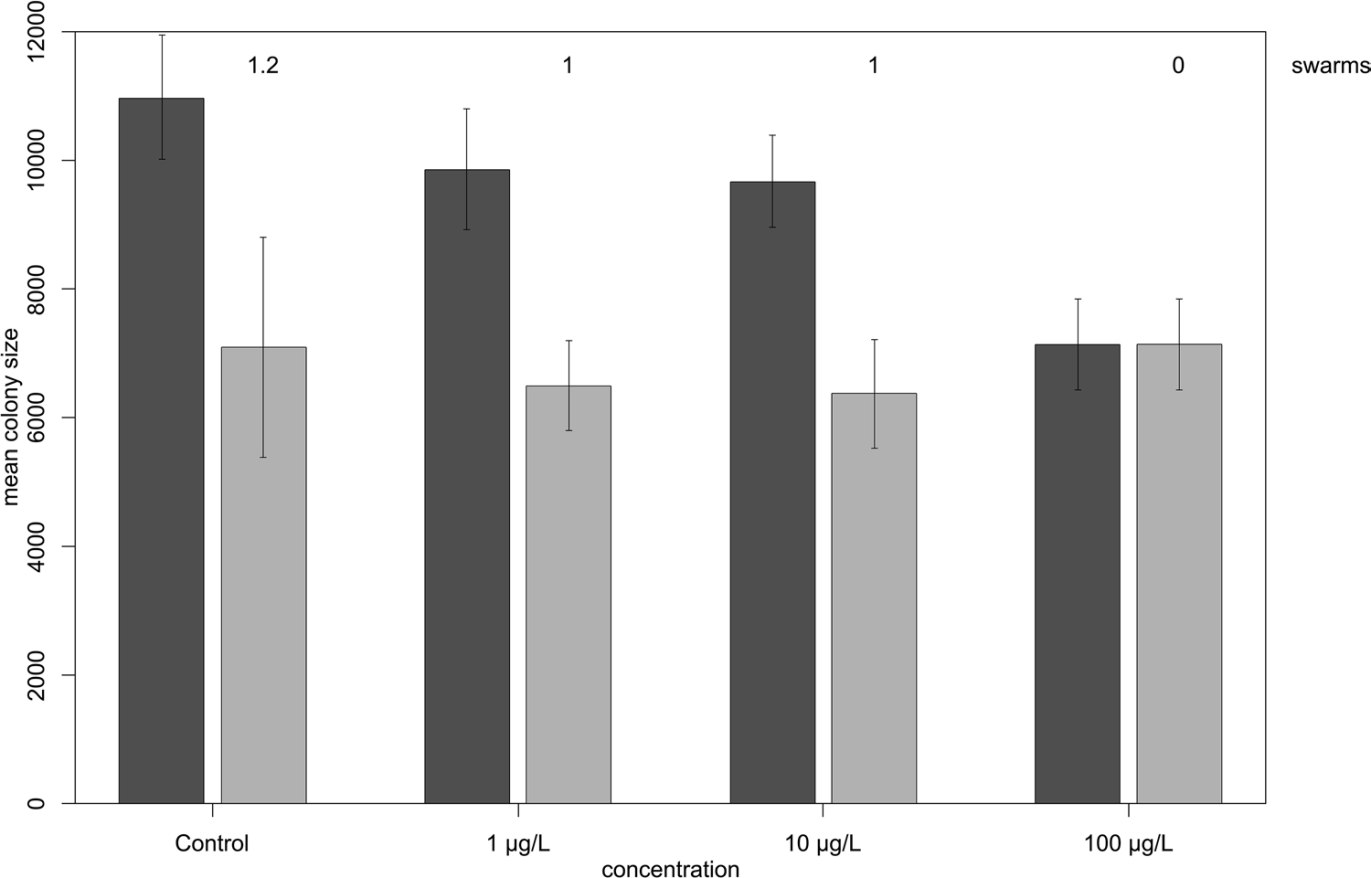
Colony sizes decline with increasing concentrations of clothianidin in simulated full-sized colonies. To estimate the potential impact of clothianidin exposure on standard-sized colonies, a BEEHAVE simulation was conducted under default settings but with less protein in the jelly (ProteinFactorNurses) as defined by ProteinNursesModifier_Exposed, with values set according to the results of the previous set of simulations (Table 2^46^). Colony sizes (mean number of worker bees) at the end of the year decreased with increasing concentrations of clothianidin, particularly when swarming was prevented (dark grey). When swarming was allowed (light grey), the control group produced 1.2 swarms in the first year. For the 1 and 10 µg/L treatment groups, each colony released a single swarm during the first year. No swarms at all were produced in the 100 µg/L group.

To estimate the potential impact of clothianidin exposure on standard-sized colonies (simulation B), we ran BEEHAVE under default settings but reduced the protein content of the jelly (*ProteinFactorNurses*). The degree to which the protein was depleted during exposure was defined by *ProteinNursesModifier_Exposed*, with values set according to the results of the previous set of simulations (Table 2). Swarming was either prevented or allowed (following the colony remaining in the hive). Colony sizes at the end of the year decreased with increasing concentrations of clothianidin, particularly when swarming was prevented (Fig. 6). No swarms at all formed at the highest clothianidin concentration. For the lower clothianidin concentrations, each colony released a single swarm during the first year, whereas the control colonies produced on average 1.2 (± 0.41) swarms in the first year.

At the end of the first year, one of the control colonies died after producing two swarms, but all other colonies survived (n = 30). When we extended the simulations over 3 years, two colonies in the 100 µg/L treatment group died after the second year and another nine died at the end of the third year (regardless of whether or not swarming was allowed, as these colonies did not swarm at all). No other colonies died after years two or three. The mean colony sizes after 3 years (without swarming) were 11,335.3 worker bees (control, SD = 1,209.9), 9,951.4 (1 µg/L, SD = 943.5), 9,225.6 (10 µg/L, SD = 1,116.6), and 3,097.1 (100 µg/L, SD = 2,427.8).

## Discussion

Brood care is fundamental to the maintenance of a honeybee colony and lies at the heart of its social interactions^25,46^. We dissected the brood care activity of a colony by measuring the HPG size of individual nurse bees, by chemical analysis of worker jelly and larval composition, by tracking the survival of individual larvae, and by monitoring the overall brood production of the colony precisely. By this means, we established small nucleus colonies as convenient pesticide testing systems, allowing us to investigate the key aspects of social interactions and the manner in which colonies compensate for external stressors. The experimental setup covered a range of field-realistic exposure levels to the neonicotinoid clothianidin, which reaches mean levels of 9.4 µg/kg (maximum 41.2 µg/kg, mean prevalence 11%) in pollen and 1.9 µg/kg (maximum 10.1 µg/kg, mean prevalence 17%) in honey^47^. In the field, a colony is likely to be exposed to such concentrations for a shorter time than our chronic exposure scenario, where colonies received clothianidin over a period of 7 weeks.

Our HPG measurements revealed that age-defined workers in all colonies exposed to clothianidin had significantly smaller acini than untreated controls. Indeed, we found a non-linear dose–response relationship of clothianidin on acini size. Non-linear dose–response relationships of toxins have been reported before and have been also described for neonicotinoids^36,37^. Smaller HPGs could lead to malnutrition, because young larvae and the queen feed exclusively on worker jelly and older larvae receive worker jelly in a suspension with honey and pollen^25^. Neonicotinoids are known to reduce the size of the HPG in individual nurse bees^28–30^ and also to reduce the levels of acetylcholine in their worker jelly^30^. We also found that the lipid composition of the worker jelly and larvae changed when colonies were exposed to clothianidin. Accordingly, the expression of larval genes involved in lipid and carbohydrate metabolism, and the lipid composition of larvae, was also shown to change when honeybee colonies were exposed to field-realistic concentrations of imidacloprid^48^. We found that neither concentration nor composition of protein in worker jelly was affected by clothianidin. However, the method we used is only sensitive enough to detect substantial changes in protein composition, and more sensitive methods such as mass spectrometry would be required to measure more subtle alterations. Only the highest concentration of clothianidin we tested (100 µg/L) caused a general decrease in protein levels on the SDS-PAGE gel, although there was no significant difference in the quantity of total extracted protein. This may indicate that high concentrations of clothianidin stimulate the activity of proteases in worker jelly. Alternatively, the general decrease of protein levels in the SDS PAGE in the 100 µg/L group could also be due to high molecular weight aggregates that are unable to enter the gel. In conclusion, even though the total protein concentration in worker royal jelly appeared to be unaffected by clothianidin exposure, there may be an impact on the overall amount of brood food produced by individual nurse bees, and this should be examined in future experiments.

Jelly is not only fed to larvae and queens but also to drones^49^, young workers and even foragers^50^. The capacity for pollen digestion enables nurse bees to exploit pollen nutrients efficiently and distribute the protein-rich food to other members of the colony, which take on more specialized tasks such as reproduction and foraging^50^. As well as the observed negative impact on brood survival a deficiency of nurse bees could indirectly affect adult workers and drones by reducing the amount of high-quality worker jelly available as food^51^. Given the key role of nurse bees in the provision of nutrition not only to the brood and queen but also all other members of the hive, there is an urgent need to study how pesticides affect their eusocial interactions.

Workers can regulate the egg-laying rate of a queen by controlling the quantity and quality of the food provided to the queen by nurse bees^52^. The undernourishment of a queen can inhibit egg laying and thus reduce the growth and survival of a colony. However, we found that the number of eggs in the colony differed little between the treatment groups, although there was a trend for higher egg numbers in colonies exposed to clothianidin. Given the 7-day intervals between assessments and the 3-day developmental period of an egg, we cannot be sure that the egg-laying rate of the queen was not affected.

The effect of neonicotinoids on brood mortality is controversial. Laboratory survival tests show that artificially reared larvae can consume large quantities of neonicotinoids (more than they would encounter under realistic field conditions) without direct lethal effects^30^. However, a reduction of the number of brood cell has been observed in some field studies^11,29,31^ which suggests that neonicotinoids may have an indirect impact on brood mortality, for example by preventing exposed nurse bees from completing their nurturing tasks effectively.

In the experimental colonies, we observed an absolute reduction in the number of brood cells only in the group exposed to highest clothianidin concentration (100 µg/L). The effect was first observed after 3 weeks of exposure. In the second phase of the experiment, the number of larvae or capped brood cells declined even further. By week 4, no larvae at all were observed in 60% of the colonies, and in the remainder only very young larvae were present (developmental stage 4–5 after egg laying). The stronger effect on brood cell numbers from week 3 onwards may reflect the dilution of the active ingredient in the first few weeks of the experiment, given the relatively large initial food stores of honey and beebread and the freedom of the bees to forage. As brood jelly production is very much dependent on pollen intake by the nurse bees, this might have contributed to a dilution of the effect in the first weeks of the experiment. In our experimental setting, the active nurse bees in the second phase of the experiment were exposed to clothianidin during their entire development and adult life, potentially having a substantial impact on their behavior. In the European Union, a honeybee brood test is required for the risk assessment of pesticides according to OECD guideline 75. Like our brood survival assessment, this test tracks the individual fate of a brood cell and determines the brood survival rate^51^. However, only one brood cycle directly after pesticide exposure is mandatory for the OECD test, whereas our data show that negative effects can be more severe in the second brood cycle.

The decreased brood survival in the clothianidin exposed colonies could either be a direct result of clothianidin on the brood that ends up in the food jelly, or an indirect effect related to brood care, e.g. on HPG function or brood care behavior of the nurse bees. We did not test the clothianidin concentration in the worker jelly. However, in laboratory studies, Böhme et al.^54^ exposed nurse bees with pesticides via a pollen and honey mixture. Subsequently, only small concentrations of these chemicals were detected in the jelly produced by these bees. The concentration varies with the pesticide and ranges between 0.001 (pyraclostrobin) to 0.016% (thiacloprid) of the active ingredient in royal jelly compared to the spiked food ingested by the bee. These results suggest that only low concentrations are delivered to queen larvae via royal jelly^54^^.^ The number of pesticides and the amount of each pesticide in worker jelly increased with larval age up to 0.1478%. This correlates with the increasing amount of contaminated pollen that is added to the jelly of older worker larvae^54^^.^ In our study we only spiked the sugar solution with clothianidin, the bees were allowed to collect non-contaminated pollen. This is an artificial situation; systemic pesticides like clothianidin are usually present in nectar and pollen of treated plant^18^. Thus, the effect on brood survival might have been even stronger, if also the pollen would have contained clothianidin.

In the small nucleus colonies, brood survival was reduced even by field-realistic concentrations of clothianidin (1 or 10 µg/L), particularly in the second phase of the experiment, where the survival of individually tracked larvae was 40% or lower. Concurrently, the absolute numbers of larvae and pupae in the exposed colonies were close to normal levels, and in some weeks even higher than in the untreated controls. This clearly shows that the colonies compensated for the increase in mortality by producing more brood. Indeed, the colonies in the 1 µg/L treatment group needed to produce 1.57-fold more larvae than the control group to maintain a stable population, and this demand increased at higher concentrations of clothianidin. The contrast between the low survival rate of the larvae and pupae yet the near normal absolute brood numbers under field-realistic exposure conditions provides evidence of a remarkable additional brood-rearing effort in the colonies exposed to the pesticide, allowing them to compensate for the negative effects of clothianidin on brood survival.

The overall costs for the colony of this compensatory brood activity are difficult to assess. First, we should consider the number of eggs produced by the queen. Only fertilized eggs develop into worker bees, so colony growth and survival depends on the ability of the queen to produce fertilized eggs. The lifetime performance of a queen is limited by the number of sperm she receives on her nuptial flights and stores in her spermatheca. In our experimental setting, a queen will never reach the limits of her egg-laying capacity, but in a full-sized colony a queen might approach her reproductive limits earlier than normal and would need to be replaced by the colony or the beekeeper. Premature queen supersedure and shorter queen lifespans are important drivers of honeybee colony mortality^7,11,37,38,53,54^. The accelerated egg-laying we observed to compensate for increased brood mortality might contribute to that problem. Second, to estimate the energetic costs of losing and replacing brood for the colony, we must consider that most of the larvae and pupae that eventually die are consumed by the workers, thus the nutrients are recycled and not completely lost to the colony. Third, it is difficult to judge the effect of the reduced brood-rearing capacity on the life traits of individual worker bees. This phenomenon can extend the lifespan of workers^55^, but more workers may get involved in brood-rearing tasks to compensate for the more limited capacity of nurse bees to produce worker jelly. We do not know how the disruption of this complex interaction affects overall colony fitness. As eusocial insects, honeybees depend on task specialization, which is precisely adjusted to the needs of the colony. The increase in brood mortality and the inability of nurse bees to produce sufficient worker jelly could easily interfere with this delicate balance with a serious impact on colony fitness.

Because our experiments involved nucleus colonies, it is unclear to what extent the observed effects of clothianidin exposure can be extrapolated to full-sized colonies. We estimated the impact on colony development in standard hives using the honeybee model BEEHAVE^44,56^, which simulates colony dynamics and agent-based foraging in realistic landscapes. The model was assessed by the European Food Safety Authority (EFSA), which concluded it would be a good starting point to model the impact of pesticides and other stressors on honeybee colonies^57,58^. Although BEEHAVE does not explicitly incorporate pesticides, it can be used to address their effects on the behavior and mortality of bees indirectly^59,60^. Accordingly, exposure in our simulations was not dependent on the foraging activities of the bees but was imposed by impairing the ability of in-hive bees to feed larvae due to the production of lower amounts of high-quality worker jelly. We observed strong effects on swarming and colony survival when we simulated clothianidin concentrations of 100 µg/L. Lower concentrations of clothianidin, closer to field-realistic doses, had little impact on overwintering colony sizes and no effect on colony survival, agreeing with previous findings reported by Cutler and colleagues^61^. Although such colonies might appear strong and healthy, we found that clothianidin had a strong negative effect on the number of swarms produced, particularly at high doses. This effect was not considered by Cutler and colleagues^61^ but was reported by Sandrock and coworkers for clothianidin and thiamethoxam^11^. Less frequent swarming would lower the resilience of bee populations in response to other stressors and make them more vulnerable to colony losses due to factors such as climate, weather, diseases or forage gaps.

The sublethal effects of neonicotinoid exposure on individual honey bees have been confirmed, but the impact on the performance and survival of colonies remains controversial^38^. Nevertheless, colony-level performance is of primary interest in managed honeybees because lower performance and higher rates of colony loss directly affect beekeepers and pollination services. The inability to substantiate the sublethal effects of pesticides and reliably detect them in field trials^40,41^ may reflect two major problems, which are discussed in turn below.

First, higher-tier field trials for the risk assessment of pesticides often lack statistical power as a result of the relatively low sampling size (colony number). Only well-resourced enterprises such as the agrochemical industry have the means to commission large-scale higher-tier field experiments. The outcome of these experiments is in most cases confidential and the data are not available for scientific or public evaluation. However, with our small nucleus colony approach, it is easy to scale up the number of colonies to achieve sufficient statistical power and to perform field trials at a fraction of the cost of semi-field/field trials with full-sized colonies, thus allowing independent research without industry funding.

Second, the ability of colonies to compensate for the impact of pesticides adds a layer of complexity to investigations looking for the sublethal effects of pesticides. A colony is a functionally organized cooperative group in which the tasks and activity levels of each individual are flexible and rapidly adjusted according to the group’s needs^62^. The large number of interacting individuals allows the colony to adaptively reallocate work and resources to compensate for potential negative effects on individuals. This plasticity enables the colony to react adaptively to environmental stressors, masking the effects on individuals. Indeed, the ability of a honeybee colony to withstand or recover quickly from disturbances is known as resilience, which is an intrinsic property of many complex systems. However, events leading to loss of resilience in complex systems are rarely predictable and are often irreversible^63^. In honeybee colonies, this point arrives when the compensatory capacity of the colony reaches its limits, triggering the sudden breakdown of a seemingly healthy colony, especially when multiple stressors act together.

As a super-organism, a honeybee colony can compensate for many stressors due to the presence of many individuals and the complex social interactions among them^55^. With our small nucleus colony approach, we reduced the number of bees but maintained the social coherence of the colony^64^, allowing us to investigate the compensatory capacity of the colony in detail. This sensitive model system allows the assessment of single or multiple stressors on the compensatory capacity of the colony, and will help to close the knowledge gap between laboratory cage experiments and field trials with full-sized colonies. This will facilitate not only scientific investigations, but also the regulatory testing for new pesticides.

## Methods

### Clothianidin exposure in field experiments

The experimental bee yard was situated in a suburban area in Kirchhain (Germany) with sufficient natural pollen sources in late summer, where the honeybees were allowed to forage freely. The field experiment took place with 20 *Apis mellifera carnica* colonies in Kirchhainer mating nuclei (25.5 × 19.8 × 17 cm), established with sister queens (*A. m. carnica* breeding line, mother: 17:27:20:11) mated on an island mating station (Norderney) and 180 g of worker bees each. We tried to reduce the variance between colonies by using young sister queens from an island mating station. Furthermore, we used small hives to minimize food competition so that we were able to set up the colonies within a rather small area of 20 by 20 m. In detail, the worker bees originated from four healthy colonies of the institute, located at an apiary of the institute. In order to get a homogenous composition, worker bees of brood combs and honeycombs of two colonies were shaken into a box and mixed thoroughly, before they were uniformly distributed into 10 mating nuclei. This procedure was repeated to fill 20 mating nuclei. Subsequently, the mating boxes were placed in a cool room (12 °C) for three day, to acclimatize, before they were transported to the island mating station Norderney. The queens started to lay eggs at June 25, 2014. The clothianidin exposure started at July 28. Thus, the colonies containing freshly mated, egg-laying queens were allowed to establish and build up an intact brood nest for four weeks. Two days before clothianidin exposure (sampling day 0, S0; SFig. 1), the colony strength was assessed and the treatment groups were randomly assigned to the hives such that differences between treatment groups were minimized with respect to the strength of their colonies. Every week, from July 30 to September 10, each colony received 400 mL of Apiinvert (Südzucker AG, Mannheim, Germany) sugar syrup (39% w/v fructose, 31% w/v saccharose and 30% w/v glucose) spiked with clothianidin (1, 10 or 100 µg/L). Control colonies received sugar syrup containing the same concentration of the solvent (water) as the clothianidin-treated groups. The syrup was fed in zip-look bags placed inside the food chamber containing a climbing aid. After 1 week, the leftovers were removed and weighed to record food consumption. For all four experimental groups, the analyzed clothinidin levels were close to the target concentrations (Suppl. Table 1). Environmental data were recorded during the study period using a USB data logger (EL-USB-2, Lascar Electronics Ltd., temperature accuracy ± 0.5 °C, relative humidity accuracy ± 3%) located under the colonies.

### Sampling

During weeks 3 and 7, random samples of worker bees, larvae and worker jelly were taken from each colony. In detail, 20 randomly chosen worker bees located on a brood comb and five larvae (larval stage: day 7 or 8 after egg laying) were immediately frozen for chemical analysis. To collect worker jelly, extra thick blotting paper (Protean, Xi size; Bio-Rad, Hercules, CA, USA) was cut into strips, cleaned in pure ethanol and acetone (Carl Roth, Karlsruhe, Germany) and dried in a heating cabinet at 80 °C. Each strip was inserted into five brood cells containing a small larva (developmental day 4–5) to suck up the worker jelly and the strips were immediately frozen.

To document brood development, each side of each comb was photographed within an empty hive box transformed into a photo box containing a digital camera (Canon PowerShot A1000 IS, Tokyo, Japan) and a ring-flash (Aputure Amaran AHL-C60 LED, Shenzhen, China). Brood documentation took place every week. The colonies were sampled starting with the control and then from the lowest to highest concentration of clothianidin to minimize the risk of carryover.

### HPG size measurements

To obtain worker bees of a defined age, single frames of late-stage capped brood (Binder, Tuttlingen, Germany) were brought to the laboratory and incubated in the dark at 32 °C, with humidity provided by open water jars. The frames with worker brood were collected from two full-sized colonies*,* which were regularly inspected for symptoms of disease and tested for Chronic bee paralysis virus, Deformed wing virus, Acute bee paralysis virus, and Sac brood virus^65^. Newly-emerged bees (≤ 24 h) were collected, color marked, and transferred to the experimental colonies on July 28 (15 marked bees per colony). During the second week of exposure, the marked bees (Suppl. Table 2) were removed from the colonies after 12 days in the hive and immobilized on ice. The HPGs were dissected in ice-cold phosphate-buffered saline (PBS, pH 7.4). The specimens were fixed in formaldehyde (4% in PBS, Carl Roth), rinsed three times in PBS, and mounted in Aquapolymount (Polysciences, Eppelheim, Germany). Three pictures of each gland (only one gland per bee) were photographed at 400x magnification using a phase contrast/fluorescence microscope (Leica DMIL, Leica camera DFC 420C) and LAS v4.4 image-capturing software (Leica Microsystems, Wetzlar, Germany). To measure the size of each gland, the diameters of 30 acini per bee were measured using ImageJ v1.49o (http://rsb.info.nih.gov/ij/index.html).

### High-performance thin-layer chromatography

HPTLC was chosen as sensitive analytical method in order to detect qualitative and semi-quantitative differences in the composition of brood food and larvae of dosed hives. Individual larvae (one per colony) differing in their weights (200–500 mg/larva) were solely macerated and extracted in 1 mL *n*-hexane (> 99% pure, Rotisolv, Carl Roth) in an ultrasonic bath for 1 min and then vortexed for 1 min. For the analysis of worker jelly (from three cells per colony), adsorptive filter strips (Sugi strips, Kettenbach, Eschenburg, Germany) were cut in half and one part was dipped in brood combs until maximal absorption of the material, whereas the other strip was used as background control strip, and both strips were extracted as above. The supernatants of larvae and worker jelly were transferred to a fresh vial 1000 μL isopropylacetate/methanol (3/2, v/v). After maceration for 1 min, the mix was vortexed for 1 min and the supernatant was transferred to another vial. Between extractions, the samples were cooled on ice and stored at –20 °C.

The isopropylacetate/methanol extracts (20 µL/band for worker jelly and 7 µL/band for larvae) were sprayed onto the silica gel 60 F_254_ HPTLC plate (Merck, Darmstadt, Germany) using an Automatic TLC Sampler 4. The plate of worker jelly was developed with a mobile phase consisting of chloroform/methanol/water/ammonia (30/17/2/1, v/*v*/*v*/*v*, all Carl Roth^66^ and the plate of larvae with an 8-step gradient development based on methanol, chloroform, toluene and n-hexane^66,67^. After drying in a stream of cold air for 2 min, the plate images were documented at UV 366 nm using the TLC Visualizer. For derivatisation, the chromatogram was dipped into the primuline solution (100 mg primuline in 200 mL acetone/water, 4/1 *v*/*v*, Sigma-Aldrich, Steinheim, Germany) at an immersion speed of 2.5 cm/s and an immersion time of 1 s, dried and derivatised as before. The data were processed using winCATS version 1.4.2.8121 (all instrumentation from CAMAG).

The bacterium *Aliivibrio fischeri* (NRRL-B11177, strain 7151), obtained from the German Collection of Microorganisms and Cell Cultures (DSMZ, Leibniz Institute, Berlin, Germany), was used to assess a non-targeted, broad range of effective substances within the worker jelly. HPTLC plates were developed as described above, neutralized and immersed in a bacterial suspension, prepared according to DIN EN ISO 11,348–1 8^45^ at an immersion speed of 3 cm/s and an immersion time of 2 s. The bioluminescence of the wet bioautogram was recorded in an interval of 3 min over 30 min using the BioLuminizer (CAMAG).

### Brood assessment

The free extension of the open source program ImageJ, Fiji (http://fiji.sc/Fiji) was used to count all cells with eggs, larvae, or sealed brood, for every colony on every sampling day. The photos of a single comb from different sampling days were aligned in the program and all brood cells were counted. Because the colonies had no wax foundations, some cells on the edges of these naturally-built combs were at an unfavorable angle. Therefore, most but presumably not all cells with brood were visible in the photos. This uncertainty was similar in all hives. To estimate the brood survival, we first tracked the development of individual eggs, but found a high mortality rate even in control colonies. Therefore, we tracked the development of individual larvae (day 4–5 after egg laying) over 4 weeks (= sampling weeks). Brood survival was estimated for two periods during the experiment (weeks 1–4 and 3–7).

### Modeling of demographic compensation

The aim of the model was to estimate the number of new larvae that must hatch each week in order to maintain a stable number of larvae up to 7 weeks of age given the survivorship schedule of larvae week by week. Let *h* denote the number of eggs that hatch into larvae each week, and let the probability that any individual larva dies in each successive week be *m*_*i*_, where *i* takes values in the set {1, 2, …, 7} to indicate each of seven successive weeks, after which we assume that surviving larvae pupate. We used a demographic matrix model^68^ to describe the state of the population of larvae each week as follows. Let *l*_*x*_ denote the number of larvae in the colony that are aged *x* weeks post-hatching and let *m*_*x*_ denote their *per capita* weekly mortality rate. The population of larvae is distributed into seven age classes and we also assign a class to queens, which give rise to larvae by producing eggs. Using the Lefcovitch matrix approach, the larval population of a colony can thus be viewed as a state vector *n*_*t*_ whose week-by-week change is the product of a matrix *A* and the population state vector ***n***_***t***_, as shown in Eq. (1):

1 $$An_{t} = \left[ {\begin{array}{*{20}c} 0 & 0 & 0 & \ldots & 0 & {\varvec{h}} \\ {(1 - {\varvec{m}}_{1} )} & 0 & 0 & \ldots & 0 & 0 \\ 0 & {\left( {1 - {\varvec{m}}_{2} } \right)} & 0 & \ldots & 0 & 0 \\ \vdots & \vdots & \vdots & \ddots & \vdots & \vdots \\ 0 & 0 & 0 & \ldots & {\left( {1 - {\varvec{m}}_{7} } \right)} & 0 \\ 0 & 0 & 0 & \ldots & 0 & 1 \\ \end{array} } \right]\left[ {\begin{array}{*{20}c} {{\varvec{l}}_{1} } \\ {{\varvec{l}}_{2} } \\ {{\varvec{l}}_{3} } \\ \vdots \\ {{\varvec{l}}_{7} } \\ {\varvec{Q}} \\ \end{array} } \right]$$

The lowermost element of *n*_*t*_ is the number of queens (*Q*) which is here set to *Q* = 1 for all models, and the total number of larvae in the colony at any time is ${\varvec{L}}_{{\varvec{t}}} = \mathop \sum \nolimits_{{\varvec{x}}} {\varvec{l}}_{{\varvec{x}}}$. Given the observed mortality schedule (*m*_*x*_), we used the model to solve for the value of *h* that produces a stable value for *L*_*t*_, as shown in Eq. (2):

2 $$An_{t} = n_{t}$$

We determined the mortality schedule by observing the survivorship of a larval cohort. For example, if a cohort of *S*_*t*_ larvae was originally marked at time *t* and, of these, *S*_*t*+1_ survived until the following week, the *per capita* weekly mortality rate would be estimated as shown in Eq. (3):

3 $${\varvec{m}}_{{\varvec{t}}} = 1 - \frac{{{\varvec{S}}_{{{\varvec{t}} + 1}} }}{{{\varvec{S}}_{{\varvec{t}}} }}$$

### BEEHAVE simulations

To predict the impact of clothianidin on colony development in standard hives we used BEEHAVE, a honeybee model that simulates colony dynamics and agent-based foraging in realistic landscapes^44^(http://beehave-model.net/). Although BEEHAVE does not explicitly allow the incorporation of pesticides, the effect of pesticides on behavior and mortality can nevertheless be addressed^59,60^. BEEHAVE simulates the development of a single honeybee colony, starting with 10,000 foragers on January 1. Colony dynamics are based on a daily egg laying rate, with the developmental stages eggs, larvae, pupae, and adults (in drones) or in-hive workers and foragers (in workers). The brood needs to be tended by in-hive bees, and the larvae additionally need to be fed with nectar and pollen. Foragers can scout for new food sources or collect nectar and pollen from sources already known. Successful foragers can recruit nestmates to the food source. Mortality rates depend on the developmental stage and the time spent on foraging. The colony dies if it either runs out of honey or if the colony size falls below 4000 bees at the end of the year. Swarming may take place when the brood nest grows to more than 17,000 bees before July 18. Under default conditions, two food sources are present at distances of 500 or 1,500 m. Daily foraging conditions are based on weather data from Rothamsted, UK.

We ran two sets of simulations: (A) We first set up BEEHAVE to mimic our experimental nucleus colonies. We then determined how the protein content of the jelly produced by nurses (*ProteinFactorNurses*) had to be modified to replicate the larval mortality we observed in our empirical data. (B) We then set up BEEHAVE under default conditions but modified *ProteinFactorNurses* according to the results from the previous simulations to assess the impact of clothianidin exposure under more realistic conditions.

To mimic the experimental nucleus colonies (simulation A), the maximum honey store (*MAX_HONEY_STORE_kg*) was reduced to 0.77 kg and the maximum size of the brood nest (*MAX_BROODCELLS*) was reduced to 250. Furthermore, *HoneyIdeal* was set to ‘true’, so that even though the honey store was small it was filled every day, reflecting the feeding of the experimental bees. In contrast, *PollenIdeal* was set to ‘false’, because the experimental colonies still had to forage for pollen. On day 209 (July 28), we set the number of pupae to 100 and the number of workers to 650, similar to the experimental colony sizes. During the exposure to clothianidin between days 211 (July 30) and 253 (September 10), the protein content of the jelly fed to the larvae (*ProteinFactorNurses*) was modified by the new variable *ProteinNursesModifier_Exposed*. We tested for *ProteinNursesModifier_Exposed* values from 0.6 to 1 in steps of 0.01. The main output of the simulation was the survival of the brood, calculated from the brood cohort sizes aged 19 days divided by the sizes of these cohorts when they were 3 days old. We calculated the mean brood survival over 30 replicates, using the last 10 cohorts only (i.e. those reaching the age of 19 days between September 1 and 10). Those parameter values for *ProteinNursesModifier_Exposed* resulting in brood survival most similar to the experimental brood survival were then chosen to represent the clothianidin concentrations of 100, 10 and 1 µg/L.

To assess the impact of clothianidin on standard colonies under more realistic conditions (simulation B), we ran BEEHAVE under default settings but reduced the protein content of the jelly (*ProteinFactorNurses*) during times of exposure. We assumed that colonies would be exposed when rapeseed plants are flowering, defined in the model as the period between days 95 (April 5) and 130 (May 10). *ProteinNursesModifier_Exposed* values representing the tested concentrations of clothianidin were derived from the previous set of simulations, and for the control we set *ProteinNursesModifier_Exposed* to 1 (i.e. no effect of clothianidin). Swarming was either prevented or allowed, in which case the simulation followed the colony remaining in the hive.

### Statistical methods

Statistical analysis was carried out using *R* v3.4.2^69^, including the add-on packages *lme4*^70^ for linear mixed-effects models, *pbkrtest*^71^ for testing fixed effects in mixed-effects models, *parallel*^69^ to increase computational power, *RLRsim*^72^ for testing random effects in mixed-effects model, *multcomp*^73^ for multiple comparisons, and *lattice*^74^ for various graphical displays. We used linear mixed-effects models for one- and two-factorial analysis of variance (ANOVA) or regressions as indicated and where necessary. In those models, the colonies were modeled as random effects to reflect the (longitudinal) grouping structure in the data. For the analysis of larval survival, we used a logarithmic transformation of the proportion of surviving larvae. When testing fixed effects in mixed-effects models, we used the Kenward-Roger method (and double-checked the results by comparing them with parametric bootstrap values). Where sufficient, we simplified the analysis using linear (fixed-effects) models. Model diagnostics were performed for all fitted models using qualitative tools such as normal q-q-plots for residuals and plots of residuals versus fitted values to assess the validity of model assumptions like homoscedastic normality. Dunnett’s test (or customized contrasts as appropriate) was used for multiple comparisons in post hoc analysis, and *P* values were appropriately adjusted for multiple testing within well-defined test families using the single-step or Westfall’s method. Model and analysis details, model diagnostic graphs, and further information are available in the supplemental statistical report.

## Supplementary information

Supplementary Information 1.
