# Supplementary material for: Honeybee colonies compensate for pesticide-induced effects on royal jelly composition and brood survival with increased brood production
Source: Sci Rep. 2021 Jan 8;11:62. doi: 10.1038/s41598-020-79660-w (PMC7794607; doi:10.1038/s41598-020-79660-w)
Supplement: Supplementary file 1 — Supplementary Information 1. [file 41598_2020_79660_MOESM1_ESM.pdf]

Supplemental information for

Honeybee colonies compensate for pesticide-induced effects on royal jelly composition and brood survival with increased brood production

Matthias Schott[1, 2], Maximilian Sandmann[3], James E. Cresswell[4,] Matthias A. Becher[5], Gerrit Eichner[6], Dominique Tobias Brandt[7], Rayko Halitschke[8], Stephanie Krueger[9], Gertrud Morlock[9], Rolf-Alexander Düring[10], Andreas Vilcinskas[1], Marina Doris Meixner[3], Ralph Büchler[3], Annely Brandt[3*]

[1]Institute of Insect biotechnology, Justus-Liebig-University Giessen, Giessen, Germany;
[2]Animal Ecology I,University of Bayreuth Universitaetsstr. 30, D-95447 Bayreuth
[3]LLH Bee Institute, Erlenstr. 9, 35274 Kirchhain, Germany;
[4]Biosciences, University of Exeter, Exeter, EX4 4PS, UK;
[5]Environment & Sustainability Institute, Biosciences, University of Exeter, Penryn Campus, Penryn, Cornwall TR10 9FE, UK;
[6]Mathematical Institute, Justus-Liebig University of Giessen, Giessen, Germany;
[7]Institute of Pharmacology, Biochemical-Pharmacological Centre (BPC), University of Marburg, Karl-von-Frisch-Str. 1, 35032 Marburg, Germany;
[8]Department of Molecular Ecology, Max Planck Institute for Chemical Ecology, Hans Knöll Str. 8, 07745Jena, Germany;
[9]Chair of Food Science, Institute of Nutritional Science, Justus Liebig University Giessen, Heinrich-Buff-Ring 26-32, 35392 Giessen;
[10]Institute of Soil Science and Soil Conservation, Justus-Liebig-University of Giessen, Heinrich-Buff-Ring 58, 35392 Giessen, Germany

***Corresponding author**

Annely Brandt

Bee Institute Kirchhain

Erlenstr. 9,

35274 Kirchhain, Germany

E-mail: annely.brandt@llh.hessen.de

Tel.: +49 6422 940632, Fax: +49 6422 940633

Content

SFig 1. Sampling scheme…………………………………………………………………...Page 3

SFig 2. Climate data………………………………………………………………………. Page 4

SFig 3A. HPTLC plate from Fig2 showing the *A. fisherii* antimicrobial activity test…..Page 5

SFig 3B. HPTLC Peak areas of SFig3A……………………………………………………...Page 5

SFig 3C. Full TLC Plate of Fig2A…..………………………………………………………… Page 6

SFig 3D. Peak Profile of Fig 2A……………………………………………………………….Page 7

SFig 3E. Full HPTLC Plate of Fig2B..…………………………………………………….....Page 8

SFig 3F. Peak Profile of Fig 2B………………………………………………………………. Page 8

SFig 3G. Full HPTLC plate of Lipid profiles of larvae………...……………………………..Page 9

SFig 3H. Peak Profile of Fig 2C..…………...………………………………………………Page 9

SFig 4. Protein composition of worker jelly……………………………………………….. Page 10

Suppl. Table 1. The clothianidin concentrations in spiked sugar syrup and workers.. Page 11

Suppl. Table 2. 1 Age-defiend worker bees retrieved from the colonies……………….. Page 13

Statistical Report………………………………………………………………………….. Page 14

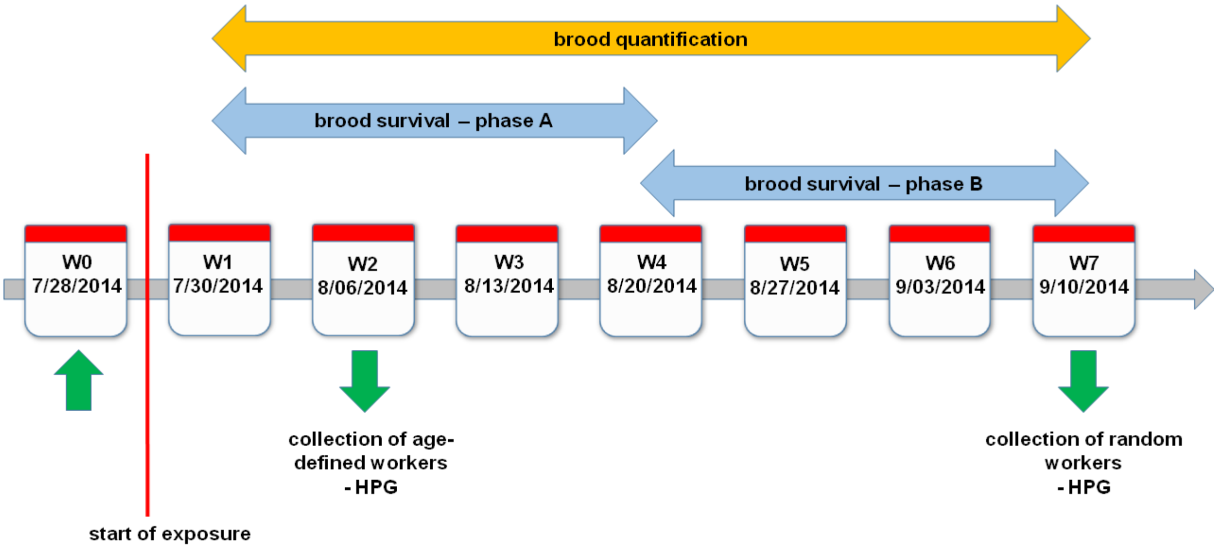

SFig 1. Sampling scheme. Each week (W1–7), all combs were photographed and samples of worker bees, larvae, and worker jelly were taken. To determine the size of the hypopharyngeal gland, 15 newly-hatched and marked worker bees were introduced into each colony during week 1 and recovered in week 2 (age-defined marked bees, green arrows). In week 7, randomly chosen worker bees of undefined age were taken from brood combs for the same reason (random bees). The total number of eggs, larvae and pupae were recorded for every week and colony (brood quantification). To determine the survival rate of individually tracked larvae, at least 50 individual cells per colony were followed over 4 weeks in the first half of the experiment (first brood cycle, brood survival phase A) and in the second half of the experiment (second brood cycle, brood survival phase B).

Climate recordings

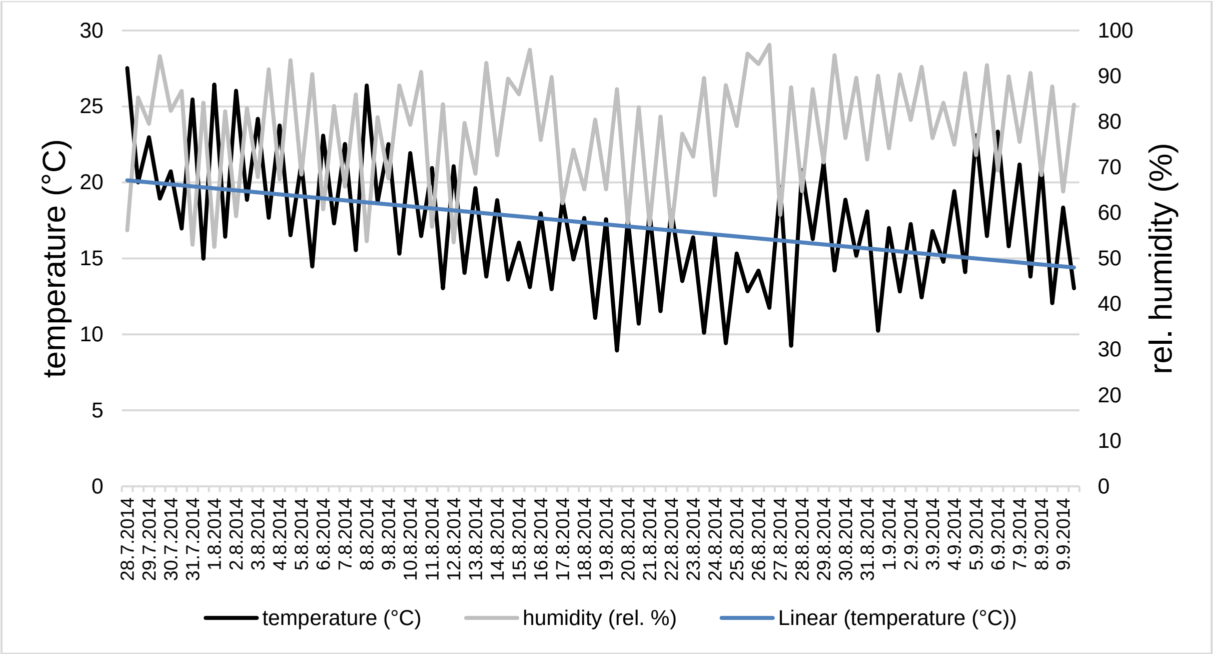

SFig 2. Climate data. Throughout the study period (July 28 to September 10, 2014), the temperature (°C) and relative humidity (%) were recorded twice daily (8:05 a.m. and 8:05 p.m.). A data logger recorded the temperature and relative humidity within the colonies over the study period. The daily temperature varied between 14.2 and 27.5 °C (8:00–20:00 h) and between 8.9 and 20 °C overnight (20:00–8:00 h). The relative humidity was 52.6–92.6% during the day and 73.8–96.9% overnight. The 20 colonies were all placed within an area of ~20 m^2. The control and treatment groups were randomly distributed within these colonies and were thus exposed to very similar environmental conditions.

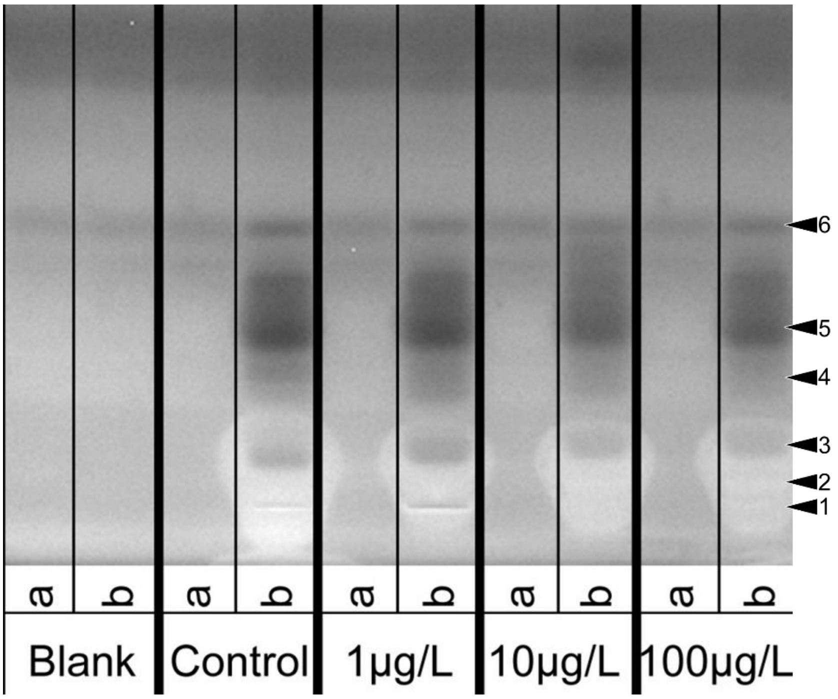

SFig 3A. HPTLC plate from Fig2 showing the *A. fisherii* antimicrobial activity test. Each Sugi strip was cut halved to provide one part without the sample (a) and another with the sample (b). The blank is a fresh Sugi strip from the same batch as the strips used in the experiment, cut into equal halves. The other lanes show the clothianidin doses (Control = no clothianidin). Arrows indicate Peaks. For peak areas see SFig2B.

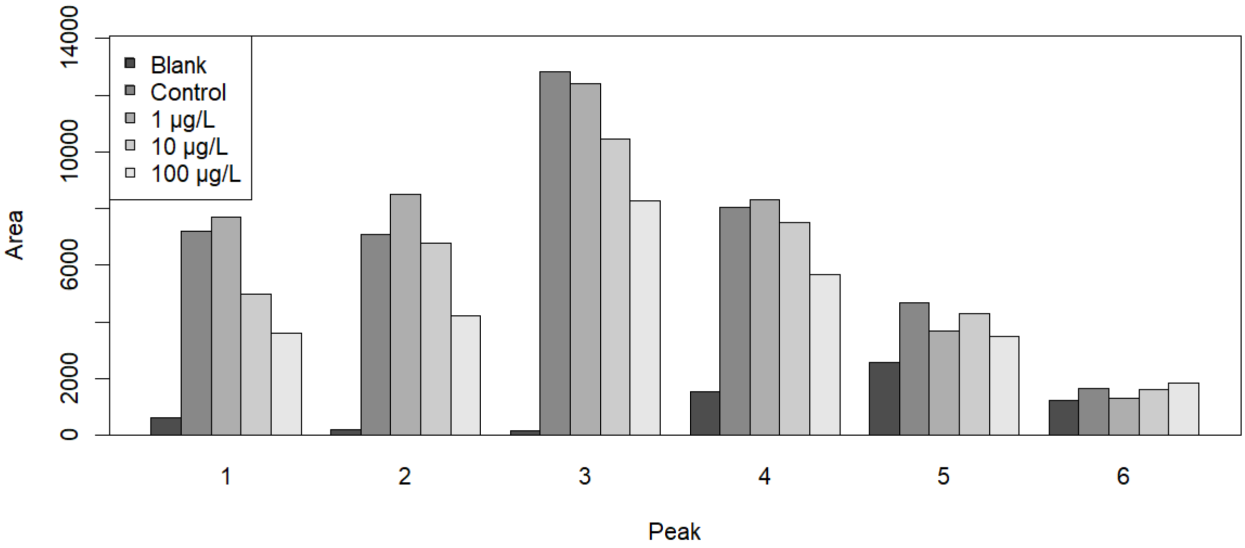

SFig 3B. HPTLC Peak areas of SFig3A. HPTLC analysed according to by Olech et al. 2012 (Olech, M., Komsta, Ł., Nowak, R., Cieśla, Ł. & Waksmundzka-Hajnos, M. Investigation of antiradical activity of plant material by thin-layer chromatography with image processing. Food Chemistry 132, 549–553 (2012)) and evaluated by Popovic and Sherma2014 (Popovic, N. & Sherma, J. Comparative Study of the Quantification of Thin-Layer Chromatograms of a Model Dye

Using Three Types of Commercial Densitometers and Image Analysis with open source program ImageJ v1.49o (http://rsb.info.nih.gov/ij/index.html). Trends Chromatogr. 9, 21–28 (2014)) with the only difference that we took photos with a TLC vizualizer from CAMAG instead of a mobile phone on a tripod.

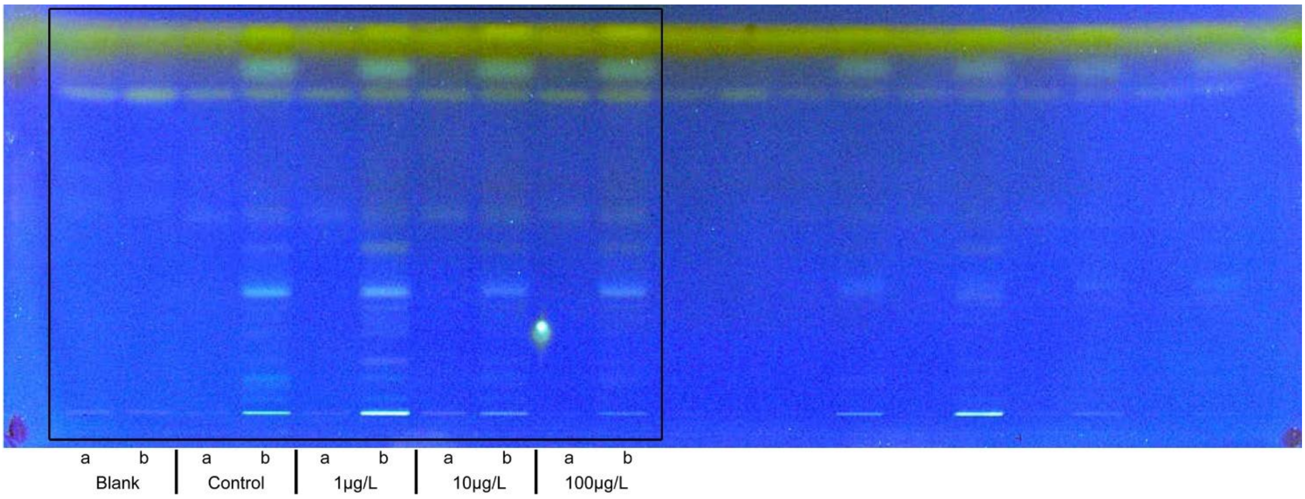

SFig 3C. Full TLC Plate of Fig2A. Lipid profiles of royal jelly samples at 366 nm, derivatization with primuline. Box indicates the area of Fig2A.

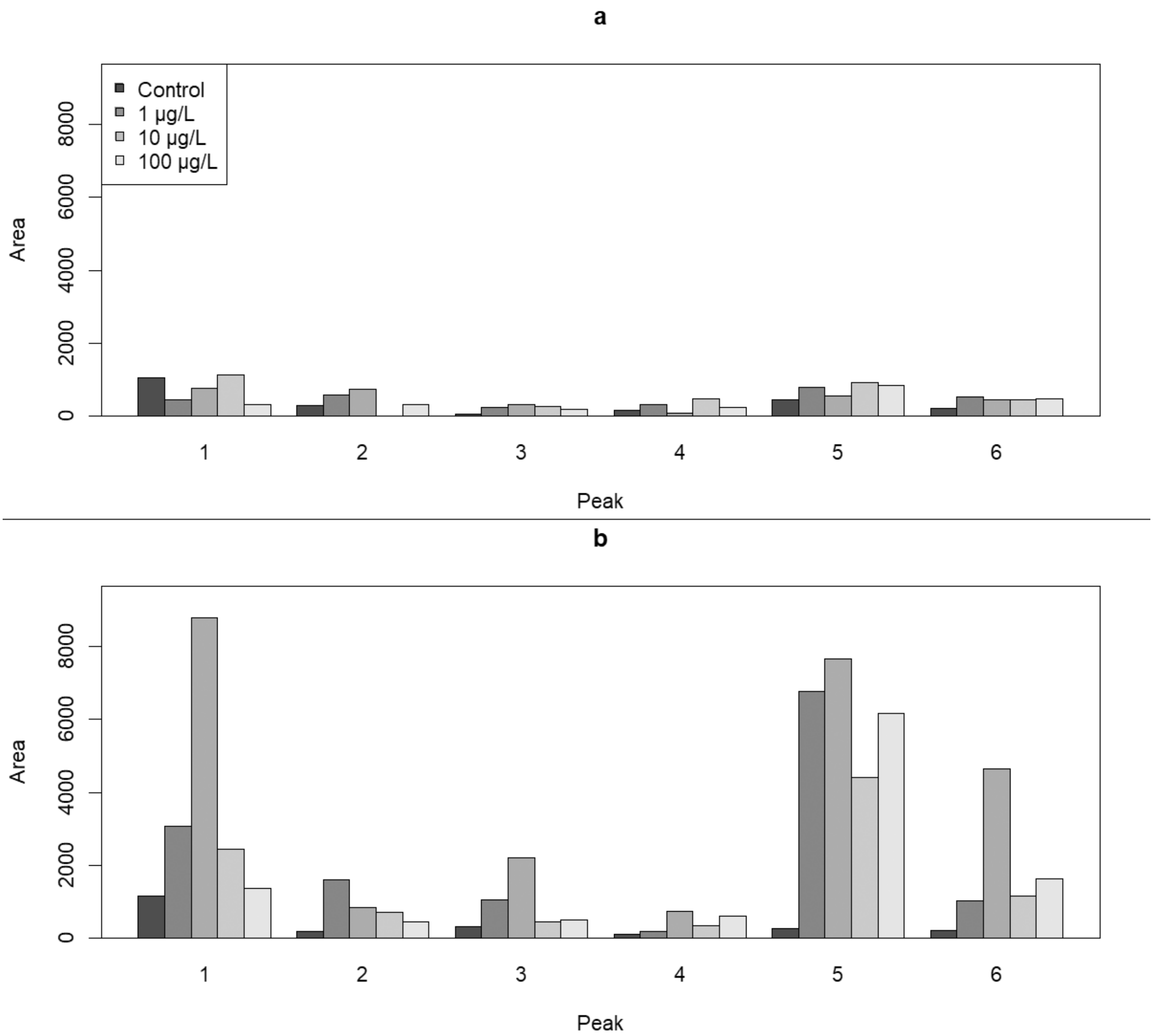

SFig 3D. Peak Profile of Fig 2A. Samples of worker jelly were taken using absorptive filter strips (Sugi strips), extracted with *n*-hexane and separated by HPTLC. Each Sugi strip was halved: one half without the sample (track a, upper plot) and the other with absorbed sample (track b, lower plot). Numbers on abscissae indicate the peaks shown in Fig. 2A.

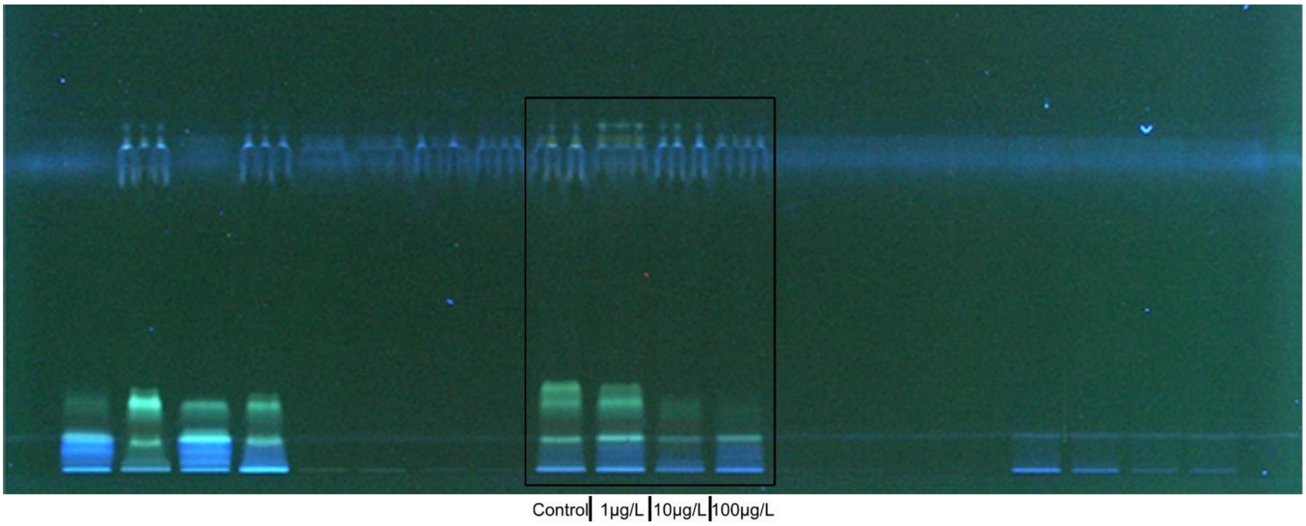

SFig 3E. Full HPTLC Plate of Fig2B. Lipid profiles of larvae at 366 nm, not derivatized. Box indicates the area of Fig2B.

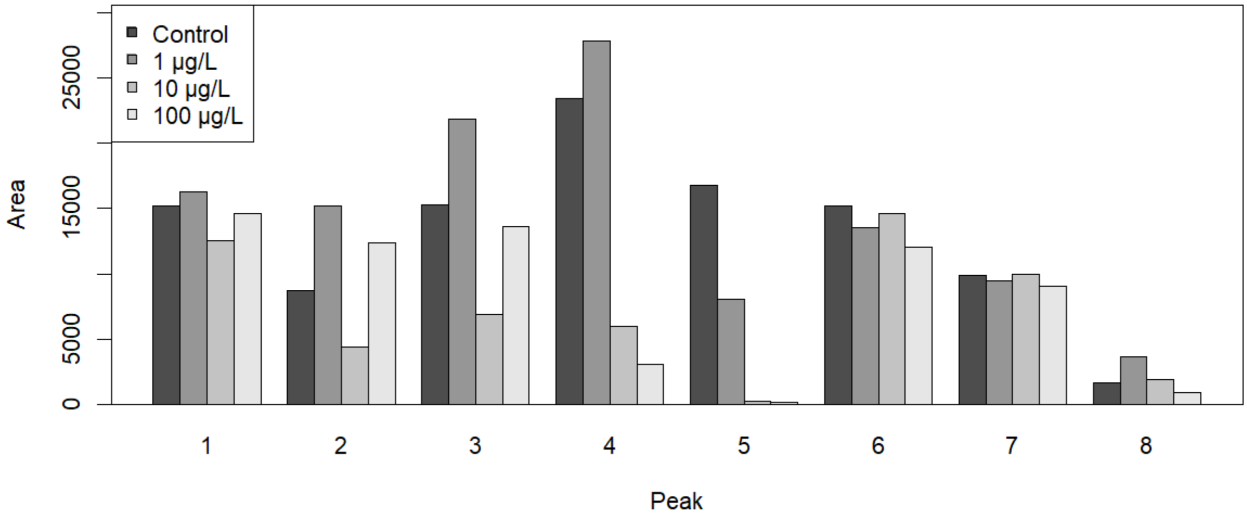

SFig 3F. Peak Profile of Fig 2B. Lipid profiles of larvae, not derivatized. Numbers on abscissae indicate the peaks shown in Fig 2B.

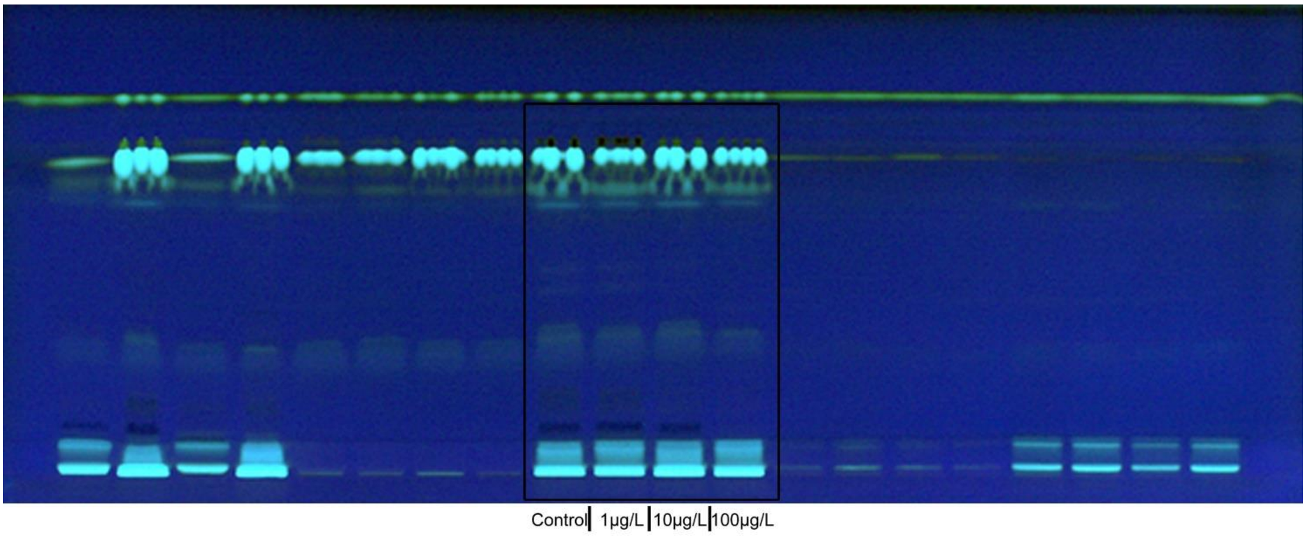

SFig 3G. Full HPTLC plate of Lipid profiles of larvae. Same plate as Fig3E at 366 nm after derivatization with primuline. Box indicates the area of Fig2C.

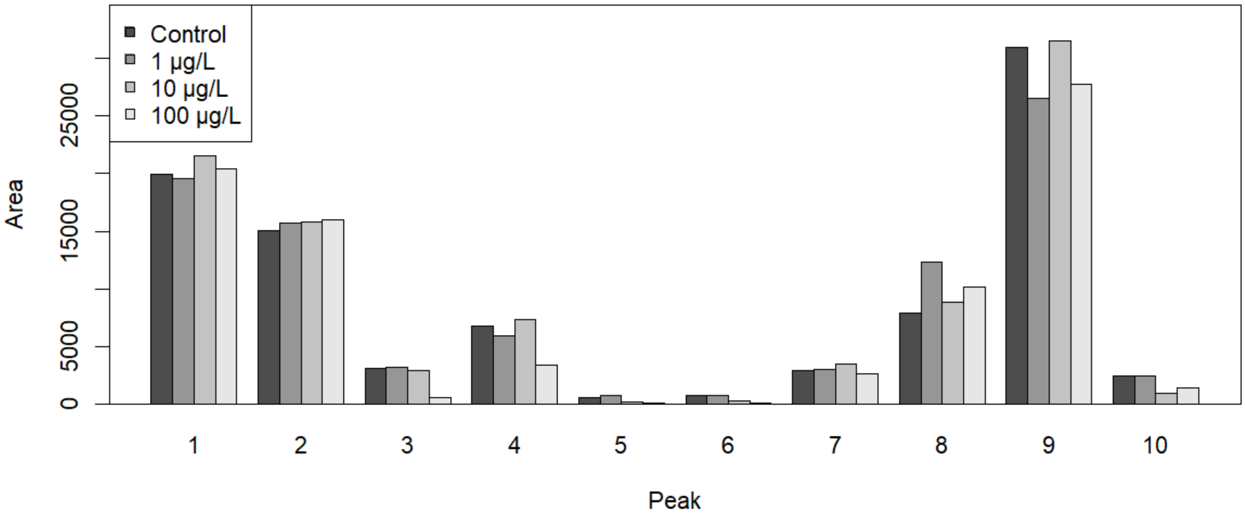

SFig 3H. Peak Profile of Fig 2C. Lipid profiles of larvae derivatized. Numbers on abscissae indicate the peaks shown in Fig. 2C.

Protein analysis of worker jelly

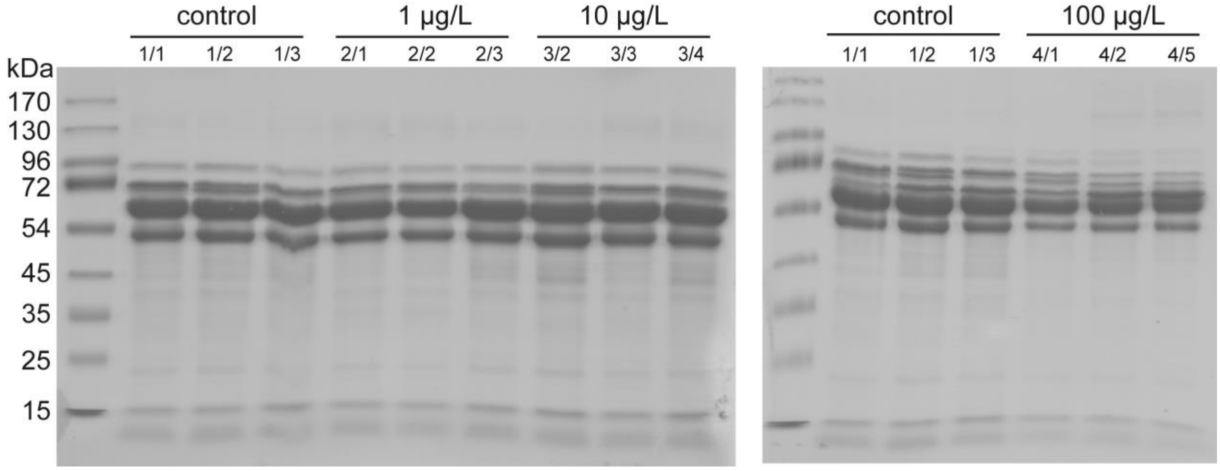

SFig 4. Protein composition of worker jelly.

For protein analysis, samples of three colonies of each treatment group were analyzed from week 4. Blotting strips were dipped in brood combs and proteins were extracted by incubation in 100 µl RIPA high salt buffer (50 mM Tris HCl pH 7.6, 500 mM NaCl, 10 mM MgCl2, 2 mM EDTA, 1% Triton X-100, 0.25% sodium deoxycholate, 0.1% SDS and Roche EDTA-free Protease cocktail mix) for 30 min at 4 °C. The protein concentration was determined using the DC Protein assay kit (Biorad; mean concentrations: control: 12.82 µg/µL ± 0.17; 1 µg/L clothianidin: 11.97 µg/µL ± 0.21; 10 µg/L clothianidin: 12.68 µg/µL ± 0.13; 100 µg/L clothianidin: 11.67 µg/µL ± 0.31). No statistical significant differences regarding the total protein concentration were found between the treatment groups (Kruskal-Wallis test, p = 0.9832).

To test, whether the concentration of a protein subfraction could be affected in clothianidin exposed bees, 15 µg of protein of each sample were analyzed by SDS-PAGE in 12% polyacrylamide gels followed by staining with Coomassie Brilliant Blue. The concentration and composition of major proteins in worker jelly was unaffected by clothianidin exposure. Although, there was no statistically significant difference in the protein concentration (see above), we observed a decrease in the concentration in major worker jelly proteins for the 100 µg/L treatment group. This could be related to a possible increase of protein degradation in this treatment group. This would lead to an increase of peptides, which appear in the total protein measurement, but are too small to be visualized on this gel. Alternatively, the general decrease of protein levels in the SDS PAGE in the 100 µg/L group could also be due to high molecular weight aggregates that are unable to enter the gel.

Clothianidin uptake

Suppl. Table 1. The clothianidin concentrations in spiked sugar syrup were close to the target concentrations, residues were detected in worker bees.

**treatment group**	**clothianidin (µg/L) in sugar syrup** (mean ± SEM)	**clothianidin in workers (ng/bee)** (mean)
control	0.0 ± 0.00	0.00
1 µg/L	1.2 ± 0.11	0.05
10 µg/L	10.1 ± 0.29	0.20
100 µg/L	99.8 ± 0.70	2.63

We analyzed the sugar syrup used to feed the experimental colonies to assess its exact clothianidin content. For all four experimental groups, the final spiked pesticide levels were close to the target concentrations (**Suppl. Table 1**). The consumption of the spiked syrup was recorded every week to estimate the total clothianidin uptake over the study period. The provided amount of 400 mL (= 540 g) syrup each week per colony was completely consumed each week with the exception of week 6 in the highest concentration treatment. All five colonies in the 100 µg/L clothianidin treatment group were visibly weakened and did not consume all the sugar syrup, which was provided during week 6 (residual syrup: 76.7, 114.5, 142.2 and 18.0 g, respectively). To determine the clothianidin levels in bees exposed to the different pesticide levels, 10 randomly chosen worker bees from each hive were analyzed on week 7 (**Suppl. Table 1**). Clothianidin was detected in the bee sample from only one colony of the control group (colony I, 0.004 ng/bee).

Analytical Method

LC/MS/MS was used for the identification and quantification of the substances in the samples. The system used was a Prominence UFLC XR HPLC (SHIMADZU) coupled to a triple quadrupole mass spectrometer 4000 Q TRAP (AB SCIEX) equipped with an electro spray ionization (ESI) source. Clothianidin and its metabolites clothianidin-metabolite TZMU and clothianidin-metabolite TZNG were identified by their retention time and three MRM transitions. The residues in the samples were quantified with reference standards in matrix (concentrations: 0.1, 0.5, 1, 5, 10, 25, 50 and 100 pg/µL). The quantification was carried out by the internal standard method. The values shown for

the samples are averages of measurements out of duplicate injections of the sample extracts. The limit of detection (LOD) was determined as the lowest concentration tested in which the peak signal of the main MRM, which was used for quantification, was three times higher than the background noise of the chromatogram. The LOD was 0,5 pg/µL for clothianidin, 1 pg/µL for clothianidin-metabolite TZMU and 5 pg/µL for clothianidin-metabolite TZNG.

Number of age-defined worker bees collected for HPG measurements

Suppl. Table 2. 1 Age-defiend worker bees retrieved from the colonies. At the beginning of the experiment, 15 newly hatched marked worker bees were introduced into each colony. At the age of 12 days, all marked bees were collected and frozen for HPG preparations. The number of retrieved worker bees differed between colonies. All HPGs were dissected at the same day. Therefore, a maximum of six bees per colony were included in the subsequent analysis.

treatment group	colony	worker bees
control	I	7
	II	8
	III	6
	IV	7
	V	7
1 µg/L	I	2
	II	6
	III	5
	IV	6
	V	3
10 µg/L	I	6
	II	1
	III	0
	IV	6
	V	6
100 µg/L	I	6
	II	6
	III	6
	IV	0
	V	5

Technical report for the statistical analysis of "Honeybee colonies compensate for pesticide-induced effects on royal jelly composition and brood survival with increased brood production"

by

Dr. Gerrit Eichner
Mathematical Institute
Justus-Liebig-University Giessen
Arndtstr. 2, 35392 Giessen, Germany
Email: gerrit.eichner@math.uni-giessen.de
URL: http://www.uni-giessen.de/eichner

and

Dr. Matthias Schott
Animal Ecology I
University of Bayreuth
Universitaetsstr. 30, 95447 Bayreuth, Germany
and
Institute of Insect Biotechnology
Justus-Liebig-University Giessen,
Heinrich-Buff-Ring 26 - 32, 35392 Giessen, Germany
Email: matthias.schott@uni-bayreuth.de

September 20, 2020

This is a report of the statistical analyses of the article "Honeybee colonies compensate for pesticide-induced effects on royal jelly composition and brood survival with increased brood production" that is intended to explain all data analytical and statistical steps used in the article. Furthermore we added the utilized R-Scripts [1, R Development Core Team] to allow recalculation and usage for own projects.

Contents

0 R-technical preparations 1

1 Raw data 2
1.1 Hyperpharingal gland sizes . . . 2
1.2 Brood development . . . 3
1.3 Larval Survival . . . 4

2 The actual questions 7
2.1 Question 1: Influence on the HPG size . . . 8
2.1.1 Exploratory data analysis (EDA) . . . 8
2.1.2 Inferential analysis: Effect of treatment in week 2 . . . 9
2.1.3 Posthoc tests in week 2 – comparisons with the Control . . . 10
2.1.4 Inferential analysis: Effect of treatment in week 7 . . . 12
2.1.5 Posthoc tests in week 7 – comparisons with the Control . . . 14
2.2 Question 2: Influence on the brood development . . . 15
2.2.1 Longitudinal EDA . . . 15
2.2.2 Treatment effect on number of eggs . . . 17
2.2.3 Posthoc tests for time trend of eggs – comparisons with the Control . . . 22
2.2.4 Treatment effect on number of larvae . . . 23
2.2.5 Posthoc tests for time trend of larvae – comparisons with the Control . . . 26
2.2.6 Treatment effect on number of capped brood cells . . . 27
2.2.7 Posthoc tests for time trend of capped brood cells – comparisons with the Control . . 30
2.3 Question 3: Impact on the larval survival . . . 31

2.3.1 Longitudinal EDA . . . 31
2.3.2 Treatment effect on larval survival in phase 1 . . . 33
2.3.3 Posthoc tests in survival phase 1 – comparisons with the Control . . . 37
2.3.4 Treatment effect on larval survival in phase 2 . . . 39
2.3.5 Posthoc tests in survival phase 2 – comparisons with the Control . . . 43
2.4 Question 4: Compensation rate . . . 45
2.4.1 Larvae-to-eggs ratio: Longitudinal EDA . . . 45
2.4.2 Treatment effect on larvae-to-eggs ratio (WITHOUT the extreme outlier) . . . 47
2.4.3 Posthoc tests for time trend of larvae-to-eggs ratio (WITHOUT the extreme outlier) – comparisons with the Control . . . 50
2.4.4 Treatment effect on larvae-to-eggs ratio (WITH the extreme outlier) . . . 51
2.4.5 Posthoc tests for time trend of larvae-to-eggs ratio (WITH the extreme outlier) – comparisons with the Control . . . 54
2.4.6 Capped-brood-to-larvae ratio . . . 55
2.4.7 Treatment effect on capped-brood-to-larvae ratio . . . 57
2.4.8 Posthoc tests for time trend of capped-brood-to-larvae ratio – comparisons with the Control . . . 60

3 Software & References **61**

List of Figures

1 Q1: HPG sizes by treatment for weeks 2 and 7 (boxplots) . . . 8
2 Q1: HPG sizes by treatment for weeks 2 and 7 (barplots) . . . 8
3 Q1: HPG by treatment in week 2: diagnostic plots . . . 11
4 Q1: HPG by treatment in week 7: diagnostic plots . . . 13
5 Q1: HPG sizes by treatment for weeks 2 and 7 (confidence intervals) . . . 14
6 Q2: Eggs along weeks by treatment . . . 15
7 Q2: Larvae along weeks by treatment . . . 15
8 Q2: Capped brood cells along weeks by treatment . . . 15
9 Q2: Eggs, larvae, brood development along weeks by treatment (summarized) . . . 16
10 Q2: Eggs along time by treatment: diagnostic plots . . . 20
11 Q2: Larvae along time by treatment: diagnostic plots . . . 21
12 Q2: Larvae along time by treatment: diagnostic plots . . . 25
13 Q2: Capped brood along time by treatment: diagnostic plots . . . 29
14 Q3: Proportions of surviving larvae along weeks . . . 31
15 Q3: Log-proportions of dead larvae along weeks . . . 32
16 Q3: Log-proportions of dead larvae in phase 1: diagnostic plots . . . 36
17 Q3: Log-proportions of dead larvae in phase 2: diagnostic plots . . . 42
18 Q4: Larvae-to-eggs ratio along weeks by treatment . . . 45
19 Q4: Mean larvae-to-eggs ratio along weeks by treatment . . . 46
20 Q4: Median larvae-to-eggs ratio along weeks by treatment . . . 46
21 Q4: Larvae-to-eggs ratio along time by treatment: diagnostic plots . . . 49
22 Q4: Larvae-to-eggs ratio along time by treatment: diagnostic plots . . . 53
23 Q4: Brood-to-larvae ratio along weeks by treatment . . . 55
24 Q4: Mean brood-to-larvae ratio along weeks by treatment . . . 56
25 Q4: Median brood-to-larvae ratio along weeks by treatment . . . 56
26 Q4: Brood-to-larvae ratio along time by treatment: diagnostic plots . . . 59

List of Tables

1 Q4: Mean and median larvae-to-eggs ratios per week . . . 45
2 Q4: Mean and median larvae-to-eggs ratios across weeks . . . 45
3 Q4: Mean and median capped-brood-to-larvae ratios per week . . . 55
4 Q4: Mean and median capped-brood-to-larvae ratios across weeks . . . 55

0 R-technical preparations

The current session:

```
R version 3.6.3 (2020-02-29)
Platform: x86_64-w64-mingw32/x64 (64-bit)
Running under: Windows 10 x64 (build 18363)

Matrix products: default

locale:
[1] LC_COLLATE=German_Germany.1252  LC_CTYPE=German_Germany.1252
[3] LC_MONETARY=German_Germany.1252 LC_NUMERIC=C
[5] LC_TIME=German_Germany.1252

attached base packages:
[1] parallel  stats     graphics  grDevices utils     datasets  methods   base

other attached packages:
 [1] RColorBrewer_1.1-2 pbkrtest_0.4-8.6   lme4_1.1-23        Matrix_1.2-18
 [5] multcomp_1.4-13    TH.data_1.0-10     MASS_7.3-51.5      mvtnorm_1.1-1
 [9] Hmisc_4.4-1        ggplot2_3.3.2      Formula_1.2-3      survival_3.1-8
[13] lattice_0.20-38

loaded via a namespace (and not attached):
 [1] statmod_1.4.34      zoo_1.8-8           tidyselect_1.1.0    xfun_0.16
 [5] purrr_0.3.4         splines_3.6.3       colorspace_1.4-1    vctrs_0.3.2
 [9] generics_0.0.2      htmltools_0.5.0     base64enc_0.1-3     rlang_0.4.7
[13] nloptr_1.2.2.2      pillar_1.4.6        foreign_0.8-75      glue_1.4.1
[17] withr_2.2.0         jpeg_0.1-8.1        lifecycle_0.2.0     stringr_1.4.0
[21] munsell_0.5.0       gtable_0.3.0        htmlwidgets_1.5.1   codetools_0.2-16
[25] latticeExtra_0.6-29 knitr_1.29          htmlTable_2.0.1     Rcpp_1.0.5
[29] backports_1.1.7     scales_1.1.1        checkmate_2.0.0     gridExtra_2.3
[33] png_0.1-7           digest_0.6.25       stringi_1.4.6       dplyr_1.0.2
[37] grid_3.6.3          tools_3.6.3         sandwich_2.5-1      magrittr_1.5
[41] tibble_3.0.3        cluster_2.1.0       crayon_1.3.4        pkgconfig_2.0.3
[45] ellipsis_0.3.1      data.table_1.13.0   minqa_1.2.4         rstudioapi_0.11
[49] R6_2.4.1            rpart_4.1-15        boot_1.3-24         nnet_7.3-12
[53] nlme_3.1-144        compiler_3.6.3
```

Self-made helper functions are "sourced":

```
> source("HelperFcts.R")
```

1 Raw data

The original raw data had been saved in several MS-Excel-sheets and exported as 'comma-separated values' (CSV) files, whose fields have been separated by semicolon (;). The decimal sign is the dot (.). The columns in the CSV-file possess names (in their first rows).

The file names are:

```
 [1] "BroodDevelopment_Brood.csv"      "BroodDevelopment_Brood_SUM.csv"
 [3] "BroodDevelopment_Eggs.csv"       "BroodDevelopment_Eggs_SUM.csv"
 [5] "BroodDevelopment_Larvae.csv"     "BroodDevelopment_Larvae_SUM.csv"
 [7] "HPGlandsizeS2.csv"               "HPGlandsizeS7.csv"
 [9] "LarvalSurvivalPhase1.csv"        "LarvalSurvivalPhase2.csv"
```

After passing an initial, minimal format check each of these 10 CSV-files is imported into a 'data frame' in R, Version 3.6.3 [1, R Development Core Team], and those data frames are combined into a list with components according to the file names.

```
> sapply(Filepaths, function(fn) unique(count.fields(fn, sep = ";")))
> Files <- lapply(Filepaths, read.csv, sep = ";")   # List of imported data frames
```

1.1 Hyperpharingal gland sizes

From the imported files we extract the data on honeybee hyperpharingal gland sizes and create two data frames named *"W2HPGland"* and *"W7HPGland"*. We check data consistency by controlling the first few rows of the created data frames as well as their summaries.

```
> Imp <- names(Files)[startsWith(names(Files), "HPG")]
> TMP <- lapply(Files[Imp], function(X) {
+   names(X) <- c("Treatment", "Hive", "Bee", "Size")
+   X <- within(X, {
+     Treatment <- factor(Treatment, levels = 1:4,
+                         labels = Trtmt, ordered = TRUE)
+     Hive <- factor(Hive, levels = 1:5, paste0("H", 1:5))
+     HiveID <- interaction(Treatment, Hive, lex.order = TRUE)
+     Bee <- factor(Bee, levels = 1:6, paste0("B", 1:6))
+     BeeID <- interaction(Treatment, Hive, Bee)
+     })
+   # print(str(X));   print(head(X));   print(tail(X));   print(summary(X))
+   # print(replications(~ Treatment * Hive * Bee, X))
+
+   droplevels(X)
+   })
> W2HPGland <- TMP[[1]]
> W7HPGland <- TMP[[2]]
> # str(W2HPGland);   str(W7HPGland)
```

```
> head(W2HPGland)

  Treatment Hive Bee     Size      BeeID  HiveID
1      Ctrl   H1  B1 174.8528 Ctrl.H1.B1 Ctrl.H1
2      Ctrl   H1  B2 180.0751 Ctrl.H1.B2 Ctrl.H1
3      Ctrl   H1  B3 173.6599 Ctrl.H1.B3 Ctrl.H1
4      Ctrl   H1  B4 164.3608 Ctrl.H1.B4 Ctrl.H1
5      Ctrl   H1  B5 170.2658 Ctrl.H1.B5 Ctrl.H1
6      Ctrl   H1  B6 202.5233 Ctrl.H1.B6 Ctrl.H1
```

```
> summary(W2HPGland)

    Treatment  Hive     Bee          Size                      BeeID          HiveID
 Ctrl    :30   H1:19   B1:17   Min.   : 64.06   Ctrl.H1.B1     : 1   Ctrl.H1  : 6
 1 ug/l  :16   H2:23   B2:17   1st Qu.:131.99   1 ug/l.H1.B1   : 1   Ctrl.H2  : 6
```

```
 10 ug/l :22   H3:17   B3:16   Median :156.46   10 ug/l.H1.B1 : 1   Ctrl.H3   : 6
 100 ug/l:23   H4:12   B4:15   Mean   :146.04   100 ug/l.H1.B1: 1   Ctrl.H4   : 6
               H5:20   B5:15   3rd Qu.:168.96   Ctrl.H2.B1    : 1   Ctrl.H5   : 6
                       B6:11   Max.   :202.52   1 ug/l.H2.B1  : 1   1 ug/l.H2: 6
                                                (Other)       :85   (Other)  :55
```

```
> head(W7HPGland)
```

```
  Treatment Hive Bee     Size      BeeID  HiveID
1      Ctrl   H1  B1 150.3844 Ctrl.H1.B1 Ctrl.H1
2      Ctrl   H1  B2 154.5890 Ctrl.H1.B2 Ctrl.H1
3      Ctrl   H1  B3 142.8989 Ctrl.H1.B3 Ctrl.H1
4      Ctrl   H1  B4 146.0462 Ctrl.H1.B4 Ctrl.H1
5      Ctrl   H1  B6 149.9465 Ctrl.H1.B6 Ctrl.H1
6      Ctrl   H2  B1 150.1412 Ctrl.H2.B1 Ctrl.H2
```

```
> summary(W7HPGland)
```

```
    Treatment  Hive    Bee          Size                    BeeID            HiveID
 Ctrl    :27   H1:21   B1:18   Min.   : 57.83   Ctrl.H1.B1    : 1   Ctrl.H4   : 6
 1 ug/l  :22   H2:16   B2:18   1st Qu.:117.57   1 ug/l.H1.B1  : 1   Ctrl.H5   : 6
 10 ug/l :22   H3:22   B3:18   Median :134.17   10 ug/l.H1.B1 : 1   1 ug/l.H1 : 6
 100 ug/l:29   H4:18   B4:18   Mean   :129.45   100 ug/l.H1.B1: 1   1 ug/l.H3 : 6
               H5:23   B5:17   3rd Qu.:148.21   Ctrl.H2.B1    : 1   10 ug/l.H4: 6
                       B6:11   Max.   :170.68   1 ug/l.H2.B1  : 1   10 ug/l.H5: 6
                                                (Other)       :94   (Other)   :64
```

1.2 Brood development

Here, we create the three data frames *Eggs*, *Brood* and *Larvae* and check data consistency as before.

```
> Imp <- names(Files)[endsWith(names(Files), "SUM")]
> for(nfi in Imp){
+   X <- Files[[nfi]]
+   RespName <- unlist(strsplit(nfi,"_"))[2]
+   names(X) <- c("Treatment", "Hive", "Week", RespName)
+   X <- within(X, {
+     Treatment <- factor(Treatment, levels = 1:4,
+                         labels = Trtmt, ordered = TRUE)
+     Hive <- factor(Hive, levels = 1:5, paste0("H", 1:5))
+     WeekFactor <- factor(Week, levels = 1:7, ordered = TRUE)
+     HiveID <- interaction(Treatment, Hive, lex.order = TRUE)
+     } )
+
+   X <- droplevels(X)   # droplevels() added 2017-21-22
+   # print(str(X));   print(head(X));   print(tail(X));   print(summary(X))
+   # print(replications(~ Treatment * Hive * WeekFactor, X))
+   assign(RespName, X)
+   }
```

```
> head(Eggs)
```

```
  Treatment Hive Week Eggs  HiveID WeekFactor
1      Ctrl   H1    1  180 Ctrl.H1          1
2      Ctrl   H2    1  267 Ctrl.H2          1
3      Ctrl   H3    1  242 Ctrl.H3          1
4      Ctrl   H4    1  268 Ctrl.H4          1
5      Ctrl   H1    2  246 Ctrl.H1          2
6      Ctrl   H2    2  238 Ctrl.H2          2
```

```
> summary(Eggs)
```

```
   Treatment  Hive        Week       Eggs             HiveID    WeekFactor
 Ctrl    :28  H1:21   Min.   :1   Min.   : 30.0   Ctrl.H1  : 7   1:16
 1 ug/l  :28  H2:21   1st Qu.:2   1st Qu.:150.0   Ctrl.H2  : 7   2:16
 10 ug/l :21  H3:28   Median :4   Median :188.5   Ctrl.H3  : 7   3:16
 100 ug/l:35  H4:21   Mean   :4   Mean   :204.1   Ctrl.H4  : 7   4:16
              H5:21   3rd Qu.:6   3rd Qu.:256.2   1 ug/l.H1: 7   5:16
                      Max.   :7   Max.   :386.0   1 ug/l.H2: 7   6:16
                                                  (Other)  :70   7:16
```

```
> head(Brood)
```

```
  Treatment Hive Week Brood  HiveID WeekFactor
1      Ctrl   H1    1   669 Ctrl.H1          1
2      Ctrl   H2    1   806 Ctrl.H2          1
3      Ctrl   H3    1   684 Ctrl.H3          1
4      Ctrl   H4    1   563 Ctrl.H4          1
5      Ctrl   H1    2   757 Ctrl.H1          2
6      Ctrl   H2    2   830 Ctrl.H2          2
```

```
> summary(Brood)
```

```
   Treatment  Hive        Week       Brood              HiveID    WeekFactor
 Ctrl    :28  H1:21   Min.   :1   Min.   :   0.0   Ctrl.H1  : 7   1:16
 1 ug/l  :28  H2:21   1st Qu.:2   1st Qu.: 467.0   Ctrl.H2  : 7   2:16
 10 ug/l :21  H3:28   Median :4   Median : 625.0   Ctrl.H3  : 7   3:16
 100 ug/l:35  H4:21   Mean   :4   Mean   : 597.9   Ctrl.H4  : 7   4:16
              H5:21   3rd Qu.:6   3rd Qu.: 770.5   1 ug/l.H1: 7   5:16
                      Max.   :7   Max.   :1408.0   1 ug/l.H2: 7   6:16
                                                   (Other)  :70   7:16
```

```
> head(Larvae)
```

```
  Treatment Hive Week Larvae  HiveID WeekFactor
1      Ctrl   H1    1    248 Ctrl.H1          1
2      Ctrl   H2    1    310 Ctrl.H2          1
3      Ctrl   H3    1    325 Ctrl.H3          1
4      Ctrl   H4    1    442 Ctrl.H4          1
5      Ctrl   H1    2    427 Ctrl.H1          2
6      Ctrl   H2    2    389 Ctrl.H2          2
```

```
> summary(Larvae)
```

```
   Treatment  Hive        Week       Larvae           HiveID    WeekFactor
 Ctrl    :28  H1:21   Min.   :1   Min.   :  1.0   Ctrl.H1  : 7   1:16
 1 ug/l  :28  H2:21   1st Qu.:2   1st Qu.:169.0   Ctrl.H2  : 7   2:16
 10 ug/l :21  H3:28   Median :4   Median :294.5   Ctrl.H3  : 7   3:16
 100 ug/l:35  H4:21   Mean   :4   Mean   :273.6   Ctrl.H4  : 7   4:16
              H5:21   3rd Qu.:6   3rd Qu.:364.5   1 ug/l.H1: 7   5:16
                      Max.   :7   Max.   :560.0   1 ug/l.H2: 7   6:16
                                                  (Other)  :70   7:16
```

1.3 Larval Survival

Here, we create the two data frames *Survival1* and *Survival2*, and again check data consistency by controlling the first few rows of the created data frames as well as their summaries.

```
> Imp <- names(Files)[startsWith(names(Files), "LarvalSurv")]
> TMP <- lapply(Files[Imp], function(X) {
+   names(X)[1:3] <- c("Treatment", "Hive", "LarvaeAtStart")
+   weeks <- as.numeric(substr(names(X)[-(1:3)], 2, 2))
+
```

```
+   X <- within(X, {
+     Treatment <- factor(Treatment, labels = Trtmt, ordered = TRUE)
+     Hive <- sapply(strsplit(as.vector(Hive), ".", fixed = TRUE), "[", 2)
+     Hive <- factor(Hive, levels = 1:5, paste0("H", 1:5))
+     HiveID <- interaction(Treatment, Hive, lex.order = TRUE)
+     })
+
+    # Compute proportions of larvae
+   wks <- paste0("S", weeks);    wksrel <- paste0(wks, ".rel")
+   X[wksrel] <- subset(X, select = wks) / X[[wks[1]]]
+   # print(str(X));   print(head(X));   print(tail(X));   print(summary(X))
+
+    # Reshape into "long" format
+   X <- reshape(X, drop = "LarvaeAtStart", varying = list(wks, wksrel),
+                v.names = c("Larvae", "Proportion.of.Larvae"),
+                timevar = "Week", times = weeks, direction = "long")
+
+    # Create Week factor and delete id column
+   X$WeekFactor <- factor(X$Week, levels = weeks, ordered = TRUE)
+   X$id <- row.names(X) <- NULL
+   droplevels(na.omit(X))
+   })
>  # "Transfer" and control
> Survival1 <- TMP[[1]]
> # str(Survival1);   head(Survival1);   tail(Survival1)
> # replications(~ Treatment * Hive * WeekFactor, Survival1)
>
> Survival2 <- TMP[[2]]
> # str(Survival2);   head(Survival2);   tail(Survival2)
> # replications(~ Treatment * Hive * WeekFactor, Survival2)

> head(Survival1)

  Treatment Hive    HiveID Week Larvae Proportion.of.Larvae WeekFactor
1      Ctrl   H1   Ctrl.H1    1     50                    1          1
2      Ctrl   H2   Ctrl.H2    1     61                    1          1
3      Ctrl   H3   Ctrl.H3    1     64                    1          1
4      Ctrl   H4   Ctrl.H4    1     55                    1          1
5    1 ug/l   H1 1 ug/l.H1    1     50                    1          1
6    1 ug/l   H2 1 ug/l.H2    1     78                    1          1

> summary(Survival1)

    Treatment  Hive         HiveID        Week          Larvae      Proportion.of.Larvae
 Ctrl    :16   H1:12   Ctrl.H1  : 4   Min.   :1.00   Min.   : 8.00   Min.   :0.1702
 1 ug/l  :16   H2:16   Ctrl.H2  : 4   1st Qu.:1.75   1st Qu.:32.50   1st Qu.:0.5428
 10 ug/l :16   H3:16   Ctrl.H3  : 4   Median :2.50   Median :41.50   Median :0.7358
 100 ug/l:20   H4:12   Ctrl.H4  : 4   Mean   :2.50   Mean   :43.54   Mean   :0.7163
               H5:12   1 ug/l.H1: 4   3rd Qu.:3.25   3rd Qu.:55.00   3rd Qu.:0.9538
                       1 ug/l.H2: 4   Max.   :4.00   Max.   :98.00   Max.   :1.0000
                       (Other)  :44
 WeekFactor
 1:17
 2:17
 3:17
 4:17

> head(Survival2)

  Treatment Hive    HiveID Week Larvae Proportion.of.Larvae WeekFactor
1      Ctrl   H1   Ctrl.H1    4     83                    1          4
```

```
2      Ctrl   H2   Ctrl.H2    4     61                     1          4
3      Ctrl   H3   Ctrl.H3    4     63                     1          4
4      Ctrl   H4   Ctrl.H4    4     53                     1          4
5    1 ug/l   H1 1 ug/l.H1    4     68                     1          4
6    1 ug/l   H2 1 ug/l.H2    4     58                     1          4
```

```
> summary(Survival2)
```

```
   Treatment  Hive         HiveID          Week           Larvae      Proportion.of.Larvae
 Ctrl    :16   H1:12   Ctrl.H1  : 4   Min.   :4.00   Min.   : 0.00   Min.   :0.0000
 1 ug/l  :16   H2:12   Ctrl.H2  : 4   1st Qu.:4.75   1st Qu.:14.00   1st Qu.:0.2667
 10 ug/l :16   H3:16   Ctrl.H3  : 4   Median :5.50   Median :35.00   Median :0.6250
 100 ug/l:16   H4:12   Ctrl.H4  : 4   Mean   :5.50   Mean   :33.50   Mean   :0.5765
               H5:12   1 ug/l.H1: 4   3rd Qu.:6.25   3rd Qu.:54.25   3rd Qu.:0.9508
                       1 ug/l.H2: 4   Max.   :7.00   Max.   :83.00   Max.   :1.0000
                       (Other)  :40
 WeekFactor
 4:16
 5:16
 6:16
 7:16
```

2 The actual questions

The actual questions, which are going to be examined in the following, are:

1. Does the Clothianidin concentration influence the Hypopharyngeal Gland (HPG) size? §2.1)
   (a) Does the treatment (Clothianidin concentration) significantly influence the Hypopharyngeal Gland (HPG) size in week 2? §2.1.2)
   (b) Does the treatment significantly influence the HPG size in week 7? §2.1.4)
   (c) Which treatments are significantly different to the control (0 ng/l Clothianidin)? (W2Gland and W7Gland have to be examined independent) §2.1.3) §2.1.5)
2. Does the Clothianidin concentration influence the bees development? §2.2)
   (a) Does the treatment (Clothianidin concentration) significantly influence number of eggs during the weeks? §2.2.2)
   (b) Does the treatment significantly influence number of larvae during the weeks? §2.2.4)?
   (c) Does the treatment significantly influence number of capped brood during the weeks? §2.2.6)?
   (d) If there is an treatment effect, which treatment differs significantly from the control §2.2.5 §2.2.7)?
3. Does the Clothianidin concentration have an impact on the larval survival? §2.3)
   (Exclude week four and week seven from data sets).
   (a) Does the treatment change the larval survival rate in phase 1 (week 1 to 3)? §2.3.2)
   (b) Which treatments differ significantly from the control in phase 1 (week 1 to 3)? §2.3.3)
   (c) Does the treatment change the larval survival rate in phase 2 (week 4 to 7)? §2.3.4)
   (d) Which treatments differ significantly from the control in phase 2 (week 4 to 7)? §2.3.5)
4. How much do the hives in the different treatment compensate for the clothianidin effect? §2.4)

2.1 Question 1: Influence on the HPG size

2.1.1 Exploratory data analysis (EDA)

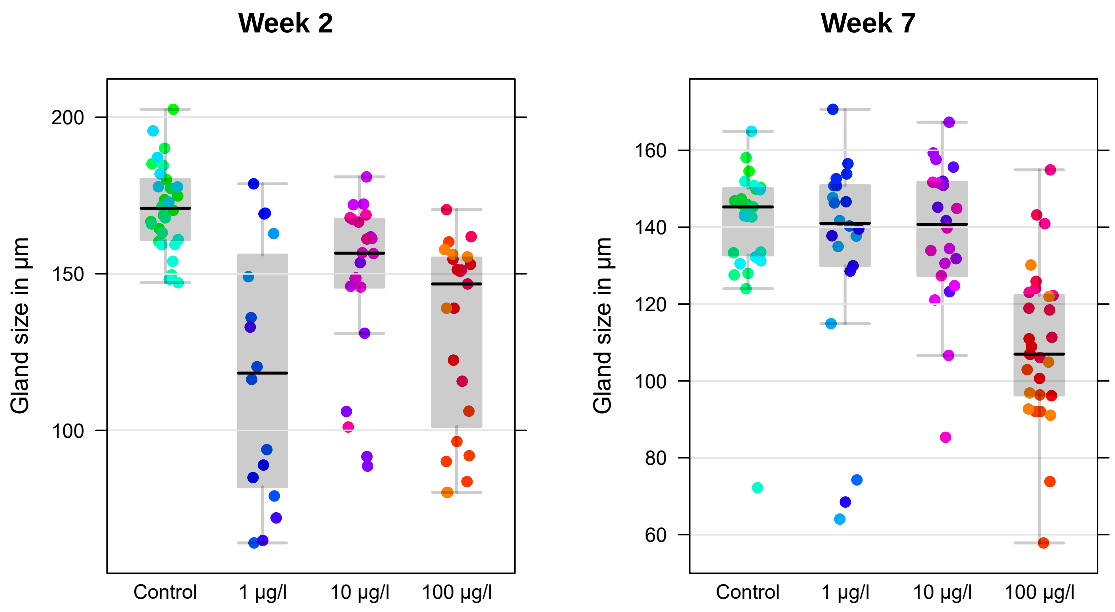

Figure 1: Per week: Boxplots and raw data of HPG size by treatment; week 2 on the left, week 7 on the right. Within each treatment different colors indicate origin from different hives. (File names: *Cloth-Q1_HPG_by_treatment_in_week2_Boxplots.pdf* and *Cloth-Q1_HPG_by_treatment_in_week7_Boxplots.pdf*)

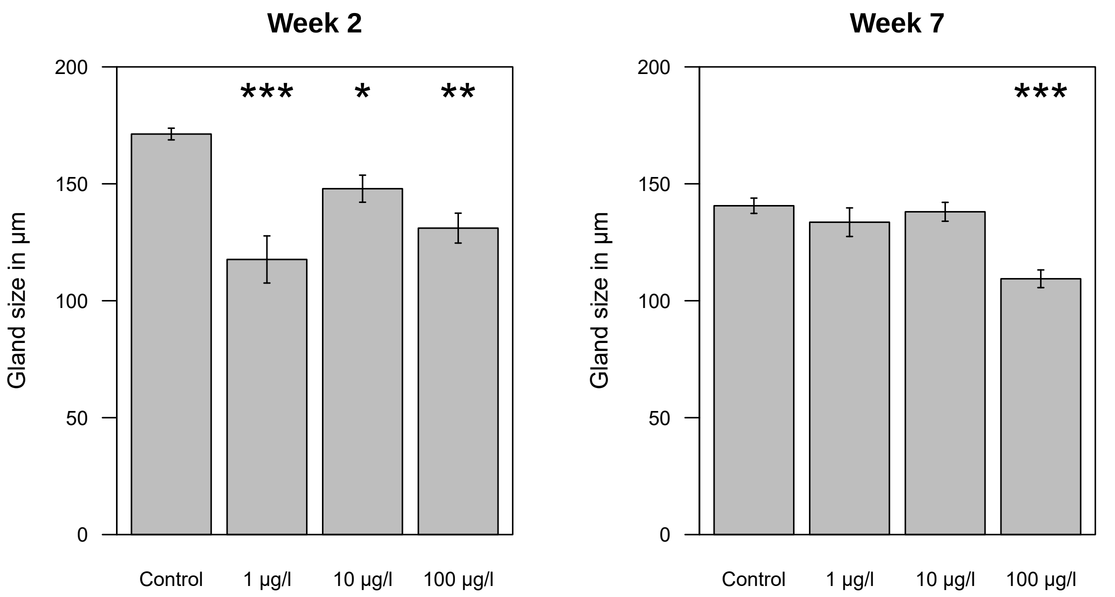

Figure 2: Per week: Barplots of average HPG size by treatment; week 2 on the left, week 7 on the right. Error bars indicate the standard error of the mean (ignoring the grouping structure by hives within each treatment; compare with 95 %-confidence intervals from a mixed-effects ANOVA in fig. 5). Asteriscs indicate significant differences in Dunnett contrasts with “Control” of mixed-effects ANOVAs, in anticipation of the analyses in §2.1.3 and §2.1.5. (File names: *Cloth-Q1_HPG_by_treatment_in_week2_Barplot.pdf* and *Cloth-Q1_HPG_by_treatment_in_week7_Barplot.pdf*)

2.1.2 Inferential analysis: Effect of treatment in week 2

Testing a hypothesis about fixed effects in a linear mixed model can always be based on a likelihood ratio or on a Wald test statistic which both have asymptotically (i.e., when the sample size goes to infinity) a χ^2-distribution under the null hypothesis. However, in cases with small and moderate sample sizes using a χ^2-distribution as an approximation of the (unknown true) distribution of the respective test statistic can be quite inappropriate and may lead to wrong conclusions.
For certain factorial model designs, it is alternatively possible to use a test statistic which has an F-distribution under the null hypothesis. However, such designs need to be balanced, e.g., with respect to the number of observations in the treatment groups.

In the present case, we unfortunately have neither large sample sizes nor a balanced design, so we have to resort to an alternative of the approximate χ^2-tests. In fact, we shall use *two* (to double-check the results) alternative, reliable methods for analysing fixed effects:

a) Kenward and Roger (KR) provide a modification of a Wald test statistic which has under the null hypothesis asymptotically an F-distribution (whose denominator degrees of freedom need and can be estimated) and is said to yield results more reliable than the F-test mentioned above. (This KR approach is for models fitted with *restricted* maximum likelihood (REML).)

b) A parametric bootstrap (PB) allows to determine the distribution (or moments thereof) of the likelihood ratio test statistic under the null hypothesis. (This is for models fitted with maximum likelihood (ML); so models fitted with REML need to be re-fitted with ML before.)

The R-package `pbkrtest` implements both the KR and the PB approach for tests regarding the fixed effects (in linear mixed models with independent errors). See [5] which describes the methods, their implementation and which contains also examples.

An even more applied description is given in §10.6 in [6, Faraway (2016)] which we shall follow here:

```
> fit1 <- lmer(Size ~ Treatment + (1|HiveID), data = W2HPGland,
+              contrasts = list(Treatment = "contr.treatment"))
> # print(summary(fit1), cor = FALSE)
> fit2 <- update(fit1, ~ . - Treatment) # Preparing analysis of a treatment effect.

>  # KR) Model comparison using an approximate F-test with degrees of freedom based
>  # on the Kenward-Roger approach:
>  #----------------------------------------------------------------------------
> (KR <- KRmodcomp(fit1, fit2))

F-test with Kenward-Roger approximation; time: 0.63 sec
large : Size ~ Treatment + (1 | HiveID)
small : Size ~ (1 | HiveID)
         stat     ndf     ddf F.scaling p.value
Ftest  5.4432  3.0000 13.0335   0.99983 0.01199 *
---
Signif. codes:  0 '***' 0.001 '**' 0.01 '*' 0.05 '.' 0.1 ' ' 1
```

```
>  # PB) Model comparison using the parametric bootstrap (after refitting with
>  # maximum likelihood automatically internally if required). (Computations are
>  # done with multiple processors in parallel by means of package parallel.)
>  #----------------------------------------------------------------------------
> cl <- makeCluster(rep("localhost", detectCores()))   # Create as many clusters as
>                                                      # there are cores.
> set.seed(201712) # Ensures reproducibility of simulation-based results.
>  # Relevant in the following parametric bootstrap model comparison: outputted
>  # p-value in row "PBtest" (others are only for comparison with further methods).
> (PB <- summary(PBmodcomp(fit1, fit2, nsim = nsim.PBmodcomp, cl = cl)))
```

```
Bootstrap test; time: 317.45 sec;samples: 5000; extremes: 88;
large : Size ~ Treatment + (1 | HiveID)
small : Size ~ (1 | HiveID)
            stat      df   ddf  p.value
LRT      13.1476  3.0000       0.004328 **
PBtest   13.1476               0.017796 *
Gamma    13.1476               0.017109 *
Bartlett 10.2044  3.0000       0.016906 *
F         4.3825  3.0000 2.698 0.142976
---
Signif. codes:  0 '***' 0.001 '**' 0.01 '*' 0.05 '.' 0.1 ' ' 1

> stopCluster(cl)   # Stop clusters.
```

Summary: Treatment has a significant effect on the HPG size with a p-value of 0.01199 from the Kenward-Rogers method, and a p-value of 0.0178 based on the parametric bootstrap.

Model diagnostics for the model in `fit1` are found on page 11.

2.1.3 Posthoc tests in week 2 – comparisons with the Control

We perform Dunnett's multiple comparisons for the **one-sided** null hypothesis that the Clothianidin treatments *do not yield smaller* HPG sizes than seen in the Control group.

```
         Simultaneous Tests for General Linear Hypotheses

Multiple Comparisons of Means: Dunnett Contrasts


Fit: lmer(formula = Size ~ Treatment + (1 | HiveID), data = W2HPGland,
    contrasts = list(Treatment = "contr.treatment"))

Linear Hypotheses:
                     Estimate Std. Error z value  Pr(<z)
1 ug/l - Ctrl >= 0     -51.52      13.83  -3.725 < 0.001 ***
10 ug/l - Ctrl >= 0    -24.27      13.34  -1.819 0.03447 *
100 ug/l - Ctrl >= 0   -39.82      13.30  -2.994 0.00269 **
---
Signif. codes:  0 '***' 0.001 '**' 0.01 '*' 0.05 '.' 0.1 ' ' 1
(Adjusted p values reported -- Westfall method)
```

Summary: In week 2 all three Clothianidin treatments show significantly *smaller* HPG sizes than the Control. (Compare left panel of fig. 1.)

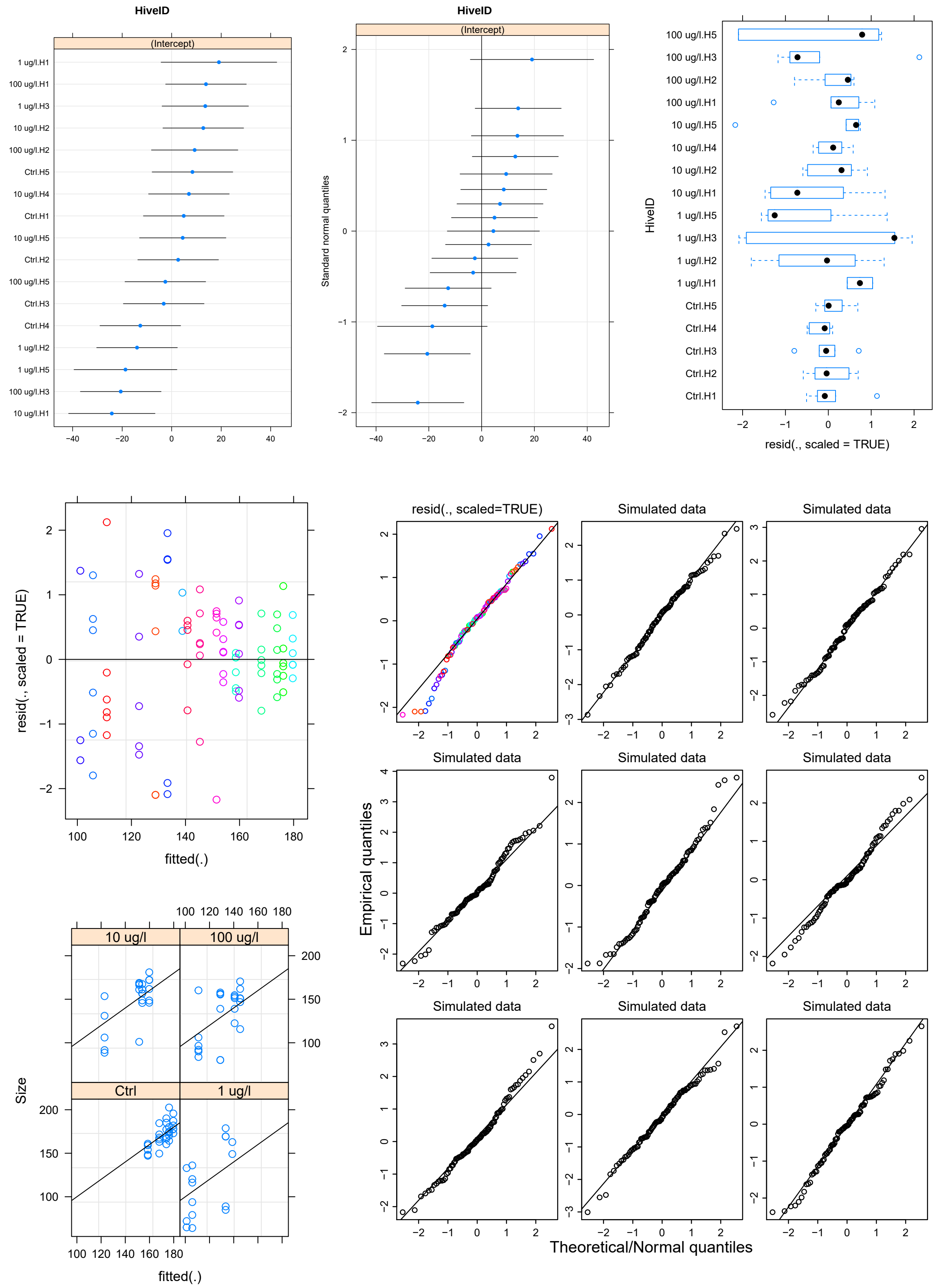

Figure 3: Diagnostic plots for the fitted linear mixed-effects model. (Color coding in plots of middle row identical to fig. 1.) Summarizing the findings (without explanation): the fitted model shows some indication against normality and homoscedasticity of the errors, but due to the not-too-small total sample size of 91 we consider the inferential results as reliable. (File names: *Cloth-Q1_Week2ModelDiagPlotX.pdf* with $X = 1, \ldots, 6$)

2.1.4 Inferential analysis: Effect of treatment in week 7

Analogous to §2.1.2:

```
> fit1 <- lmer(Size ~ Treatment + (1|HiveID), data = W7HPGland,
+              contrasts = list(Treatment = "contr.treatment"))
> # print(summary(fit1), cor = FALSE)
> fit2 <- update(fit1, ~ . - Treatment) # Preparing the analysis of a treatment effect.

>  # KR) Model comparison using an approximate F-test with degrees of freedom based
>  # on the Kenward-Roger approach:
>  #----------------------------------------------------------------------------
> (KR <- KRmodcomp(fit1, fit2))

F-test with Kenward-Roger approximation; time: 0.19 sec
large : Size ~ Treatment + (1 | HiveID)
small : Size ~ (1 | HiveID)
         stat     ndf     ddf F.scaling p.value
Ftest  5.0818  3.0000 13.9955         1 0.01379 *
---
Signif. codes:  0 '***' 0.001 '**' 0.01 '*' 0.05 '.' 0.1 ' ' 1
```

```
>  # PB) Model comparison using the parametric bootstrap (after refitting with
>  # maximum likelihood automatically internally if required). (Computations are
>  # done with multiple processors in parallel by means of package parallel.)
>  #----------------------------------------------------------------------------
> cl <- makeCluster(rep("localhost", detectCores()))   # Create as many clusters as
>                                                      # there are cores.
> set.seed(201712) # Ensures reproducibility of simulation-based results.
>  # Relevant in the following parametric bootstrap model comparison: outputted
>  # p-value in row "PBtest" (others are only for comparison with further methods).
> (PB <- summary(PBmodcomp(fit1, fit2, nsim = nsim.PBmodcomp, cl = cl)))

Bootstrap test; time: 257.16 sec;samples: 5000; extremes: 87;
large : Size ~ Treatment + (1 | HiveID)
small : Size ~ (1 | HiveID)
            stat      df    ddf p.value
LRT      12.7833  3.0000        0.00513 **
PBtest   12.7833                0.01760 *
Gamma    12.7833                0.01758 *
Bartlett 10.1380  3.0000        0.01743 *
F         4.2611  3.0000 2.7187 0.14623
---
Signif. codes:  0 '***' 0.001 '**' 0.01 '*' 0.05 '.' 0.1 ' ' 1

> stopCluster(cl)   # Stop clusters.
```

Summary: Treatment has a significant effect with a p-value of 0.01379 from the Kenward-Rogers method, and a p-value of 0.0176 based on the parametric bootstrap.

Model diagnostics for the model in `fit1`:

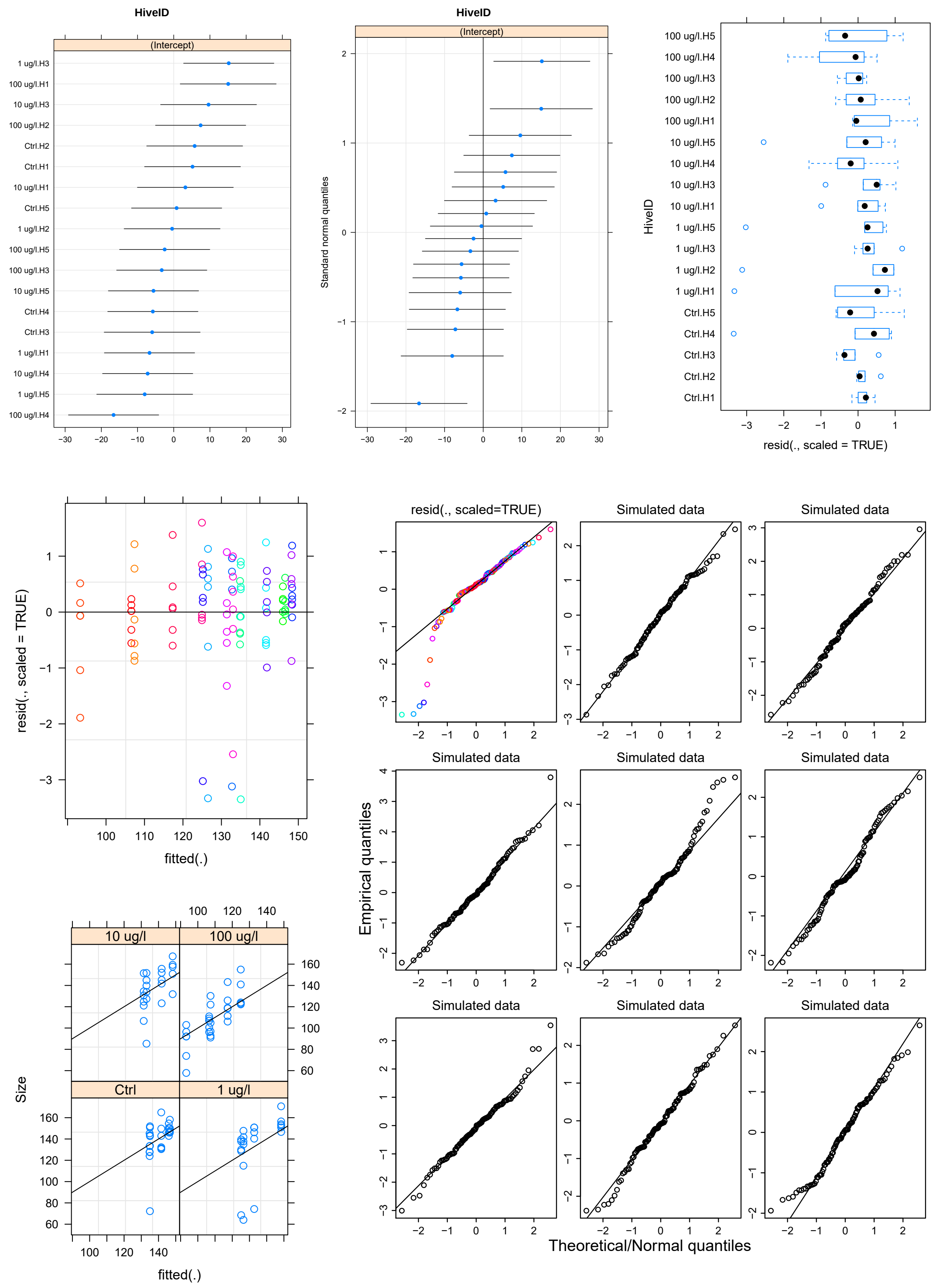

Figure 4: Diagnostic plots for the fitted linear mixed-effects model. (Color coding in plots of middle row identical to fig. 1.) Summarizing the findings (without explanation): the fitted model does show an indication against normality of the errors (outliers), but due to the not-too-small total sample size of 100 we consider the inferential results as acceptably reliable. (File names: *Cloth-Q1_Week7ModelDiagPlotX.pdf* with $X = 1, \ldots, 6$)

2.1.5 Posthoc tests in week 7 – comparisons with the Control

Analogous as in §2.1.3, we perform Dunnett's multiple comparisons for the **one-sided** null hypothesis that the Clothianidin treatments *do not yield smaller* HPG sizes than the Control.

```
         Simultaneous Tests for General Linear Hypotheses

Multiple Comparisons of Means: Dunnett Contrasts


Fit: lmer(formula = Size ~ Treatment + (1 | HiveID), data = W7HPGland,
    contrasts = list(Treatment = "contr.treatment"))

Linear Hypotheses:
                     Estimate Std. Error z value   Pr(<z)
1 ug/l - Ctrl >= 0     -7.603      9.380  -0.811 0.331134
10 ug/l - Ctrl >= 0    -2.190      9.380  -0.233 0.407710
100 ug/l - Ctrl >= 0  -30.899      8.806  -3.509 0.000699 ***
---
Signif. codes:  0 '***' 0.001 '**' 0.01 '*' 0.05 '.' 0.1 ' ' 1
(Adjusted p values reported -- Westfall method)
```

Summary: In week 7 only the 100 µg/l treatment shows significantly *smaller* HPG sizes than the Control.(Compare right panel of fig. 1.)

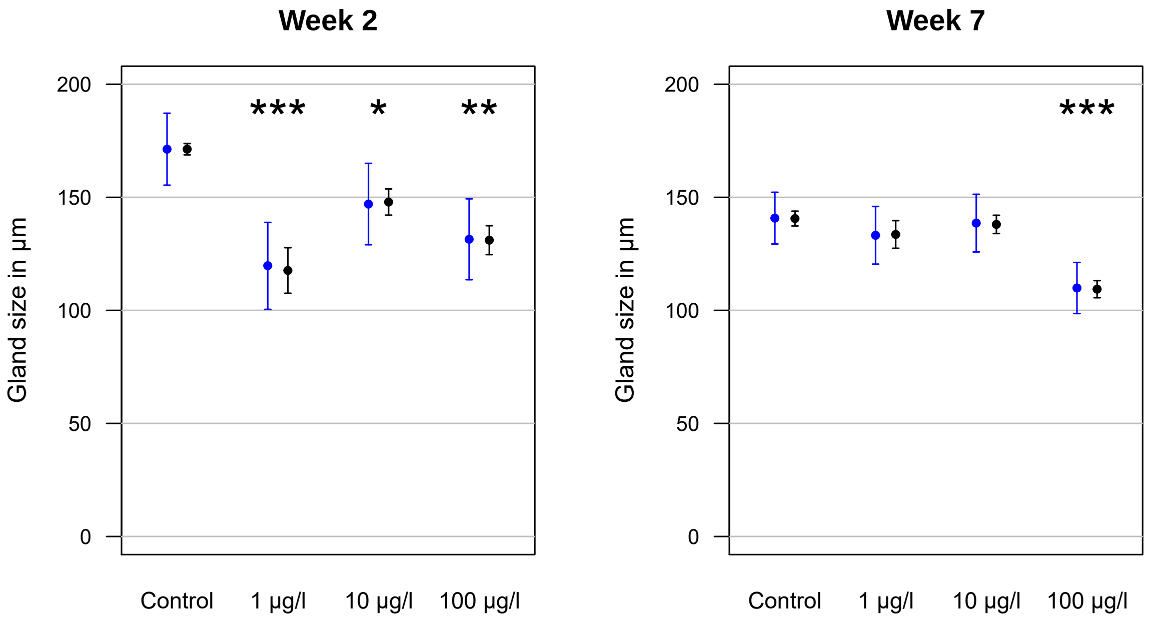

Figure 5: Per week and by treatment: Fixed-effects estimates (solid blue circles) with (non-simultaneous!) two-sided 95 %-confidence intervals (blue) of HPG size. (Week 2 on the left, week 7 on the right.) Black error bars indicate the mean HPG size plus/minus one standard error of the mean (ignoring the grouping structure by hives within each treatment). Note that the fixed-effects estimates differ slightly from the treatment specific "simple" mean HPG sizes. They are closer to the overal mean (why they are sometimes also called "shrinkage estimators"). Asteriscs indicate significant differences in one-sided Dunnett contrasts with "Control" of the mixed-effects ANOVA in §2.1.3 and §2.1.5, respectively. (File names: *Cloth-Q1_HPG_by_treatment_in_week2_CIplot.pdf* and *Cloth-Q1_HPG_by_treatment_in_week7_CIplot.pdf*)

2.2 Question 2: Influence on the brood development

2.2.1 Longitudinal EDA

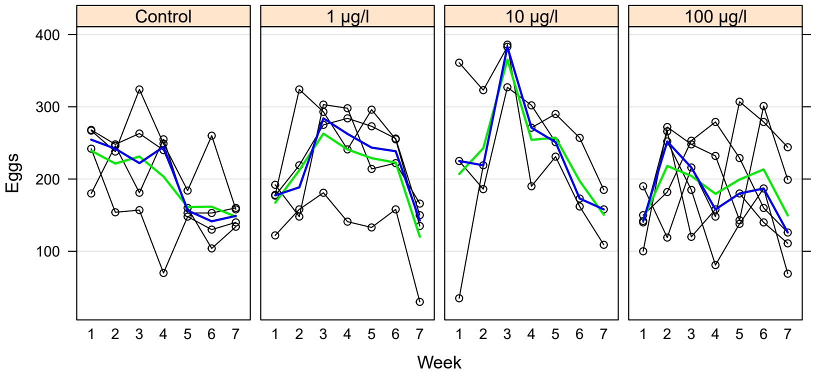

Figure 6: Numbers of eggs (open circles) in each hive along weeks by treatment, augmented by arithmetic means and medians across hives at each week. Values connected by a black polyline belong to the same hive; different polylines indicate different hives. The blue polylines connect the time specific median values, the green ones the respective means. (File name: *Cloth-Q2_Eggs_along_Weeks_by_Treat.pdf*)

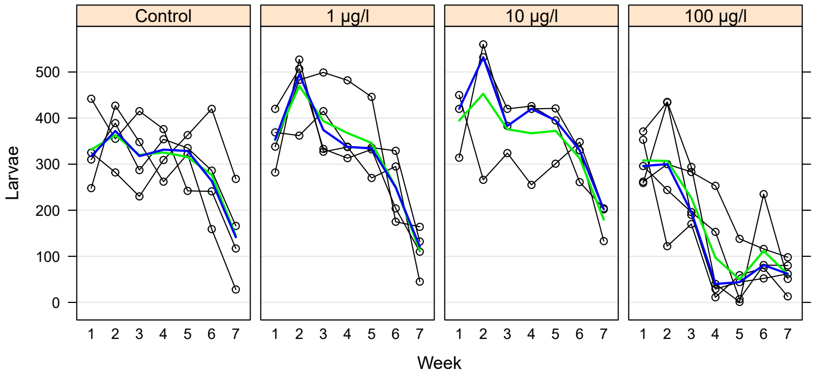

Figure 7: Larvae along weeks by treatment. Layout as explained in caption of fig. 6. (File name: *Cloth-Q2_Larvae_along_Weeks_by_Treat.pdf*)

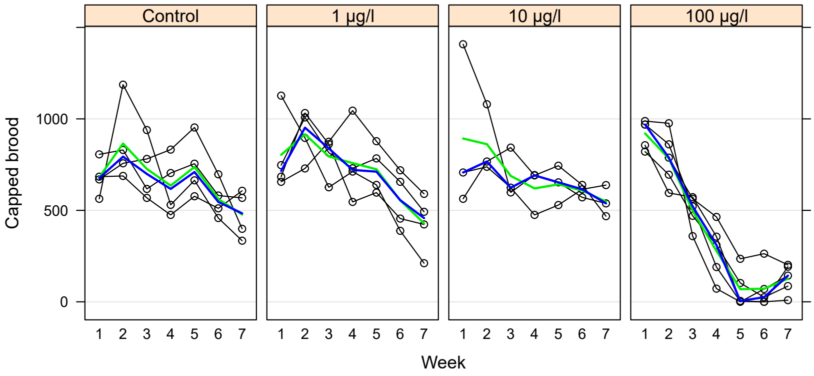

Figure 8: Capped brood cells along weeks by treatment: Layout as explained in caption of fig. 6. (File name: *Cloth-Q2_Eggs_along_Weeks_by_Treat.pdf*)

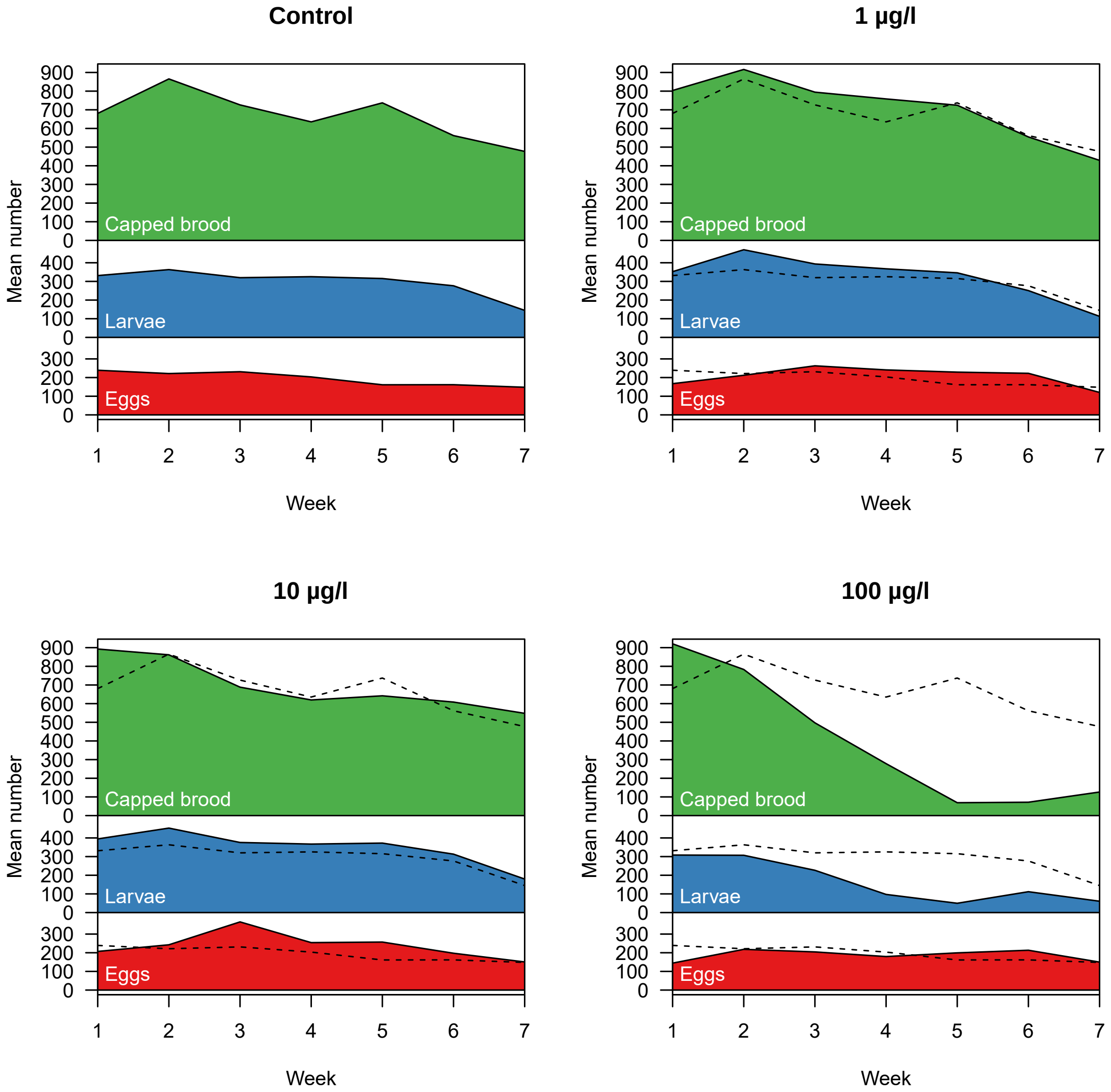

Figure 9: Summarizing and overlaying the information in fig. 6 - 8: Mean numbers of eggs, larvae, and capped brood cells along time by treatment: Values at each time point indicate mean values over all hives in the same treatment. The dashed lines in treatments different from “Control” indicate the respective time courses in the control group. (File name: *Cloth-Q2_BroodDevelopment.pdf*)

2.2.2 Treatment effect on number of eggs

We analyse the number of eggs by a linear mixed-effects model with a fixed effects linear or parabolic time trend along weeks, with fixed treatment main effects, and fixed interaction effects between weeks and treatment as well as between squared weeks and treatment. This is done for weeks centered at 3 – arbitrarily selected – so that the main effect of treatment represents the estimated number of eggs at week 3. Hives are modelled as random shift effects, thus accounting for the within-hive correlation.

For explanations regarding the testing methodology and for comments regarding the R-code see §2.1.2. We again follow, but also extend §10.6 in [6, Faraway (2016)].

```
> linfit1 <- lmer(Eggs ~ I(Week - 3) + (1 | HiveID), data = Eggs)

> linfit2 <- update(linfit1, ~ . + Treatment,
+                   contrasts = list(Treatment = "contr.treatment"))
> linfit3 <- update(linfit2, ~ . + I(Week - 3):Treatment)

> parfit3 <- update(linfit3, ~ . + I((Week - 3)^2) + I((Week - 3)^2):Treatment)
> print(summary(parfit3), cor = FALSE)

Linear mixed model fit by REML ['lmerMod']
Formula: Eggs ~ I(Week - 3) + (1 | HiveID) + Treatment + I((Week - 3)^2) +
    I(Week - 3):Treatment + Treatment:I((Week - 3)^2)
   Data: Eggs

REML criterion at convergence: 1150.3

Scaled residuals:
     Min       1Q   Median       3Q      Max
-2.65724 -0.52253  0.05715  0.48153  2.51319

Random effects:
 Groups   Name        Variance Std.Dev.
 HiveID   (Intercept) 1403     37.45
 Residual             2783     52.76
Number of obs: 112, groups:  HiveID, 16

Fixed effects:
                                  Estimate Std. Error t value
(Intercept)                       213.7321    23.4417   9.118
I(Week - 3)                       -15.2292     7.6151  -2.000
Treatment1 ug/l                    35.5000    33.1515   1.071
Treatment10 ug/l                   77.8155    35.8077   2.173
Treatment100 ug/l                 -10.3750    31.4503  -0.330
I((Week - 3)^2)                    -0.6577     2.8782  -0.229
I(Week - 3):Treatment1 ug/l        33.5982    10.7693   3.120
I(Week - 3):Treatment10 ug/l       28.2173    11.6322   2.426
I(Week - 3):Treatment100 ug/l      26.2101    10.2167   2.565
Treatment1 ug/l:I((Week - 3)^2)   -11.2768     4.0704  -2.770
Treatment10 ug/l:I((Week - 3)^2)  -12.4018     4.3966  -2.821
Treatment100 ug/l:I((Week - 3)^2)  -4.8042     3.8615  -1.244

>  # F-tests with approximated degrees of freedom according to Kenward-Roger:
>  #-----------------------------------------------------------------------
> (KR1 <- KRmodcomp(largeModel = linfit3, smallModel = linfit1))

F-test with Kenward-Roger approximation; time: 0.11 sec
large : Eggs ~ I(Week - 3) + (1 | HiveID) + Treatment + I(Week - 3):Treatment
small : Eggs ~ I(Week - 3) + (1 | HiveID)
        stat    ndf    ddf F.scaling p.value
Ftest  1.354  6.000 31.621   0.96696  0.2633
```

```
> (KR2 <- KRmodcomp(largeModel = parfit3, smallModel = linfit3))

F-test with Kenward-Roger approximation; time: 0.16 sec
large : Eggs ~ I(Week - 3) + (1 | HiveID) + Treatment + I((Week - 3)^2) +
    I(Week - 3):Treatment + Treatment:I((Week - 3)^2)
small : Eggs ~ I(Week - 3) + (1 | HiveID) + Treatment + I(Week - 3):Treatment
         stat     ndf     ddf F.scaling   p.value
Ftest  9.2969  4.0000 88.0000         1 2.587e-06 ***
---
Signif. codes:  0 '***' 0.001 '**' 0.01 '*' 0.05 '.' 0.1 ' ' 1

> (KR3 <- KRmodcomp(largeModel = parfit3, smallM = update(parfit3, ~ . - Treatment)))

F-test with Kenward-Roger approximation; time: 0.16 sec
large : Eggs ~ I(Week - 3) + (1 | HiveID) + Treatment + I((Week - 3)^2) +
    I(Week - 3):Treatment + Treatment:I((Week - 3)^2)
small : Eggs ~ I(Week - 3) + (1 | HiveID) + I((Week - 3)^2) + I(Week -
    3):Treatment + Treatment:I((Week - 3)^2)
         stat     ndf     ddf F.scaling p.value
Ftest  2.6086  3.0000 17.7679         1 0.08367 .
---
Signif. codes:  0 '***' 0.001 '**' 0.01 '*' 0.05 '.' 0.1 ' ' 1



>  # Parametric bootstrap:
>  #------------------------------------------------------------------------
> cl <- makeCluster(rep("localhost", detectCores()))
> set.seed(201712)   # For reproducibility.
>  # Relevant: p-value in row "PBtest"
> (PB1 <- summary(PBmodcomp(largeM = linfit3, smallM = linfit1, nsim = nsim.PBmodcomp,
+                            cl = cl)))

Bootstrap test; time: 240.24 sec;samples: 5000; extremes: 1406;
large : Eggs ~ I(Week - 3) + (1 | HiveID) + Treatment + I(Week - 3):Treatment
small : Eggs ~ I(Week - 3) + (1 | HiveID)
           stat     df    ddf p.value
LRT      8.6846 6.0000         0.1921
PBtest   8.6846                0.2813
Gamma    8.6846                0.2747
Bartlett 7.5258 6.0000         0.2749
F        1.4474 6.0000 2.3376  0.4420

> (PB2 <- summary(PBmodcomp(largeM = parfit3, smallM = linfit3, nsim = nsim.PBmodcomp,
+                            cl = cl)))

Bootstrap test; time: 357.50 sec;samples: 5000; extremes: 0;
large : Eggs ~ I(Week - 3) + (1 | HiveID) + Treatment + I((Week - 3)^2) +
    I(Week - 3):Treatment + Treatment:I((Week - 3)^2)
small : Eggs ~ I(Week - 3) + (1 | HiveID) + Treatment + I(Week - 3):Treatment
            stat      df    ddf   p.value
LRT      33.9631  4.0000        7.583e-07 ***
PBtest   33.9631                  0.00020 ***
Gamma    33.9631                1.462e-06 ***
Bartlett 31.4881  4.0000        2.434e-06 ***
F         8.4908  4.0000 2.6034   0.07087 .
---
Signif. codes:  0 '***' 0.001 '**' 0.01 '*' 0.05 '.' 0.1 ' ' 1

> (PB3 <- summary(PBmodcomp(largeM = parfit3, smallM = update(parfit3, ~ . - Treatment),
+                            nsim = nsim.PBmodcomp, cl = cl)))
```

```
Bootstrap test; time: 349.55 sec;samples: 5000; extremes: 477;
large : Eggs ~ I(Week - 3) + (1 | HiveID) + Treatment + I((Week - 3)^2) +
    I(Week - 3):Treatment + Treatment:I((Week - 3)^2)
small : Eggs ~ I(Week - 3) + (1 | HiveID) + I((Week - 3)^2) + I(Week -
    3):Treatment + Treatment:I((Week - 3)^2)
           stat     df    ddf p.value
LRT      8.0641 3.0000        0.04471 *
PBtest   8.0641               0.09558 .
Gamma    8.0641               0.09518 .
Bartlett 6.3215 3.0000        0.09697 .
F        2.6880 3.0000 2.7075 0.23406
---
Signif. codes:  0 '***' 0.001 '**' 0.01 '*' 0.05 '.' 0.1 ' ' 1
```

```
> stopCluster(cl)
```

Summary: Allowing (only) a linear time trend along weeks, including an interaction between time and treatment, there is no significant influence of treatment on the time trend of number of eggs (p-value = 0.2633 from the Kenward-Rogers method, and p-value = 0.2813 based on parametric bootstrap).

However, using a parabolic time trend along weeks, including an interaction between squared time and treatment (to account for the visible and diffferent curvatures of the time trends of numbers of eggs), there is a significant influence of treatment on the number of eggs (p-value = 2.587e-06 from the Kenward-Rogers method, and a p-value = 2e-04 based on parametric bootstrap).
This means in particular, that the time trends of the number of eggs are not the same in the four treatment groups.

On top, there is no significant treatment main effect in the parabolic model (p-value = 0.08367 from the Kenward-Rogers method, and p-value = 0.09558 based on parametric bootstrap). This means, the estimated average number of eggs at week 3 (!) are not significantly different between the four treatment groups.

Model diagnostics for the model in `linfit3`:

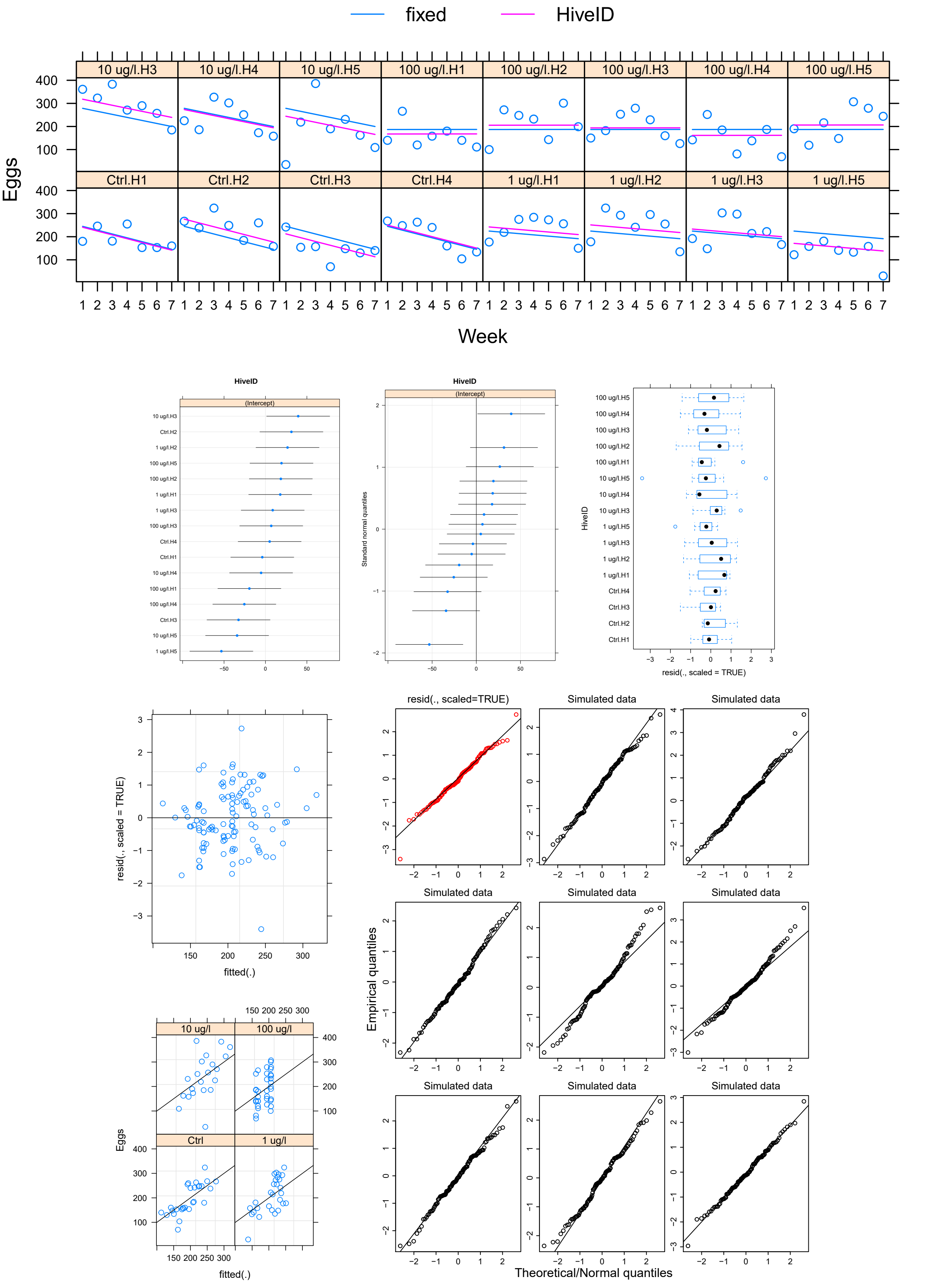

Figure 10: Diagnostic plots for the fitted linear mixed-effects model with *linear* time trends. Summarizing the findings (without explanation): the fitted model appears to fit well and does not show any serious indication against homoscedastic normality of the errors, so the inferential results are considered reliable. (File names: *Cloth-Q2_LinFit_EggsModelAugPred.pdf* and *Cloth-Q2_LinFit_EggsModelDiagX.pdf* with $X = 1, \ldots, 6$)

Model diagnostics for the model in `parfit3`:

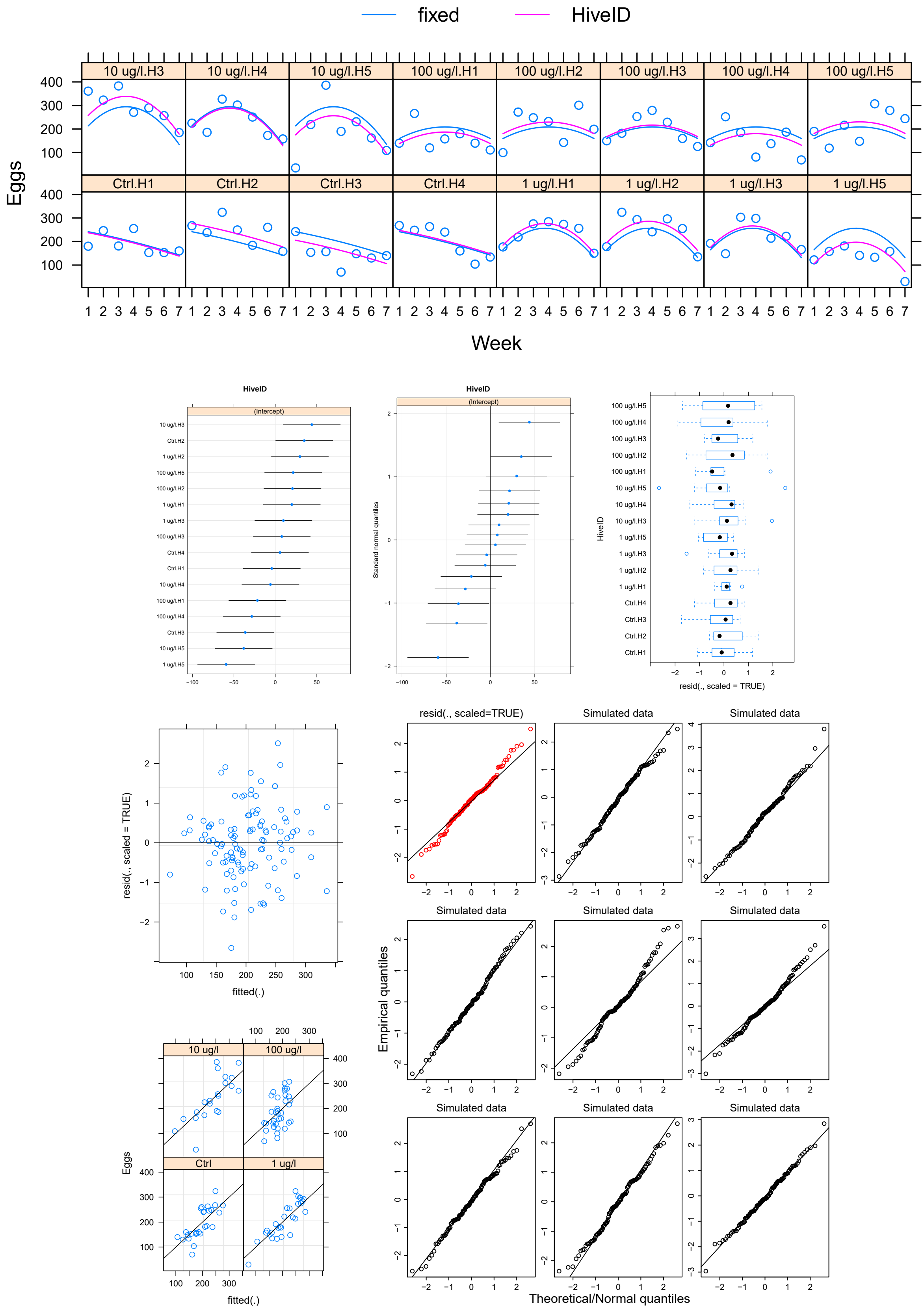

Figure 11: Diagnostic plots for the fitted linear mixed-effects model with *parabolic* time trends. Summarizing the findings (without explanation): the fitted model appears to fit quite well and does not show any indication against homoscedastic normality of the errors, so the inferential results are considered reliable. (File names: *Cloth-Q2_ParFit_EggsModelAugPred.pdf* and *Cloth-Q2_ParFit_EggsModelDiagX.pdf* with $X = 1, \dots, 6$)

2.2.3 Posthoc tests for time trend of eggs – comparisons with the Control

Here, we compare each Clothianidin treatment group to the control group with respect to the time trends in numbers of eggs.

Note: It is actually of little interest to analyse the main effects of treatment since they characterize the situation at only a single point in time, namely here at week 3. Instead, we focus on comparing the treatments with the control with respect to the (local) slope of their trend at week 3 and with respect to their (global) curvature. The first is represented by the interaction effect of treatment and week (centered at 3), and the latter by the by the interaction effect of treatment and squared week.

```
> fx <- fixef(parfit3)

> K <- diag(length(fx))[-(1:6),]
> rownames(K) <- names(fx)[-(1:6)]
> CompWCntrl <- glht(parfit3, linfct = K);     # summary(CompWCntrl)
> summary(CompWCntrl, test = adjusted(type = "Westfall"))

         Simultaneous Tests for General Linear Hypotheses

Fit: lmer(formula = Eggs ~ I(Week - 3) + (1 | HiveID) + Treatment +
    I((Week - 3)^2) + I(Week - 3):Treatment + Treatment:I((Week -
    3)^2), data = Eggs, contrasts = list(Treatment = "contr.treatment"))

Linear Hypotheses:
                                       Estimate Std. Error z value Pr(>|z|)
I(Week - 3):Treatment1 ug/l == 0         33.598     10.769   3.120  0.00926 **
I(Week - 3):Treatment10 ug/l == 0        28.217     11.632   2.426  0.02954 *
I(Week - 3):Treatment100 ug/l == 0       26.210     10.217   2.565  0.02656 *
Treatment1 ug/l:I((Week - 3)^2) == 0    -11.277      4.070  -2.770  0.02015 *
Treatment10 ug/l:I((Week - 3)^2) == 0   -12.402      4.397  -2.821  0.02015 *
Treatment100 ug/l:I((Week - 3)^2) == 0   -4.804      3.862  -1.244  0.21346
---
Signif. codes:  0 '***' 0.001 '**' 0.01 '*' 0.05 '.' 0.1 ' ' 1
(Adjusted p values reported -- Westfall method)
```

Summary: All Clothianidin treatments are significantly different from the control with respect to both their (local) slope at week 3 and their (global) curvature, with the exception of the 100 µg/l group: it does not differ significantly from the control in respect of its curvature.

2.2.4 Treatment effect on number of larvae

In proceeding analogously to §2.2.2 it turned out that the random effects of hives are in both the linear mixed-effects model with a linear time trend and the one with a parabolic time trend not significant. We tested this using exact restricted likelihood ratio tests implemented in package RLRsim. Analysis and results are not shown.

Hence, we decided to analyse the number of larvae by (purely) fixed-effects models with a linear or parabolic time trend along weeks, with treatment main effects, and interaction effects between weeks and treatment as well as between squared weeks and treatment. As in the previous paragraph this is done for weeks centered at 3 so that the main effect of treatment represents the estimated number of larvae at week 3.

```
> linfit1 <- lm(Larvae ~ I(Week - 3), data = Larvae)
> linfit2 <- update(linfit1, ~ . + Treatment,
+                   contrasts = list(Treatment = "contr.treatment"))
> linfit3 <- update(linfit2, ~ . + I(Week - 3):Treatment)
> parfit3 <- update(linfit3, ~ . + I((Week - 3)^2) + I((Week - 3)^2):Treatment)
> print(summary(parfit3), cor = FALSE)

Call:
lm(formula = Larvae ~ I(Week - 3) + Treatment + I((Week - 3)^2) +
    I(Week - 3):Treatment + Treatment:I((Week - 3)^2), data = Larvae,
    contrasts = list(Treatment = "contr.treatment"))

Residuals:
     Min       1Q   Median       3Q      Max
-150.976  -56.036   -0.614   44.882  176.036

Coefficients:
                                   Estimate Std. Error t value Pr(>|t|)
(Intercept)                        352.5357    20.9082  16.861  < 2e-16 ***
I(Week - 3)                         -6.6369    11.2917  -0.588 0.558013
Treatment1 ug/l                     66.6607    29.5686   2.254 0.026349 *
Treatment10 ug/l                    61.3214    31.9378   1.920 0.057704 .
Treatment100 ug/l                 -162.2500    28.0513  -5.784 8.37e-08 ***
I((Week - 3)^2)                     -9.8512     4.2679  -2.308 0.023046 *
I(Week - 3):Treatment1 ug/l         -3.8988    15.9689  -0.244 0.807615
I(Week - 3):Treatment10 ug/l        -6.4861    17.2484  -0.376 0.707682
I(Week - 3):Treatment100 ug/l      -55.0560    15.1494  -3.634 0.000442 ***
Treatment1 ug/l:I((Week - 3)^2)     -6.3810     6.0357  -1.057 0.292964
Treatment10 ug/l:I((Week - 3)^2)    -0.1528     6.5193  -0.023 0.981350
Treatment100 ug/l:I((Week - 3)^2)   17.3155     5.7259   3.024 0.003170 **
---
Signif. codes:  0 '***' 0.001 '**' 0.01 '*' 0.05 '.' 0.1 ' ' 1

Residual standard error: 78.23 on 100 degrees of freedom
Multiple R-squared:  0.7074,	Adjusted R-squared:  0.6753
F-statistic: 21.98 on 11 and 100 DF,  p-value: < 2.2e-16

>  # "Classical" ANOVA
>  #-----------------------------------------------------------------------
> (A1 <- anova(linfit1, linfit2, linfit3, parfit3))

Analysis of Variance Table

Model 1: Larvae ~ I(Week - 3)
Model 2: Larvae ~ I(Week - 3) + Treatment
Model 3: Larvae ~ I(Week - 3) + Treatment + I(Week - 3):Treatment
Model 4: Larvae ~ I(Week - 3) + Treatment + I((Week - 3)^2) + I(Week -
    3):Treatment + Treatment:I((Week - 3)^2)
  Res.Df     RSS Df Sum of Sq       F    Pr(>F)
```

```
1    110 1439492
2    107  812533  3    626959 34.1474 2.794e-15 ***
3    104  781770  3     30763  1.6755    0.1771
4    100  612012  4    169758  6.9344 5.728e-05 ***
---
Signif. codes:  0 '***' 0.001 '**' 0.01 '*' 0.05 '.' 0.1 ' ' 1
```

```
> (A2 <- anova(update(parfit3, ~ . - Treatment), parfit3))

Analysis of Variance Table

Model 1: Larvae ~ I(Week - 3) + I((Week - 3)^2) + I(Week - 3):Treatment +
    Treatment:I((Week - 3)^2)
Model 2: Larvae ~ I(Week - 3) + Treatment + I((Week - 3)^2) + I(Week -
    3):Treatment + Treatment:I((Week - 3)^2)
  Res.Df     RSS Df Sum of Sq      F   Pr(>F)
1    103 1145944
2    100  612012  3    533932 29.081 1.33e-13 ***
---
Signif. codes:  0 '***' 0.001 '**' 0.01 '*' 0.05 '.' 0.1 ' ' 1
```

Summary: Allowing (only) a linear time trend along weeks, including an interaction between time and treatment, there is no significant influence of treatment on the time trend of number of larvae (p-value = 0.1771).

Adding a parabolic time trend along weeks, including an interaction between squared time and treatment (to allow for a partly visible curvature of the time trends of numbers of larvae), is a significant contribution to the model (p-value = 5.728e-05).
This means in particular, that the parabolic time trends of the number of larvae are not the same in the four treatment groups.

On top, there is a significant treatment main effect in the parabolic model (p-value = 1.33e-13. This means, there is significant difference in the estimated average number of larvae at week 3 (!) between the four treatment groups.

Model diagnostics for the model in `parfit3`:

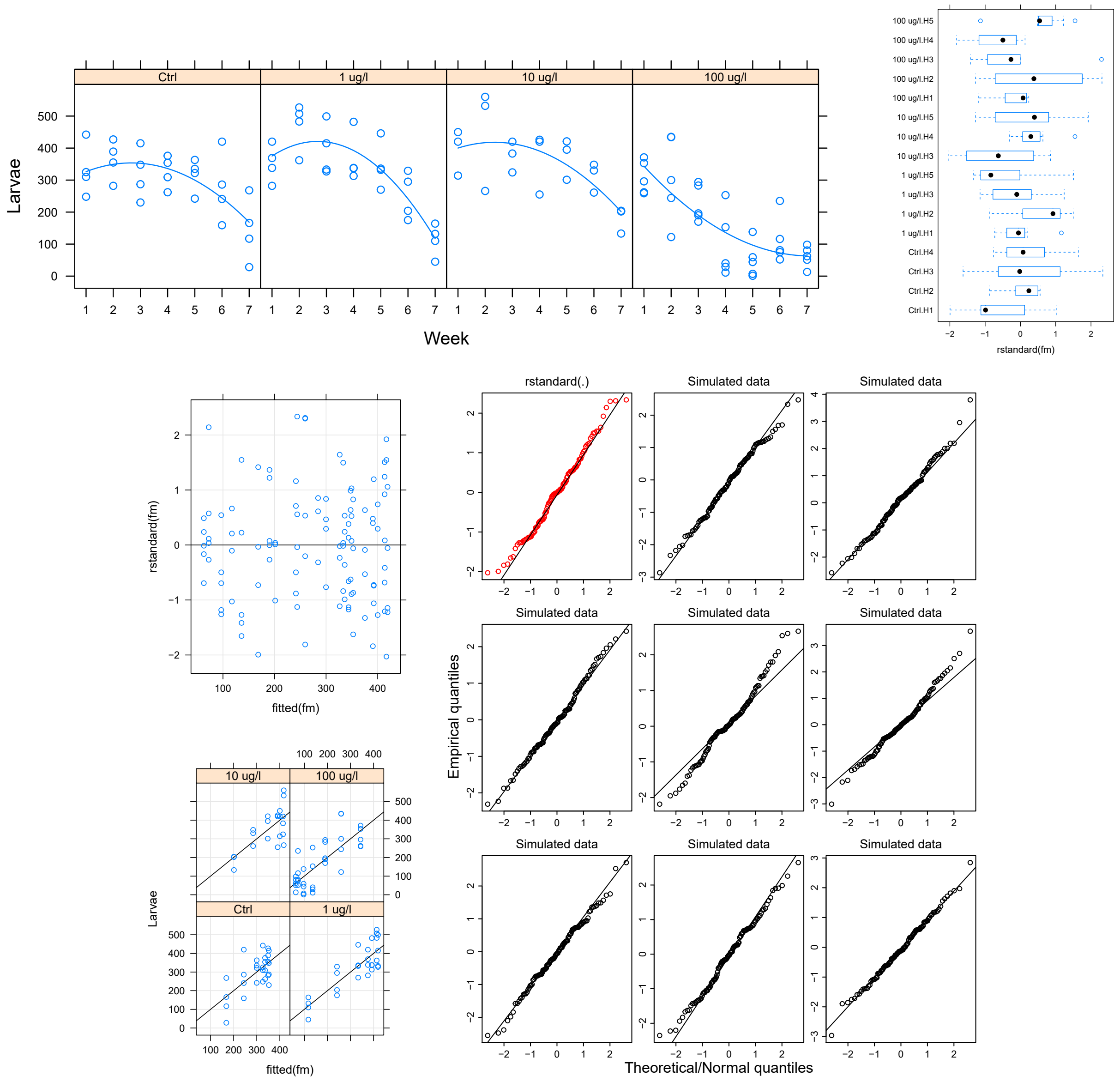

Figure 12: Diagnostic plots for the fitted linear fixed-effects model with *parabolic* time trends. Summarizing the findings (without explanation): the fitted model appears to fit very well and does not show any indication against homoscedastic normality of the errors, so the inferential results are considered reliable. (File names: *Cloth-Q2_ParFit_LarvaeModelAugPred.pdf* and *Cloth-Q2_ParFit_LarvaeModelDiagX.pdf* with $X = 3, \ldots, 6$)

2.2.5 Posthoc tests for time trend of larvae – comparisons with the Control

Here, we compare each Clothianidin treatment group to the control group with respect to the time trends in numbers of larvae.

Note: The same "Note" as in §2.2.3 applies!

```
> fx <- coef(parfit3)
> K <- diag(length(fx))[-(1:6),]
> rownames(K) <- names(fx)[-(1:6)]
> CompWCntrl <- glht(parfit3, linfct = K);     # summary(CompWCntrl)
> summary(CompWCntrl, test = adjusted(type = "Westfall"))

         Simultaneous Tests for General Linear Hypotheses

Fit: lm(formula = Larvae ~ I(Week - 3) + Treatment + I((Week - 3)^2) +
    I(Week - 3):Treatment + Treatment:I((Week - 3)^2), data = Larvae,
    contrasts = list(Treatment = "contr.treatment"))

Linear Hypotheses:
                                       Estimate Std. Error t value Pr(>|t|)
I(Week - 3):Treatment1 ug/l == 0        -3.8988    15.9689  -0.244  0.96041
I(Week - 3):Treatment10 ug/l == 0       -6.4861    17.2484  -0.376  0.95938
I(Week - 3):Treatment100 ug/l == 0     -55.0560    15.1494  -3.634  0.00226 **
Treatment1 ug/l:I((Week - 3)^2) == 0    -6.3810     6.0357  -1.057  0.63482
Treatment10 ug/l:I((Week - 3)^2) == 0   -0.1528     6.5193  -0.023  0.98135
Treatment100 ug/l:I((Week - 3)^2) == 0  17.3155     5.7259   3.024  0.01355 *
---
Signif. codes:  0 '***' 0.001 '**' 0.01 '*' 0.05 '.' 0.1 ' ' 1
(Adjusted p values reported -- Westfall method)
```

Summary: Only the 100 µg/l group is significantly different from the control with respect to both their (local) slope at week 3 and their (global) curvature.

2.2.6 Treatment effect on number of capped brood cells

In the analysis of capped brood cells we experienced the same phenomenon as with the number of larvae in §2.2.4: the random effects of hives are in both the linear mixed-effects model with a linear time trend and the one with a parabolic time trend not significant (using again exact restricted likelihood ratio tests implemented in package RLRsim; neither analysis nor results shown).

So, the number of caped brood cells is also analysed by (purely) fixed-effects models with a linear or parabolic time trend along weeks, with treatment main effects, and interaction effects between weeks and treatment as well as between squared weeks and treatment. As in the previous paragraph this is done for weeks centered at 3 so that the main effect of treatment represents the estimated number of larvae at week 3.

```
> linfit1 <- lm(Brood ~ I(Week - 3), data = Brood)
> linfit2 <- update(linfit1, ~ . + Treatment,
+                   contrasts = list(Treatment = "contr.treatment"))
> linfit3 <- update(linfit2, ~ . + I(Week - 3):Treatment)
> parfit3 <- update(linfit3, ~ . + I((Week - 3)^2) + I((Week - 3)^2):Treatment)
> print(summary(parfit3), cor = FALSE)

Call:
lm(formula = Brood ~ I(Week - 3) + Treatment + I((Week - 3)^2) +
    I(Week - 3):Treatment + Treatment:I((Week - 3)^2), data = Brood,
    contrasts = list(Treatment = "contr.treatment"))

Residuals:
    Min      1Q  Median      3Q     Max
-345.09  -98.42   -6.42   88.63  499.91

Coefficients:
                                  Estimate Std. Error t value Pr(>|t|)
(Intercept)                        753.036     40.606  18.545  < 2e-16 ***
I(Week - 3)                        -15.869     21.930  -0.724 0.470985
Treatment1 ug/l                     77.911     57.426   1.357 0.177926
Treatment10 ug/l                   -28.179     62.027  -0.454 0.650601
Treatment100 ug/l                 -296.050     54.479  -5.434 3.89e-07 ***
I((Week - 3)^2)                    -13.619      8.289  -1.643 0.103507
I(Week - 3):Treatment1 ug/l        -18.607     31.014  -0.600 0.549885
I(Week - 3):Treatment10 ug/l       -58.278     33.498  -1.740 0.084986 .
I(Week - 3):Treatment100 ug/l     -193.017     29.422  -6.560 2.40e-09 ***
Treatment1 ug/l:I((Week - 3)^2)     -3.375     11.722  -0.288 0.774004
Treatment10 ug/l:I((Week - 3)^2)    22.353     12.661   1.765 0.080535 .
Treatment100 ug/l:I((Week - 3)^2)   42.462     11.120   3.818 0.000233 ***
---
Signif. codes:  0 '***' 0.001 '**' 0.01 '*' 0.05 '.' 0.1 ' ' 1

Residual standard error: 151.9 on 100 degrees of freedom
Multiple R-squared:  0.7496,       Adjusted R-squared:  0.722
F-statistic: 27.21 on 11 and 100 DF,  p-value: < 2.2e-16

>  # "Classical" ANOVA
>  #-------------------------------------------------------------------------
> (A1 <- anova(linfit1, linfit2, linfit3, parfit3))

Analysis of Variance Table

Model 1: Brood ~ I(Week - 3)
Model 2: Brood ~ I(Week - 3) + Treatment
Model 3: Brood ~ I(Week - 3) + Treatment + I(Week - 3):Treatment
Model 4: Brood ~ I(Week - 3) + Treatment + I((Week - 3)^2) + I(Week -
    3):Treatment + Treatment:I((Week - 3)^2)
  Res.Df     RSS Df Sum of Sq      F    Pr(>F)
```

```
1    110 5922179
2    107 3744238  3   2177941 31.449 2.107e-14 ***
3    104 2836400  3    907838 13.109 2.749e-07 ***
4    100 2308417  4    527983  5.718 0.0003471 ***
---
Signif. codes:  0 '***' 0.001 '**' 0.01 '*' 0.05 '.' 0.1 ' ' 1
```

```
> (A2 <- anova(update(parfit3, ~ . - Treatment), parfit3))
```

```
Analysis of Variance Table

Model 1: Brood ~ I(Week - 3) + I((Week - 3)^2) + I(Week - 3):Treatment +
    Treatment:I((Week - 3)^2)
Model 2: Brood ~ I(Week - 3) + Treatment + I((Week - 3)^2) + I(Week -
    3):Treatment + Treatment:I((Week - 3)^2)
  Res.Df     RSS Df Sum of Sq      F    Pr(>F)
1    103 3592015
2    100 2308417  3   1283599 18.535 1.225e-09 ***
---
Signif. codes:  0 '***' 0.001 '**' 0.01 '*' 0.05 '.' 0.1 ' ' 1
```

Summary: Allowing (only) a linear time trend along weeks, including an interaction between time and treatment, there is a significant influence of treatment on the time trend of number of larvae (p-value = 2.749e-07).
This means in particular, that the linear time trends of the number of larvae are not the same in the four treatment groups.

Adding a parabolic time trend along weeks, including an interaction between squared time and treatment (to allow for a partly visible curvature of the time trends of numbers of larvae), is a significant contribution to the model (p-value = 0.0003471).
This means in particular, that the parabolic time trends of the number of larvae are not the same in the four treatment groups.

On top, there is a significant treatment main effect in the parabolic model (p-value = 1.225e-09. This means, there is significant difference in the estimated average number of larvae at week 3 (!) between the four treatment groups.

Model diagnostics for the model in `parfit3`:

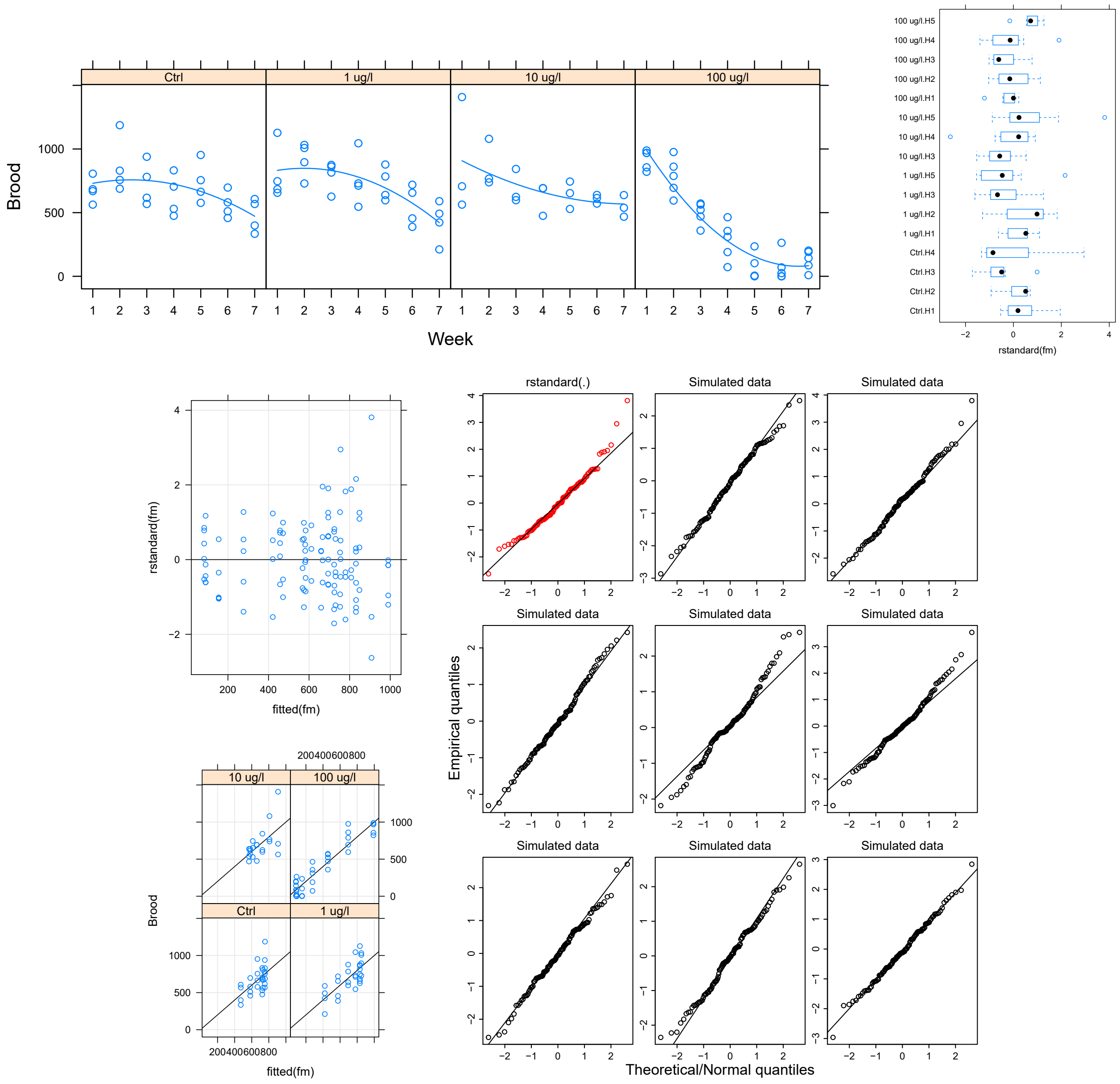

Figure 13: Diagnostic plots for the fitted linear fixed-effects model with *parabolic* time trends. Summarizing the findings (without explanation): the fitted model appears to fit well and does not show any serious indication against normality and homoscedasticity of the errors, so the inferential results can be considered reliable. (File names: *Cloth-Q2_ParFit_BroodModelAugPred.pdf* and *Cloth-Q2_ParFit_BroodModelDiagX.pdf* with $X = 3, \ldots, 6$)

2.2.7 Posthoc tests for time trend of capped brood cells – comparisons with the Control

Here, we compare each Clothianidin treatment group to the control group with respect to the time trends in numbers of capped brood cells.

Note: The same “Note” as in §2.2.3 applies!

```
> fx <- coef(parfit3)
> K <- diag(length(fx))[-(1:6),]
> rownames(K) <- names(fx)[-(1:6)]
> CompWCntrl <- glht(parfit3, linfct = K);     # summary(CompWCntrl)
> summary(CompWCntrl, test = adjusted(type = "Westfall"))

         Simultaneous Tests for General Linear Hypotheses

Fit: lm(formula = Brood ~ I(Week - 3) + Treatment + I((Week - 3)^2) +
    I(Week - 3):Treatment + Treatment:I((Week - 3)^2), data = Brood,
    contrasts = list(Treatment = "contr.treatment"))

Linear Hypotheses:
                                       Estimate Std. Error t value Pr(>|t|)
I(Week - 3):Treatment1 ug/l == 0        -18.607     31.014  -0.600  0.72745
I(Week - 3):Treatment10 ug/l == 0       -58.278     33.498  -1.740  0.22119
I(Week - 3):Treatment100 ug/l == 0     -193.017     29.422  -6.560  < 0.001 ***
Treatment1 ug/l:I((Week - 3)^2) == 0     -3.375     11.722  -0.288  0.77400
Treatment10 ug/l:I((Week - 3)^2) == 0    22.353     12.661   1.765  0.22119
Treatment100 ug/l:I((Week - 3)^2) == 0   42.462     11.120   3.818  0.00107 **
---
Signif. codes:  0 '***' 0.001 '**' 0.01 '*' 0.05 '.' 0.1 ' ' 1
(Adjusted p values reported -- Westfall method)
```

Summary: Only the 100 µg/l group is significantly different from the control with respect to both their (local) slope at week 3 and their (global) curvature.

2.3 Question 3: Impact on the larval survival

2.3.1 Longitudinal EDA

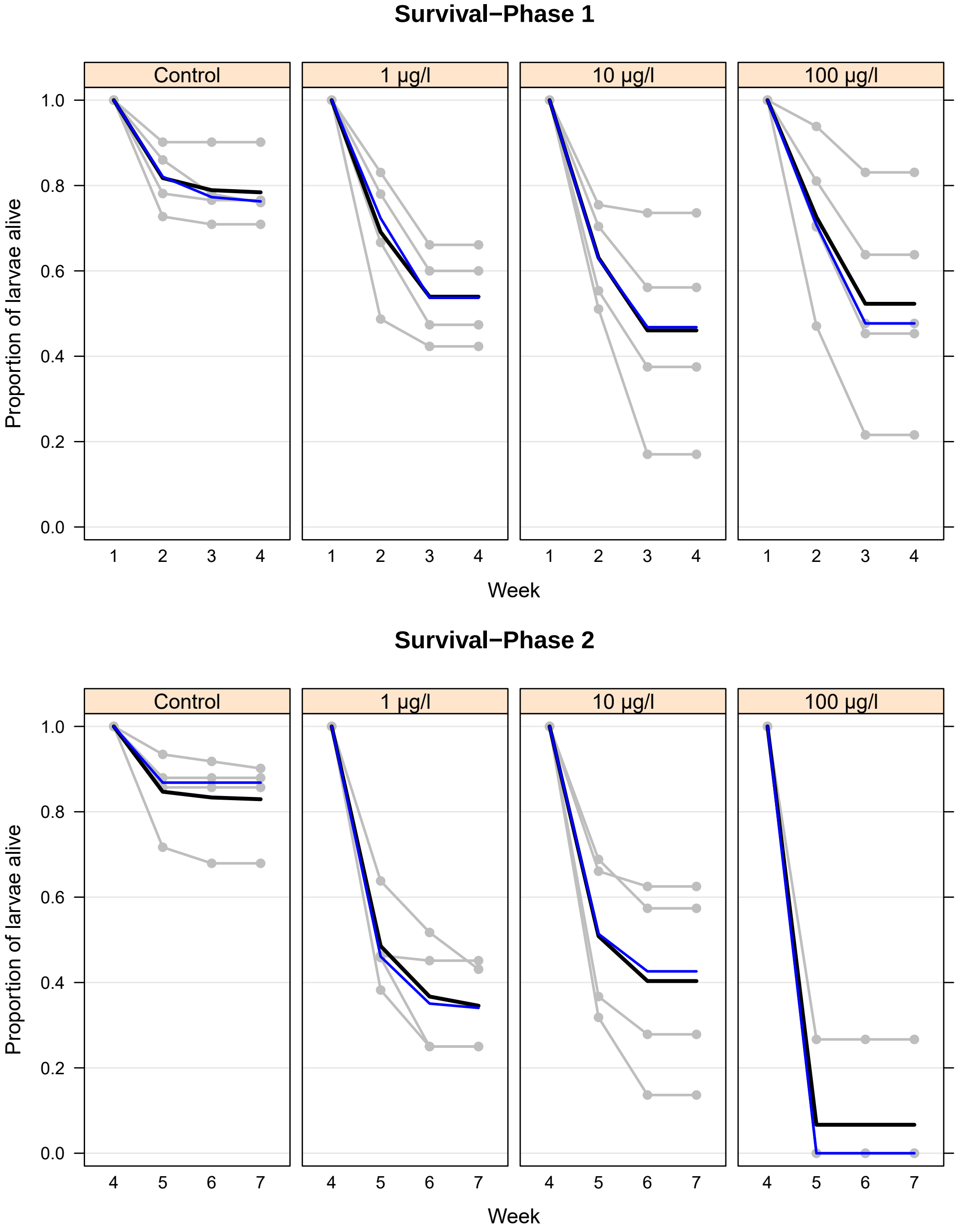

Figure 14: Proportions of surviving larvae (filled grey circles) in each hive along weeks by treatment in phase 1 (weeks 1 to 4, top) and in phase 2 (weeks 4 to 7, bottom). Proportions in each hive are calculated relative to hive value in first week of respective phase. The raw values are augmented by arithmetic means and medians across hives at each week. Values connected by a grey polyline belong to the same hive; different polylines indicate different hives. The blue polylines connect the time specific median values, the black ones the respective means. (File names: *Cloth-Q3_LarvalSurvivalPhase1.pdf* and *Cloth-Q3_LarvalSurvivalPhase2.pdf*)

Exploratory data analysis using q-q plots revealed that the raw data (displayed in fig. 14) are neither normally distributed nor homoscedastic. The variance of the data seems to increase with decreasing proportions of surviving larvae. To compensate that we take the logarithm of the proportions of *dead larvae* (= 1 -

proportion of *surviving larvae*) to obtain (approximately) normally distributed and homoscedastic values.

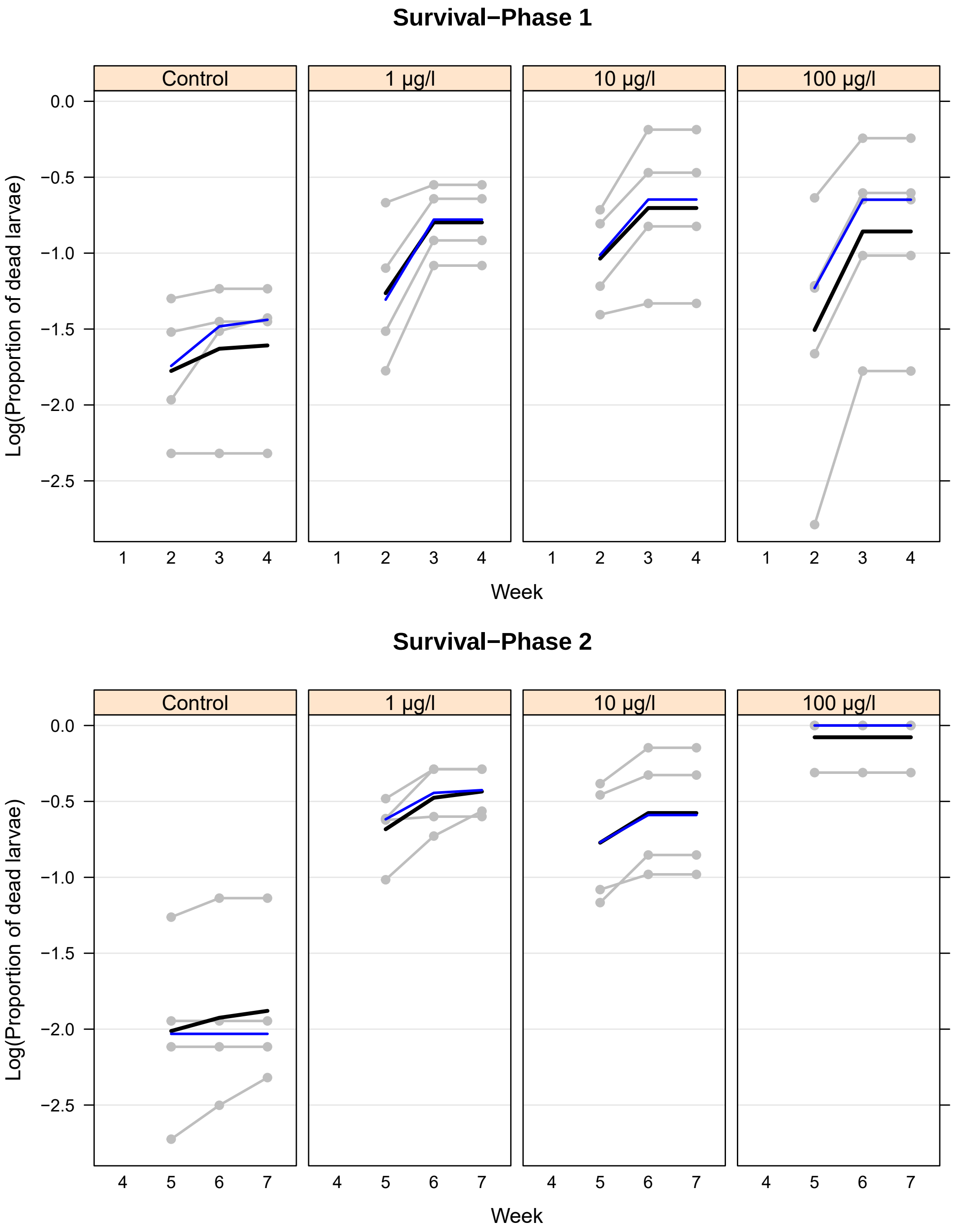

Figure 15: Logarithms of proportions of dead larvae in each hive along weeks by treatment: For layout details see caption of fig. 14. (Values at first week in each phase are missing since the logarithm of 0 (zero) is undefined.) (File names: *Cloth-Q3_LarvalLogSurvivalPhase1.pdf* and *Cloth-Q3_LarvalLogSurvivalPhase2.pdf*)

Note: Since in phase 1 the proportions of dead larvae virtually do not change from the penultimate to the last week in any but a single hive (see the Control group in top part of fig. 14), we exclude the last week of phase 1 from the following analysis. This is similar in phase 2 where proportions remain constant in the last two weeks in all but only two hives (one in the Control group and one in the 1 µg/l treatment; see bottom part of fig. 14). Since in addition, phase 2 has 100 % dead larvae from week five on in three of the four hives in the 100 µg/l treatment (see bottom part of fig. 14), we exclude this treatment group in phase 2 completely from the following analysis.

2.3.2 Treatment effect on larval survival in phase 1

We analyse the log-proportions of dead larvae using a two-factorial mixed-effects ANOVA with treatment and week as fixed-effects factors including interactions and with hive as random-effects grouping factor. (This model is the same as a two-factorial repeated measures ANOVA with treatment and week as fixed-effects factors and hive as random-effects factor for grouping the repeated measurements.)

We restrict the following analysis to weeks 2 and 3 of phase 1, and we proceed as in §2.1.2; for explanations and comments see also there.

```
> Data <- droplevels(subset(Survival1, subset = Week > 1 & Week < 4))

> fit0 <- lmer(log(1 - Proportion.of.Larvae) ~ (1 | HiveID), data = Data)
> fit1 <- update(fit0, ~ . + WeekFactor, contrasts = list(WeekFactor = "contr.treatment"))
> fit2 <- update(fit1, ~ . + Treatment, contrasts = list(WeekFactor = "contr.treatment",
+                                                        Treatment = "contr.treatment"))
> fit3 <- update(fit2, ~ . + WeekFactor:Treatment)
> print(summary(fit3), cor = FALSE)

Linear mixed model fit by REML ['lmerMod']
Formula:
log(1 - Proportion.of.Larvae) ~ (1 | HiveID) + WeekFactor + Treatment +
    WeekFactor:Treatment
   Data: Data

REML criterion at convergence: 28.7

Scaled residuals:
     Min       1Q   Median       3Q      Max
-1.48971 -0.41749  0.03685  0.35422  1.24429

Random effects:
 Groups   Name        Variance Std.Dev.
 HiveID   (Intercept) 0.25214  0.5021
 Residual             0.02421  0.1556
Number of obs: 34, groups:  HiveID, 17

Fixed effects:
                              Estimate Std. Error t value
(Intercept)                    -1.7761     0.2628  -6.757
WeekFactor3                     0.1464     0.1100   1.330
Treatment1 ug/l                 0.5122     0.3717   1.378
Treatment10 ug/l                0.7400     0.3717   1.991
Treatment100 ug/l               0.2699     0.3526   0.765
WeekFactor3:Treatment1 ug/l     0.3200     0.1556   2.056
WeekFactor3:Treatment10 ug/l    0.1868     0.1556   1.200
WeekFactor3:Treatment100 ug/l   0.5024     0.1476   3.404

>  # F-tests with approximated degrees of freedom according to Kenward-Roger:
>  #----------------------------------------------------------------------
> (KR0 <- KRmodcomp(largeModel = fit1, smallModel = fit0))

F-test with Kenward-Roger approximation; time: 0.13 sec
large : log(1 - Proportion.of.Larvae) ~ (1 | HiveID) + WeekFactor
small : log(1 - Proportion.of.Larvae) ~ (1 | HiveID)
        stat    ndf    ddf F.scaling   p.value
Ftest 37.826  1.000 16.000         1 1.396e-05 ***
---
Signif. codes:  0 '***' 0.001 '**' 0.01 '*' 0.05 '.' 0.1 ' ' 1

> (KR1 <- KRmodcomp(largeModel = fit2, smallModel = fit1))
```

```
F-test with Kenward-Roger approximation; time: 0.11 sec
large : log(1 - Proportion.of.Larvae) ~ (1 | HiveID) + WeekFactor + Treatment
small : log(1 - Proportion.of.Larvae) ~ (1 | HiveID) + WeekFactor
         stat     ndf     ddf F.scaling p.value
Ftest  1.9736  3.0000 13.0000         1  0.1678

> (KR2 <- KRmodcomp(largeModel = fit3, smallModel = fit2))

F-test with Kenward-Roger approximation; time: 0.11 sec
large : log(1 - Proportion.of.Larvae) ~ (1 | HiveID) + WeekFactor + Treatment +
    WeekFactor:Treatment
small : log(1 - Proportion.of.Larvae) ~ (1 | HiveID) + WeekFactor + Treatment
        stat    ndf    ddf F.scaling p.value
Ftest  4.125  3.000 13.000         1 0.02929 *
---
Signif. codes:  0 '***' 0.001 '**' 0.01 '*' 0.05 '.' 0.1 ' ' 1



>  # Parametric bootstrap:
>  #------------------------------------------------------------------------
> cl <- makeCluster(rep("localhost", detectCores()))
> set.seed(201712)   # For reproducibility.
>  # Relevant: p-value in row "PBtest"
> (PB0 <- summary(PBmodcomp(largeM = fit1, smallM = fit0, nsim = nsim.PBmodcomp,
+                          cl = cl)))

Bootstrap test; time: 377.22 sec;samples: 5000; extremes: 0;
large : log(1 - Proportion.of.Larvae) ~ (1 | HiveID) + WeekFactor
small : log(1 - Proportion.of.Larvae) ~ (1 | HiveID)
           stat     df    ddf   p.value
LRT      20.639  1.000        5.544e-06 ***
PBtest   20.639               0.0002000 ***
Gamma    20.639               1.201e-05 ***
Bartlett 18.636  1.000        1.582e-05 ***
F        20.639  1.000 20.603 0.0001851 ***
---
Signif. codes:  0 '***' 0.001 '**' 0.01 '*' 0.05 '.' 0.1 ' ' 1

> (PB1 <- summary(PBmodcomp(largeM = fit2, smallM = fit1, nsim = nsim.PBmodcomp,
+                          cl = cl)))

Bootstrap test; time: 426.21 sec;samples: 5000; extremes: 873;
large : log(1 - Proportion.of.Larvae) ~ (1 | HiveID) + WeekFactor + Treatment
small : log(1 - Proportion.of.Larvae) ~ (1 | HiveID) + WeekFactor
           stat     df    ddf p.value
LRT      6.1975 3.0000         0.1024
PBtest   6.1975                0.1748
Gamma    6.1975                0.1765
Bartlett 4.9243 3.0000         0.1774
F        2.0658 3.0000 2.7205  0.2966

> (PB2 <- summary(PBmodcomp(largeM = fit3, smallM = fit2, nsim = nsim.PBmodcomp,
+                          cl = cl)))

Bootstrap test; time: 528.22 sec;samples: 5000; extremes: 142;
large : log(1 - Proportion.of.Larvae) ~ (1 | HiveID) + WeekFactor + Treatment +
    WeekFactor:Treatment
small : log(1 - Proportion.of.Larvae) ~ (1 | HiveID) + WeekFactor + Treatment
            stat      df    ddf  p.value
LRT      11.5528  3.0000        0.009083 **
```

```
PBtest   11.5528               0.028594 *
Gamma    11.5528               0.026092 *
Bartlett  9.2566  3.0000       0.026066 *
F         3.8509  3.0000 2.7288 0.162266
---
Signif. codes:  0 '***' 0.001 '**' 0.01 '*' 0.05 '.' 0.1 ' ' 1
```

```
> stopCluster(cl)
```

Summary: There is a significant main effect of week (p-value = 1.396e-05 from Kenward-Rogers method, and p-value = 2e-04 based on parametric bootstrap), but there is a non-significant main effect of treatment (p-value = 0.1678 from Kenward-Rogers method, and p-value = 0.1748 based on parametric bootstrap).

Since there is a significant interaction effect between treatment and week (p-value = 0.02929 from Kenward-Rogers method, and p-value = 0.02859 based on parametric bootstrap), the main effect of each factor has to be interpreted as averaged across the levels of the other factor: The averages (across the four treatments) of log-proportions of dead larvae are significantly different between the two weeks, but the averaged log-proportions of dead larvae (across the two weeks) are not significantly different between the four treatment groups.
A bit more concrete: Interpreting the week main effect averaged across the levels of treatment implies that we compare the two points in time without separating the treatments. Even more precise: in each week we consider the population mean (of log-proportions of dead larvae) after "pooling" over the four treatment groups, i.e., the data from the four treatments are combined in each week as if there were only a single treatment group.
In turn, interpreting the treatment main effect averaged across the levels of week implies that we compare the four treatments without separating the weeks. Even more precise: in each treatment group we consider the population mean (of log-proportions of dead larvae) after "pooling" week 2 and week 3, i.e., the data from the two weeks are combined in each treatment group as if there were only a single point in time.

Additional remark: the significant interaction effect between treatment and week means that changes (!) in (average) log-proportions of dead larvae along weeks are different between treatments.

Model diagnostics for the model in `fit3`:

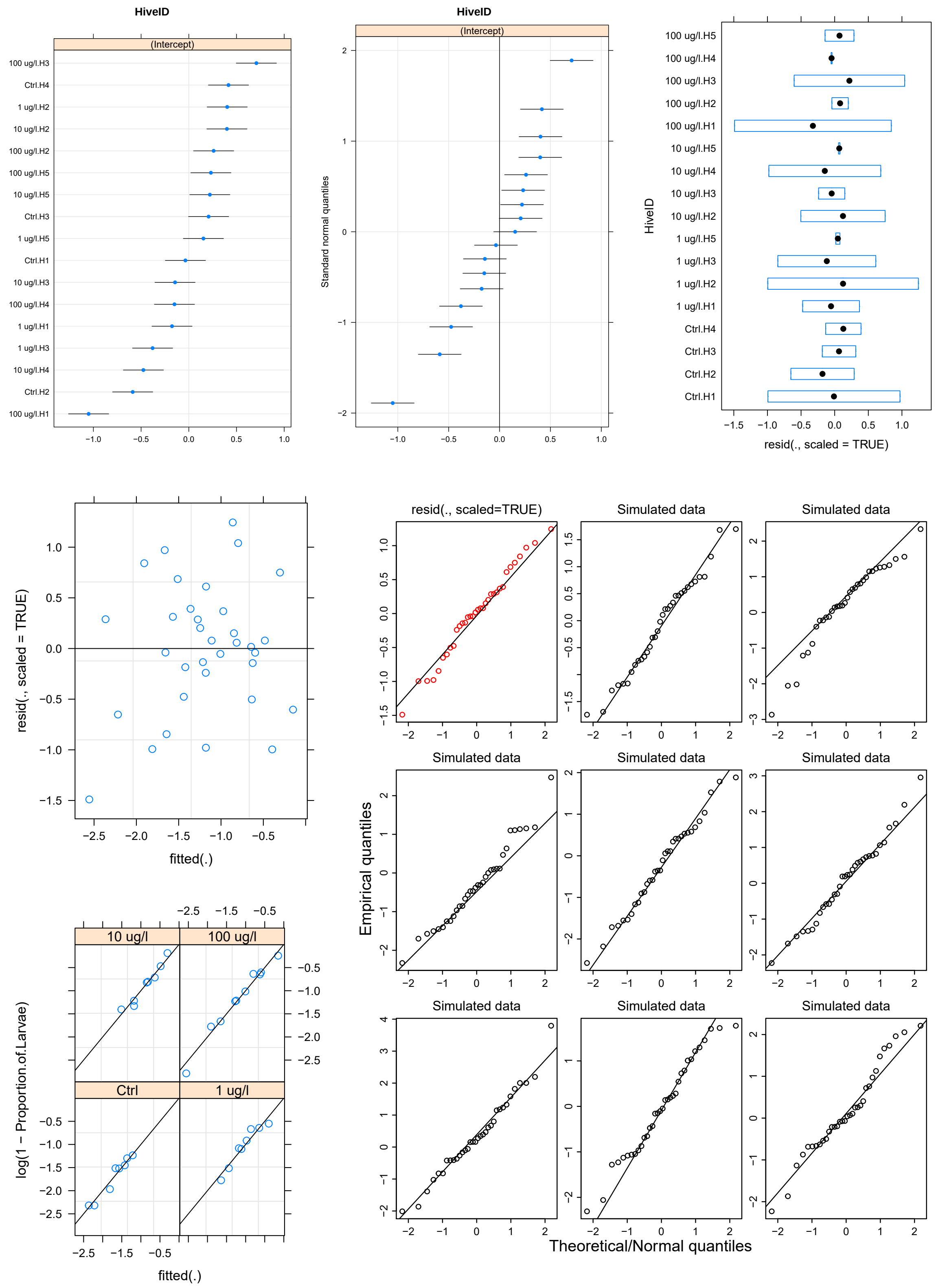

Figure 16: Diagnostic plots for the fitted linear mixed-effects model. Summarizing the findings (without explanation): the fitted model shows no indication against homoscedastic normality of the errors, so the inferential results are considered as reliable. (File names: *Cloth-Q3_Survival1ModelDiagPlotX.pdf* with $X = 1, \dots, 6$)

2.3.3 Posthoc tests in survival phase 1 – comparisons with the Control

We perform Dunnett's multiple comparisons for treatment (as an "inner" factor) within week (as "outer" factor) for the **one-sided** null hypothesis that the Clothianidin treatments *do not yield higher* values than the Control group. (The implementation is an adaption of the approach in chapter 3 of vignette "multcomp-examples" of the R-package multcomp.)

```
> fm <- fit3   # fm expects the 2-factorial mixed-effects ANOVA-object with interactions.
>  # Names of the 2 fixed-effects factors (Note: Order matters!):
> factornames <- list(Outer = "WeekFactor", Inner = "Treatment")
>  # Extract factor vector *data* which entered into fm via Data:
> InnerFactor <- Data[[factornames$Inner]]
> OuterFactor <- Data[[factornames$Outer]]

>  # 1. Dunnett's multiple comparisons for "inner" factor within "outer" factor:
>  #*************************************************************************
>   # Adapted from ch. 3 of multcomp-vignette "multcomp-examples". In particular the way
>   # of generating tmp for the model matrix X was modified to using levels() instead of
>   # unique(). (The latter would ignore a potentially non-alphanumerically sorted level
>   # order in the "inner factor" and could yield a wrong sign in contrast estimators.)
> tmp <- expand.grid(levels(InnerFactor), levels(OuterFactor))
> names(tmp) <- c(factornames$Inner, factornames$Outer)
> X <- model.matrix(formula(fm, fixed.only = TRUE)[-2], data = tmp)
> CM <- contrMat(table(InnerFactor), "Dunnett")   # Would also work with "Tukey".
> IM <- diag(nlevels(OuterFactor))
> dimnames(IM) <- list(levels(OuterFactor), levels(OuterFactor))
> Kron1 <- kronecker(IM, CM, make.dimnames = TRUE)

> fm.glht <- glht(fm, linfct = Kron1 %*% X, alternative = "greater")
> summary(fm.glht, test = adjusted(type = "Westfall"))

         Simultaneous Tests for General Linear Hypotheses

Fit: lmer(formula = log(1 - Proportion.of.Larvae) ~ (1 | HiveID) +
    WeekFactor + Treatment + WeekFactor:Treatment, data = Data,
    contrasts = list(WeekFactor = "contr.treatment", Treatment = "contr.treatment"))

Linear Hypotheses:
                        Estimate Std. Error z value Pr(>z)
2:1 ug/l - Ctrl <= 0      0.5122     0.3717   1.378 0.1414
2:10 ug/l - Ctrl <= 0     0.7400     0.3717   1.991 0.0580 .
2:100 ug/l - Ctrl <= 0    0.2699     0.3526   0.765 0.2220
3:1 ug/l - Ctrl <= 0      0.8322     0.3717   2.239 0.0414 *
3:10 ug/l - Ctrl <= 0     0.9268     0.3717   2.493 0.0241 *
3:100 ug/l - Ctrl <= 0    0.7723     0.3526   2.190 0.0418 *
---
Signif. codes:  0 '***' 0.001 '**' 0.01 '*' 0.05 '.' 0.1 ' ' 1
(Adjusted p values reported -- Westfall method)
```

Summary: In week 2 the log-proportions of dead larvae in hives treated with Clothianidin are not significantly *higher* than in the Control group, but in week 3 they are significantly *higher* in all three Clothianidin groups. (Compare top panel in fig. 15.)

Special consideration: Here, we select particular fixed effects coefficients of the fitted model for multiple comparisons with zero, namely coefficients number 6, 7, and 8 which means that we analyse only the interaction effects:

```
>  # 3. Selected fixed effects coefficients of fitted model are specified in
>  #    vector idx, e.g., for selected interaction effects:
>  #**********************************************************************
> idx <- c(6, 7, 8)   # Can also be just a scalar, i.e., a single level index.
```

```
> fixcoef <- fixef(fm)
> K <- matrix(0, nrow = length(fixcoef), ncol = length(fixcoef),
+             dimnames = list(names(fixcoef), names(fixcoef)))
> diag(K)[idx] <- 1
> K <- K[rowSums(abs(K)) > 0, ]

> fm.glht3 <- glht(fm, linfct = K, alternative = "greater")
> # summary(fm.glht3)
> summary(fm.glht3, test = adjusted(type = "Westfall"))

         Simultaneous Tests for General Linear Hypotheses

Fit: lmer(formula = log(1 - Proportion.of.Larvae) ~ (1 | HiveID) +
    WeekFactor + Treatment + WeekFactor:Treatment, data = Data,
    contrasts = list(WeekFactor = "contr.treatment", Treatment = "contr.treatment"))

Linear Hypotheses:
                                   Estimate Std. Error z value Pr(>z)
WeekFactor3:Treatment1 ug/l <= 0     0.3200     0.1556   2.056 0.0364 *
WeekFactor3:Treatment10 ug/l <= 0    0.1868     0.1556   1.200 0.1150
WeekFactor3:Treatment100 ug/l <= 0   0.5024     0.1476   3.404 <0.001 ***
---
Signif. codes:  0 '***' 0.001 '**' 0.01 '*' 0.05 '.' 0.1 ' ' 1
(Adjusted p values reported -- Westfall method)
```

Summary: The changes (!) (here: increases) from week 2 to week 3 in log-proportions of dead larvae are significantly *higher* in the 1 µg/l and the 100 µg/l Clothianidin group than in the Control group. The change (!) (here increase) from week 2 to week 3 in log-proportions of dead larvae in the 10 µg/l Clothianidin group is not significantly *higher* than in the Control group. (This does *not* proof that the change in the 10 µg/l treated hives is *not* different from the one in the control group, but the sample size is presumably to low to detect a significant difference.) (Compare top panel in fig. 15.)

Another special consideration: We carefully construct a combination of the one-sided tests from above:

```
> summary(glht(fm, linfct = rbind(Kron1 %*% X, K), alternative = "greater"),
+         test = adjusted(type = "Westfall"))

         Simultaneous Tests for General Linear Hypotheses

Fit: lmer(formula = log(1 - Proportion.of.Larvae) ~ (1 | HiveID) +
    WeekFactor + Treatment + WeekFactor:Treatment, data = Data,
    contrasts = list(WeekFactor = "contr.treatment", Treatment = "contr.treatment"))

Linear Hypotheses:
                                   Estimate Std. Error z value  Pr(>z)
2:1 ug/l - Ctrl <= 0                 0.5122     0.3717   1.378 0.21732
2:10 ug/l - Ctrl <= 0                0.7400     0.3717   1.991 0.05810 .
2:100 ug/l - Ctrl <= 0               0.2699     0.3526   0.765 0.22205
3:1 ug/l - Ctrl <= 0                 0.8322     0.3717   2.239 0.05056 .
3:10 ug/l - Ctrl <= 0                0.9268     0.3717   2.493 0.03359 *
3:100 ug/l - Ctrl <= 0               0.7723     0.3526   2.190 0.05056 .
WeekFactor3:Treatment1 ug/l <= 0     0.3200     0.1556   2.056 0.05584 .
WeekFactor3:Treatment10 ug/l <= 0    0.1868     0.1556   1.200 0.22067
WeekFactor3:Treatment100 ug/l <= 0   0.5024     0.1476   3.404 0.00251 **
---
Signif. codes:  0 '***' 0.001 '**' 0.01 '*' 0.05 '.' 0.1 ' ' 1
(Adjusted p values reported -- Westfall method)
```

Summary: If we combine the two test families from above into one family all statements from above hold still true on a significance level slightly higher than 5 %: a level of 6 % would yield the same conclusions.

2.3.4 Treatment effect on larval survival in phase 2

Recall the note on p. 32: Proportions of dead larvae remain constant in the last two weeks in almost all hives and the 100 µg/l treatment group has 100 % dead larvae from week five on in three of the four hives (see bottom part of fig. 14). Therefore, we exclude the 100 µg/l treatment group completely from the following analysis and restrict the analysis to weeks 5 and 6 of phase 2. We proceed as in §2.3.2.

```
> Data <- droplevels(subset(Survival2, subset = Week > 4 & Week < 7))

> fit0 <- lmer(log(1 - Proportion.of.Larvae) ~ (1 | HiveID), data = Data)
> fit1 <- update(fit0, ~ . + WeekFactor, contrasts = list(WeekFactor = "contr.treatment"))
> fit2 <- update(fit1, ~ . + Treatment, contrasts = list(WeekFactor = "contr.treatment",
+                                                        Treatment = "contr.treatment"))
> fit3 <- update(fit2, ~ . + WeekFactor:Treatment)
> print(summary(fit3), cor = FALSE)

Linear mixed model fit by REML ['lmerMod']
Formula:
log(1 - Proportion.of.Larvae) ~ (1 | HiveID) + WeekFactor + Treatment +
    WeekFactor:Treatment
   Data: Data

REML criterion at convergence: 0.6

Scaled residuals:
     Min       1Q   Median       3Q      Max
-1.32234 -0.44419  0.01893  0.50566  1.30689

Random effects:
 Groups   Name        Variance Std.Dev.
 HiveID   (Intercept) 0.141626 0.37633
 Residual             0.004949 0.07035
Number of obs: 32, groups:  HiveID, 16

Fixed effects:
                              Estimate Std. Error t value
(Intercept)                   -2.01225    0.19143 -10.512
WeekFactor6                    0.08708    0.04974   1.751
Treatment1 ug/l                1.32890    0.27072   4.909
Treatment10 ug/l               1.24031    0.27072   4.582
Treatment100 ug/l              1.93471    0.27072   7.147
WeekFactor6:Treatment1 ug/l    0.12036    0.07035   1.711
WeekFactor6:Treatment10 ug/l   0.10821    0.07035   1.538
WeekFactor6:Treatment100 ug/l -0.08708    0.07035  -1.238

>  # F-tests with approximated degrees of freedom according to Kenward-Roger:
>  #----------------------------------------------------------------------
> (KR0 <- KRmodcomp(largeModel = fit1, smallModel = fit0))

F-test with Kenward-Roger approximation; time: 0.13 sec
large : log(1 - Proportion.of.Larvae) ~ (1 | HiveID) + WeekFactor
small : log(1 - Proportion.of.Larvae) ~ (1 | HiveID)
        stat    ndf    ddf F.scaling  p.value
Ftest 15.387  1.000 15.000         1 0.001357 **
---
Signif. codes:  0 '***' 0.001 '**' 0.01 '*' 0.05 '.' 0.1 ' ' 1

> (KR1 <- KRmodcomp(largeModel = fit2, smallModel = fit1))

F-test with Kenward-Roger approximation; time: 0.09 sec
large : log(1 - Proportion.of.Larvae) ~ (1 | HiveID) + WeekFactor + Treatment
```

```
small : log(1 - Proportion.of.Larvae) ~ (1 | HiveID) + WeekFactor
       stat   ndf   ddf F.scaling   p.value
Ftest 18.04  3.00 12.00         1 9.629e-05 ***
---
Signif. codes:  0 '***' 0.001 '**' 0.01 '*' 0.05 '.' 0.1 ' ' 1

> (KR2 <- KRmodcomp(largeModel = fit3, smallModel = fit2))

F-test with Kenward-Roger approximation; time: 0.20 sec
large : log(1 - Proportion.of.Larvae) ~ (1 | HiveID) + WeekFactor + Treatment +
    WeekFactor:Treatment
small : log(1 - Proportion.of.Larvae) ~ (1 | HiveID) + WeekFactor + Treatment
         stat     ndf     ddf F.scaling p.value
Ftest  3.8761  3.0000 12.0000         1 0.03776 *
---
Signif. codes:  0 '***' 0.001 '**' 0.01 '*' 0.05 '.' 0.1 ' ' 1



>  # Parametric bootstrap:
>  #-----------------------------------------------------------------------
> cl <- makeCluster(rep("localhost", detectCores()))
> set.seed(201712)   # For reproducibility.
>  # Relevant: p-value in row "PBtest"
> (PB0 <- summary(PBmodcomp(largeM = fit1, smallM = fit0, nsim = nsim.PBmodcomp,
+                          cl = cl)))

Bootstrap test; time: 510.44 sec;samples: 5000; extremes: 6;
large : log(1 - Proportion.of.Larvae) ~ (1 | HiveID) + WeekFactor
small : log(1 - Proportion.of.Larvae) ~ (1 | HiveID)
           stat     df    ddf   p.value
LRT      11.312  1.000        0.0007699 ***
PBtest   11.312               0.0013997 **
Gamma    11.312               0.0013862 **
Bartlett 10.149  1.000        0.0014434 **
F        11.312  1.000 19.457 0.0031823 **
---
Signif. codes:  0 '***' 0.001 '**' 0.01 '*' 0.05 '.' 0.1 ' ' 1

> (PB1 <- summary(PBmodcomp(largeM = fit2, smallM = fit1, nsim = nsim.PBmodcomp,
+                          cl = cl)))

Bootstrap test; time: 528.91 sec;samples: 5000; extremes: 0;
large : log(1 - Proportion.of.Larvae) ~ (1 | HiveID) + WeekFactor + Treatment
small : log(1 - Proportion.of.Larvae) ~ (1 | HiveID) + WeekFactor
            stat     df    ddf   p.value
LRT      27.1063  3.0000        5.593e-06 ***
PBtest   27.1063                0.0002000 ***
Gamma    27.1063                0.0001102 ***
Bartlett 21.1325  3.0000        9.881e-05 ***
F         9.0354  3.0000 2.7022 0.0623470 .
---
Signif. codes:  0 '***' 0.001 '**' 0.01 '*' 0.05 '.' 0.1 ' ' 1

> (PB2 <- summary(PBmodcomp(largeM = fit3, smallM = fit2, nsim = nsim.PBmodcomp,
+                          cl = cl)))

Bootstrap test; time: 455.37 sec;samples: 5000; extremes: 177;
large : log(1 - Proportion.of.Larvae) ~ (1 | HiveID) + WeekFactor + Treatment +
    WeekFactor:Treatment
small : log(1 - Proportion.of.Larvae) ~ (1 | HiveID) + WeekFactor + Treatment
```

```
            stat      df    ddf p.value
LRT      11.0394  3.0000        0.01151 *
PBtest   11.0394                0.03559 *
Gamma    11.0394                0.03483 *
Bartlett  8.6800  3.0000        0.03386 *
F         3.6798  3.0000 2.7104 0.17119
---
Signif. codes:  0 '***' 0.001 '**' 0.01 '*' 0.05 '.' 0.1 ' ' 1

> stopCluster(cl)
```

Summary: There is a significant main effect of week (p-value = 0.001357 from Kenward-Rogers method, and p-value = 0.0014 based on parametric bootstrap), and there is a significant main effect of treatment (p-value = 9.629e-05 from Kenward-Rogers method, and p-value = 2e-04 based on parametric bootstrap).

Since there is a significant interaction effect between treatment and week (p-value = 0.03776 from Kenward-Rogers method, and p-value = 0.03559 based on parametric bootstrap), the main effect of each factor has to be interpreted as averaged across the levels of the other factor: The averages (across the four treatments) of log-proportions of dead larvae are significantly different between the two weeks, and the averaged log-proportions of dead larvae (across the two weeks) are significantly different between the four treatment groups.
For help in interpreting the above see the "more concrete" explanations in the summary on page 35.

Recall: the significant interaction effect between treatment and week means that changes (!) in (average) log-proportions of dead larvae along weeks are different between treatments.

Model diagnostics for the model in `fit3`:

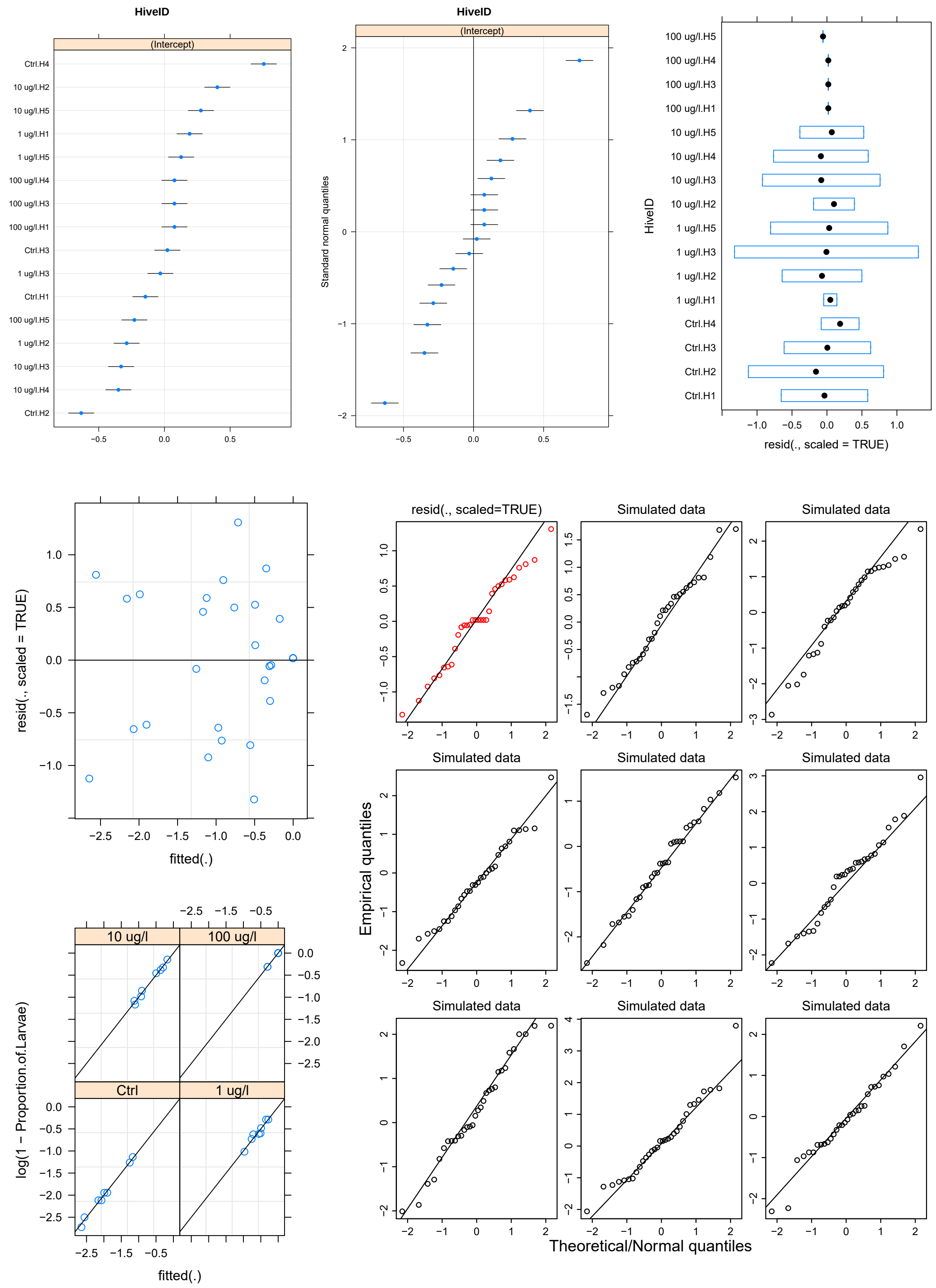

Figure 17: Diagnostic plots for the fitted linear mixed-effects model. Summarizing the findings (without explanation): the fitted model shows no serious indication against homoscedastic normality of the errors, so the inferential results are considered as reliable. (File names: *Cloth-Q3_Survival2ModelDiagPlotX.pdf* with $X = 1, \ldots, 6$)

2.3.5 Posthoc tests in survival phase 2 – comparisons with the Control

We proceed completely analogous to §2.3.3.

```
> fm <- fit3   # fm expects the 2-factorial mixed-effects ANOVA-object with interactions.
>  # Names of the 2 fixed-effects factors (Note: Order matters!):
> factornames <- list(Outer = "WeekFactor", Inner = "Treatment")
>  # Extract factor vector *data* which entered into fm via Data:
> InnerFactor <- Data[[factornames$Inner]]
> OuterFactor <- Data[[factornames$Outer]]
>  # 1. Dunnett's multiple comparisons for "inner" factor within "outer" factor:
>  #**********************************************************************
>   # Adapted from ch. 3 of multcomp-vignette "multcomp-examples". In particular the way
>   # of generating tmp for the model matrix X was modified to using levels() instead of
>   # unique(). (The latter would ignore a potentially non-alphanumerically sorted level
>   # order in the "inner factor" and could yield a wrong sign in contrast estimators.)
> tmp <- expand.grid(levels(InnerFactor), levels(OuterFactor))
> names(tmp) <- c(factornames$Inner, factornames$Outer)
> X <- model.matrix(formula(fm, fixed.only = TRUE)[-2], data = tmp)
> CM <- contrMat(table(InnerFactor), "Dunnett")   # Would also work with "Tukey".
> IM <- diag(nlevels(OuterFactor))
> dimnames(IM) <- list(levels(OuterFactor), levels(OuterFactor))
> Kron1 <- kronecker(IM, CM, make.dimnames = TRUE)

> fm.glht <- glht(fm, linfct = Kron1 %*% X, alternative = "greater")
> summary(fm.glht, test = adjusted(type = "Westfall"))

         Simultaneous Tests for General Linear Hypotheses

Fit: lmer(formula = log(1 - Proportion.of.Larvae) ~ (1 | HiveID) +
    WeekFactor + Treatment + WeekFactor:Treatment, data = Data,
    contrasts = list(WeekFactor = "contr.treatment", Treatment = "contr.treatment"))

Linear Hypotheses:
                       Estimate Std. Error z value Pr(>z)
5:1 ug/l - Ctrl <= 0     1.3289     0.2707   4.909 <1e-05 ***
5:10 ug/l - Ctrl <= 0    1.2403     0.2707   4.582 <1e-05 ***
5:100 ug/l - Ctrl <= 0   1.9347     0.2707   7.147 <1e-05 ***
6:1 ug/l - Ctrl <= 0     1.4493     0.2707   5.353 <1e-05 ***
6:10 ug/l - Ctrl <= 0    1.3485     0.2707   4.981 <1e-05 ***
6:100 ug/l - Ctrl <= 0   1.8476     0.2707   6.825 <1e-05 ***
---
Signif. codes:  0 '***' 0.001 '**' 0.01 '*' 0.05 '.' 0.1 ' ' 1
(Adjusted p values reported -- Westfall method)
```

Summary: In both weeks the log-proportions of dead larvae in hives treated with Clothianidin are significantly *higher* than in the Control group. (Compare bottom panel in fig. 15.)

Special consideration: Here, we select particular fixed effects coefficients of the fitted model for multiple comparisons with zero, namely coefficients number 6, 7, and 8 which means that we analyse only the interaction effects:

```
>  # 3. Selected fixed effects coefficients of fitted model are specified in
>  #    vector idx, e.g., for selected interaction effects:
>  #********************************************************************
> idx <- c(6, 7, 8)   # Can also be just a scalar, i.e., a single level index.
> fixcoef <- fixef(fm)
> K <- matrix(0, nrow = length(fixcoef), ncol = length(fixcoef),
+             dimnames = list(names(fixcoef), names(fixcoef)))
> diag(K)[idx] <- 1
> K <- K[rowSums(abs(K)) > 0, ]
```

```
> fm.glht3 <- glht(fm, linfct = K, alternative = "greater")
> # summary(fm.glht3)
> summary(fm.glht3, test = adjusted(type = "Westfall"))

         Simultaneous Tests for General Linear Hypotheses

Fit: lmer(formula = log(1 - Proportion.of.Larvae) ~ (1 | HiveID) +
    WeekFactor + Treatment + WeekFactor:Treatment, data = Data,
    contrasts = list(WeekFactor = "contr.treatment", Treatment = "contr.treatment"))

Linear Hypotheses:
                                   Estimate Std. Error z value Pr(>z)
WeekFactor6:Treatment1 ug/l <= 0    0.12036    0.07035   1.711  0.104
WeekFactor6:Treatment10 ug/l <= 0   0.10821    0.07035   1.538  0.107
WeekFactor6:Treatment100 ug/l <= 0 -0.08708    0.07035  -1.238  0.892
(Adjusted p values reported -- Westfall method)
```

Summary: The changes (!) from week 5 to week 6 in log-proportions of dead larvae are not significantly *higher* in any of the Clothianidin groups than in the Control group. (Compare bottom panel in fig. 15.)

Another special consideration: We carefully construct a combination of the one-sided tests from above:

```
> summary(glht(fm, linfct = rbind(Kron1 %*% X, K), alternative = "greater"),
+         test = adjusted(type = "Westfall"))

         Simultaneous Tests for General Linear Hypotheses

Fit: lmer(formula = log(1 - Proportion.of.Larvae) ~ (1 | HiveID) +
    WeekFactor + Treatment + WeekFactor:Treatment, data = Data,
    contrasts = list(WeekFactor = "contr.treatment", Treatment = "contr.treatment"))

Linear Hypotheses:
                                   Estimate Std. Error z value Pr(>z)
5:1 ug/l - Ctrl <= 0                1.32890    0.27072   4.909 <0.001 ***
5:10 ug/l - Ctrl <= 0               1.24031    0.27072   4.582 <0.001 ***
5:100 ug/l - Ctrl <= 0              1.93471    0.27072   7.147 <0.001 ***
6:1 ug/l - Ctrl <= 0                1.44926    0.27072   5.353 <0.001 ***
6:10 ug/l - Ctrl <= 0               1.34852    0.27072   4.981 <0.001 ***
6:100 ug/l - Ctrl <= 0              1.84763    0.27072   6.825 <0.001 ***
WeekFactor6:Treatment1 ug/l <= 0    0.12036    0.07035   1.711  0.104
WeekFactor6:Treatment10 ug/l <= 0   0.10821    0.07035   1.538  0.107
WeekFactor6:Treatment100 ug/l <= 0 -0.08708    0.07035  -1.238  0.892
---
Signif. codes:  0 '***' 0.001 '**' 0.01 '*' 0.05 '.' 0.1 ' ' 1
(Adjusted p values reported -- Westfall method)
```

Summary: If we combine the two test families from above into one family all statements from above hold still true (on the same significance level).

2.4 Question 4: Compensation rate

The main idea is to calculate and show the compensation effort for each treatment. The bees have to compensate for the higher larval mortality and therefore expend more energy.

2.4.1 Larvae-to-eggs ratio: Longitudinal EDA

To estimate/assess the development of the hives along time we calculate larvae-to-eggs ratios within each hive for each week. For this ratio to be sensible, the number of larvae in week w has to be compared with the number of eggs of the previous week $w-1$ (as the larvae of week w have been "produced" by the eggs of week $w-1$). The time courses of these "shifted" larvae-to-eggs ratios are then compared among treatments.

One point to consider is, that of all eggs seen in week $w-1$ approximately two thirds have already turned into capped brood at inspection in the subsequent week w because they were already one or two days old at inspection in week $w-1$. In turn, the actually observed number of larvae in week w can approximately be only one third of the number of eggs seen in the previous week.

However, we will not take this into consideration since it would complicate things unnecessarily since the deviation is very likely just a constant factor. We therefore take the raw weekly numbers of eggs and larvae as good estimates.

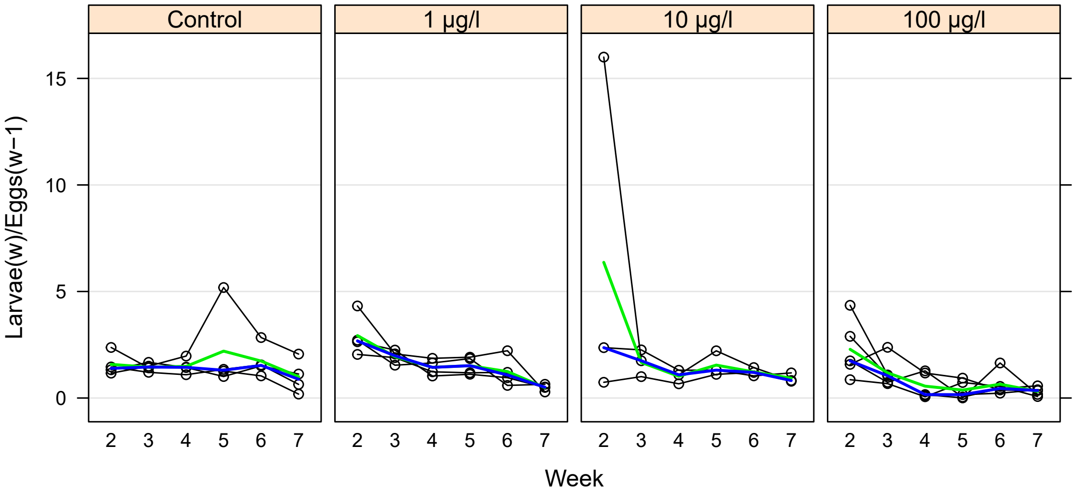

Figure 18: "Shifted" larvae-to-eggs ratio (open circles) in each hive along weeks by treatment, augmented by arithmetic means and medians across hives at each week. Values connected by a black polyline belong to the same hive; different polylines indicate different hives. The blue polylines connect the time specific median values, the green ones the respective means. (File name: *Cloth-Q4_L2E_along_Weeks_by_Treat.pdf*)

Table 1: Per treatment group: Mean and median larvae-to-eggs ratios per week

	**Week 2**		**Week 3**		**Week 4**		**Week 5**		**Week 6**		**Week 7**	
Treatment	Mean	Med.	Mean	Med.	Mean	Med.	Mean	Med.	Mean	Med.	Mean	Med.
Ctrl	1.58	1.39	1.45	1.45	1.48	1.44	2.20	1.30	1.73	1.53	1.00	0.88
1 ug/l	2.93	2.68	1.94	1.98	1.44	1.44	1.52	1.52	1.24	1.08	0.48	0.51
10 ug/l	6.37	2.36	1.67	1.75	1.02	1.09	1.54	1.31	1.22	1.20	0.93	0.82
100 ug/l	2.28	1.74	1.18	1.04	0.56	0.16	0.37	0.16	0.65	0.45	0.31	0.35

Table 2: Per treatment group and across weeks: Average and median of mean larvae-to-eggs ratios

	Ctrl	1 ug/l	10 ug/l	100 ug/l
Means of means	1.57	1.59	2.13	0.89
Medians of means	1.53	1.48	1.38	0.60

The following two figures display just the mean and median larvae-to-eggs ratio profiles, respectively, i.e., without the raw values.

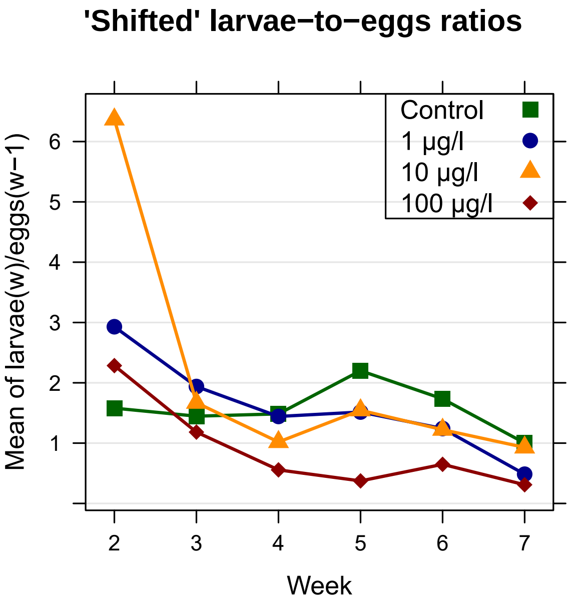

Figure 19: Means (across hives) of "shifted" larvae-to-eggs ratios along weeks by treatment. As eggs of week $w-1$ are the larvae of week w the numbers of larvae are shifted by one week. Note that the mean values in the control group stay quite steady at approx. 1.5 until week seven, when environmental conditions got worse. (File name: *Cloth-Q4_MeanL2E_along_Weeks_by_Treat.pdf*)

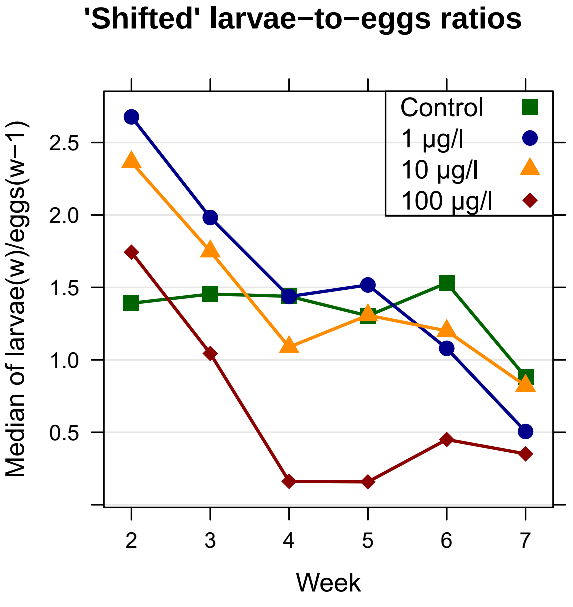

Figure 20: Medians (across hives) of "shifted" larvae-to-eggs ratios along weeks by treatment. As eggs of week $w-1$ are the larvae of week w the numbers of larvae are shifted by one week. Note that the median values in the control group stay quite steady very close to 1.5 until week seven, when environmental conditions got worse. (File name: *Cloth-Q4_MedianL2E_along_Weeks_by_Treat.pdf*)

2.4.2 Treatment effect on larvae-to-eggs ratio (WITHOUT the extreme outlier)

We again proceed as in §2.1.2, i.e., we follow, but also extend §10.6 in [6, Faraway (2016)]. For explanations regarding the testing methodology and for comments regarding the R-code see §2.1.2.

Note: We exclude the extreme outlier above 15 in treatment 10 µg/l from the following analysis!

```
> EL <- subset(EL0, subset = L2E < 15)
```

We analyse the larvae-to-eggs ratio by a linear mixed-effects model with a fixed effects linear time trend along weeks, with fixed treatment main effects, and fixed interaction effects between weeks and treatment. This is done for weeks centered at 2 – arbitrarily selected – so that the main effect of treatment represents the estimated larvae-to-eggs ratio at week 2. Hives are modelled as random shift effects, thus accounting for the within-hive correlation.

```
> levels(EL$Treatment) <- olE
> linfit1 <- lmer(L2E ~ I(Week - 2) + (1 | HiveID), data = EL)

> linfit2 <- update(linfit1, ~ . + Treatment,
+                   contrasts = list(Treatment = "contr.treatment"))
> linfit3 <- update(linfit2, ~ . + I(Week - 2):Treatment)
> print(summary(linfit3), cor = FALSE)

Linear mixed model fit by REML ['lmerMod']
Formula: L2E ~ I(Week - 2) + (1 | HiveID) + Treatment + I(Week - 2):Treatment
   Data: EL

REML criterion at convergence: 222.2

Scaled residuals:
    Min      1Q  Median      3Q     Max
-1.6475 -0.5277 -0.0943  0.2540  4.5530

Random effects:
 Groups   Name        Variance Std.Dev.
 HiveID   (Intercept) 0.08842  0.2973
 Residual             0.48522  0.6966
Number of obs: 95, groups:  HiveID, 16

Fixed effects:
                              Estimate Std. Error t value
(Intercept)                    1.66825    0.29265   5.700
I(Week - 2)                   -0.03743    0.08326  -0.450
Treatment1 ug/l                0.94119    0.41387   2.274
Treatment10 ug/l              -0.03515    0.46810  -0.075
Treatment100 ug/l              0.05734    0.39263   0.146
I(Week - 2):Treatment1 ug/l   -0.36948    0.11774  -3.138
I(Week - 2):Treatment10 ug/l  -0.08292    0.13269  -0.625
I(Week - 2):Treatment100 ug/l -0.29576    0.11170  -2.648

>  # F-tests with approximated degrees of freedom according to Kenward-Roger:
>  #----------------------------------------------------------------------
> # (KR1 <- KRmodcomp(largeModel = linfit2, smallModel = linfit1))
> (KR2 <- KRmodcomp(largeModel = linfit3, smallModel = linfit2))

F-test with Kenward-Roger approximation; time: 0.20 sec
large : L2E ~ I(Week - 2) + (1 | HiveID) + Treatment + I(Week - 2):Treatment
small : L2E ~ I(Week - 2) + (1 | HiveID) + Treatment
         stat     ndf     ddf F.scaling  p.value
Ftest  4.2802  3.0000 75.3889         1 0.007611 **
---
Signif. codes:  0 '***' 0.001 '**' 0.01 '*' 0.05 '.' 0.1 ' ' 1
```

```
> (KR3 <- KRmodcomp(largeModel = linfit3, smallM = update(linfit3, ~ . - Treatment)))

F-test with Kenward-Roger approximation; time: 0.17 sec
large : L2E ~ I(Week - 2) + (1 | HiveID) + Treatment + I(Week - 2):Treatment
small : L2E ~ I(Week - 2) + (1 | HiveID) + I(Week - 2):Treatment
         stat     ndf     ddf F.scaling p.value
Ftest  2.4851  3.0000 43.8312   0.99993 0.07313 .
---
Signif. codes:  0 '***' 0.001 '**' 0.01 '*' 0.05 '.' 0.1 ' ' 1
```

```
>  # Parametric bootstrap:
>  #------------------------------------------------------------------------
> cl <- makeCluster(rep("localhost", detectCores()))
> set.seed(201712)   # For reproducibility.
>  # Relevant: p-value in row "PBtest"
> # (PB1 <- summary(PBmodcomp(largeM = linfit2, smallM = linfit1, nsim = nsim.PBmodcomp,
> #                            cl = cl)))
> (PB2 <- summary(PBmodcomp(largeM = linfit3, smallM = linfit2, nsim = nsim.PBmodcomp,
+                           cl = cl)))

Bootstrap test; time: 420.33 sec;samples: 5000; extremes: 44;
large : L2E ~ I(Week - 2) + (1 | HiveID) + Treatment + I(Week - 2):Treatment
small : L2E ~ I(Week - 2) + (1 | HiveID) + Treatment
            stat      df    ddf  p.value
LRT      12.6663  3.0000        0.005417 **
PBtest   12.6663                0.008998 **
Gamma    12.6663                0.008019 **
Bartlett 11.7456  3.0000        0.008307 **
F         4.2221  3.0000 2.8948 0.138704
---
Signif. codes:  0 '***' 0.001 '**' 0.01 '*' 0.05 '.' 0.1 ' ' 1

> (PB3 <- summary(PBmodcomp(largeM = linfit3, smallM = update(linfit3, ~ . - Treatment),
+                           nsim = nsim.PBmodcomp, cl = cl)))

Bootstrap test; time: 419.61 sec;samples: 5000; extremes: 373;
large : L2E ~ I(Week - 2) + (1 | HiveID) + Treatment + I(Week - 2):Treatment
small : L2E ~ I(Week - 2) + (1 | HiveID) + I(Week - 2):Treatment
           stat     df    ddf p.value
LRT      7.9692 3.0000        0.04665 *
PBtest   7.9692               0.07479 .
Gamma    7.9692               0.07631 .
Bartlett 6.9319 3.0000        0.07410 .
F        2.6564 3.0000 2.8167 0.23086
---
Signif. codes:  0 '***' 0.001 '**' 0.01 '*' 0.05 '.' 0.1 ' ' 1

> stopCluster(cl)
```

Summary: Allowing a linear time trend along weeks with interaction between time and treatment there is a significant interaction between time and treatment (p-value = 0.007611 from the Kenward-Rogers method, and p-value = 0.008998 based on parametric bootstrap).
This means in particular, that the time trends of the larvae-to-eggs ratio are significantly different between the four treatment groups.

However, there is no significant treatment main effect in the model with interaction (p-value = 0.07313 from the Kenward-Rogers method, and p-value = 0.07479 based on parametric bootstrap). This means, the estimated average larvae-to-eggs ratio at week 2 (!) are not significantly different between the four treatment groups.

Model diagnostics for the model in `linfit3`:

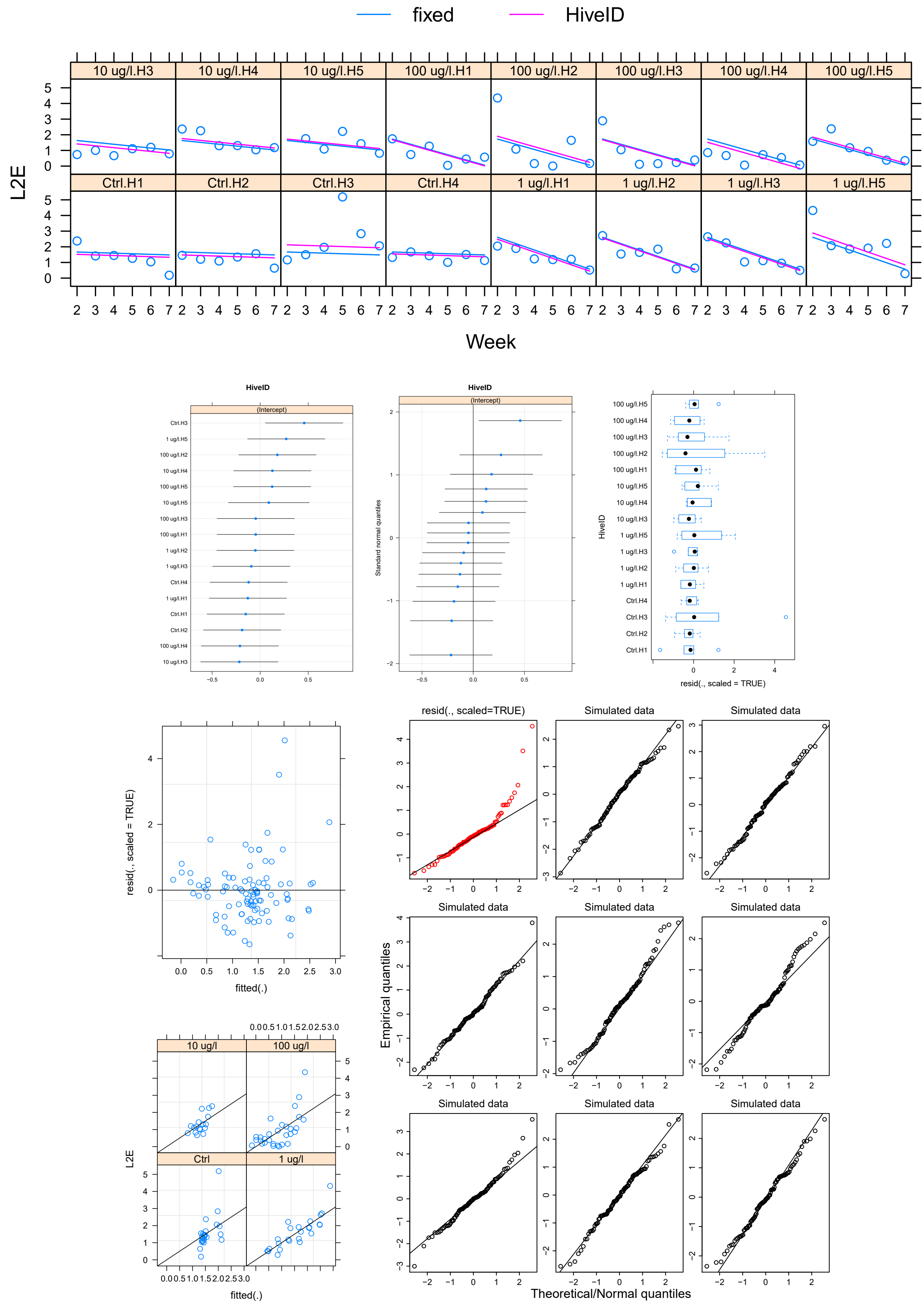

Figure 21: Diagnostic plots for the fitted linear mixed-effects model with *linear* time trends. Summarizing the findings (without explanation): the fitted model does show some indication against homoscedastic normality of the errors, so the inferential results should be interpreted with care. (File names: *Cloth-Q4_LinFit_L2EModelAugPred.pdf* and *Cloth-Q4_LinFit_L2EModelDiagX.pdf* with $X = 1, \ldots, 6$)

2.4.3 Posthoc tests for time trend of larvae-to-eggs ratio (WITHOUT the extreme outlier) – comparisons with the Control

Here, we compare each Clothianidin treatment group to the control group with respect to the time trends in larvae-to-eggs ratios.

```
> fx <- fixef(linfit3)

> K <- diag(length(fx))[-(1:5),]
> rownames(K) <- names(fx)[-(1:5)]
> CompWCntrl <- glht(linfit3, linfct = K) # summary(CompWCntrl)
> summary(CompWCntrl, test = adjusted(type = "Westfall"))

         Simultaneous Tests for General Linear Hypotheses

Fit: lmer(formula = L2E ~ I(Week - 2) + (1 | HiveID) + Treatment +
    I(Week - 2):Treatment, data = EL, contrasts = list(Treatment = "contr.treatment"))

Linear Hypotheses:
                                   Estimate Std. Error z value Pr(>|z|)
I(Week - 2):Treatment1 ug/l == 0   -0.36948    0.11774  -3.138  0.00497 **
I(Week - 2):Treatment10 ug/l == 0  -0.08292    0.13269  -0.625  0.53202
I(Week - 2):Treatment100 ug/l == 0 -0.29576    0.11170  -2.648  0.01557 *
---
Signif. codes:  0 '***' 0.001 '**' 0.01 '*' 0.05 '.' 0.1 ' ' 1
(Adjusted p values reported -- Westfall method)
```

Summary: The linear time trends of the larvae-to-eggs ratio in the Clothianidin treatments with 1 µg/l and 100 µg/l are significantly different from the one in the control group.

2.4.4 Treatment effect on larvae-to-eggs ratio (WITH the extreme outlier)

Almost identical to §2.4.2.

Note: Here, we INclude the extreme outlier above 15 in treatment group 10 µg/l in the following analysis!

```
> EL <- EL0

> levels(EL$Treatment) <- olE
> linfit1 <- lmer(L2E ~ I(Week - 2) + (1 | HiveID), data = EL)
> linfit2 <- update(linfit1, ~ . + Treatment,
+                   contrasts = list(Treatment = "contr.treatment"))
> linfit3 <- update(linfit2, ~ . + I(Week - 2):Treatment)
> print(summary(linfit3), cor = FALSE)

Linear mixed model fit by REML ['lmerMod']
Formula: L2E ~ I(Week - 2) + (1 | HiveID) + Treatment + I(Week - 2):Treatment
   Data: EL

REML criterion at convergence: 359.1

Scaled residuals:
    Min      1Q  Median      3Q     Max
-1.9802 -0.3154 -0.0671  0.0992  7.3967

Random effects:
 Groups   Name        Variance Std.Dev.
 HiveID   (Intercept) 0.1571   0.3963
 Residual             2.3641   1.5376
Number of obs: 96, groups:  HiveID, 16

Fixed effects:
                              Estimate Std. Error t value
(Intercept)                    1.66825    0.59064   2.824
I(Week - 2)                   -0.03743    0.18378  -0.204
Treatment1 ug/l                0.94119    0.83529   1.127
Treatment10 ug/l               2.45767    0.90222   2.724
Treatment100 ug/l              0.05734    0.79242   0.072
I(Week - 2):Treatment1 ug/l   -0.36948    0.25990  -1.422
I(Week - 2):Treatment10 ug/l  -0.76278    0.28072  -2.717
I(Week - 2):Treatment100 ug/l -0.29576    0.24656  -1.200

> linfit3.L2E <- linfit3

>  # F-tests with approximated degrees of freedom according to Kenward-Roger:
>  #-----------------------------------------------------------------------
> # (KR1 <- KRmodcomp(largeModel = linfit2, smallModel = linfit1))
> (KR2 <- KRmodcomp(largeModel = linfit3, smallModel = linfit2))

F-test with Kenward-Roger approximation; time: 0.14 sec
large : L2E ~ I(Week - 2) + (1 | HiveID) + Treatment + I(Week - 2):Treatment
small : L2E ~ I(Week - 2) + (1 | HiveID) + Treatment
         stat     ndf     ddf F.scaling p.value
Ftest  2.4909  3.0000 76.0000         1 0.06655 .
---
Signif. codes:  0 '***' 0.001 '**' 0.01 '*' 0.05 '.' 0.1 ' ' 1

> (KR3 <- KRmodcomp(largeModel = linfit3, smallM = update(linfit3, ~ . - Treatment)))

F-test with Kenward-Roger approximation; time: 0.14 sec
large : L2E ~ I(Week - 2) + (1 | HiveID) + Treatment + I(Week - 2):Treatment
small : L2E ~ I(Week - 2) + (1 | HiveID) + I(Week - 2):Treatment
```

```
         stat     ndf     ddf F.scaling p.value
Ftest  3.2303  3.0000 56.1350         1 0.02906 *
---
Signif. codes:  0 '***' 0.001 '**' 0.01 '*' 0.05 '.' 0.1 ' ' 1
```

```
>  # Parametric bootstrap:
>  #------------------------------------------------------------------------
> cl <- makeCluster(rep("localhost", detectCores()))
> set.seed(201712)   # For reproducibility.
>  # Relevant: p-value in row "PBtest"
> # (PB1 <- summary(PBmodcomp(largeM = linfit2, smallM = linfit1, nsim = nsim.PBmodcomp,
> #                             cl = cl)))
> (PB2 <- summary(PBmodcomp(largeM = linfit3, smallM = linfit2, nsim = nsim.PBmodcomp,
+                           cl = cl)))

Bootstrap test; time: 418.36 sec;samples: 5000; extremes: 359;
large : L2E ~ I(Week - 2) + (1 | HiveID) + Treatment + I(Week - 2):Treatment
small : L2E ~ I(Week - 2) + (1 | HiveID) + Treatment
           stat     df    ddf p.value
LRT      7.6240 3.0000        0.05446 .
PBtest   7.6240               0.07199 .
Gamma    7.6240               0.07186 .
Bartlett 7.0529 3.0000        0.07023 .
F        2.5413 3.0000 2.8917 0.23715
---
Signif. codes:  0 '***' 0.001 '**' 0.01 '*' 0.05 '.' 0.1 ' ' 1

> (PB3 <- summary(PBmodcomp(largeM = linfit3, smallM = update(linfit3, ~ . - Treatment),
+                           nsim = nsim.PBmodcomp, cl = cl)))

Bootstrap test; time: 472.53 sec;samples: 5000; extremes: 130;
large : L2E ~ I(Week - 2) + (1 | HiveID) + Treatment + I(Week - 2):Treatment
small : L2E ~ I(Week - 2) + (1 | HiveID) + I(Week - 2):Treatment
            stat      df    ddf p.value
LRT      10.2567  3.0000        0.01651 *
PBtest   10.2567                0.02619 *
Gamma    10.2567                0.02755 *
Bartlett  9.0759  3.0000        0.02830 *
F         3.4189  3.0000 2.8367 0.17785
---
Signif. codes:  0 '***' 0.001 '**' 0.01 '*' 0.05 '.' 0.1 ' ' 1

> stopCluster(cl)
```

Summary: Allowing a linear time trend along weeks with interaction between time and treatment there IS NO significant interaction between time and treatment (p-value = 0.06655 from the Kenward-Rogers method, and p-value = 0.07199 based on parametric bootstrap).
This means in particular, that the time trends of the larvae-to-eggs ratio ARE NOT significantly different between the four treatment groups.

However, there IS A significant treatment main effect in the model with interaction (p-value = 0.02906 from the Kenward-Rogers method, and p-value = 0.02619 based on parametric bootstrap). This means, the estimated average larvae-to-eggs ratio at week 2 (!) ARE significantly different between the four treatment groups.

Model diagnostics for the model in `linfit3`:

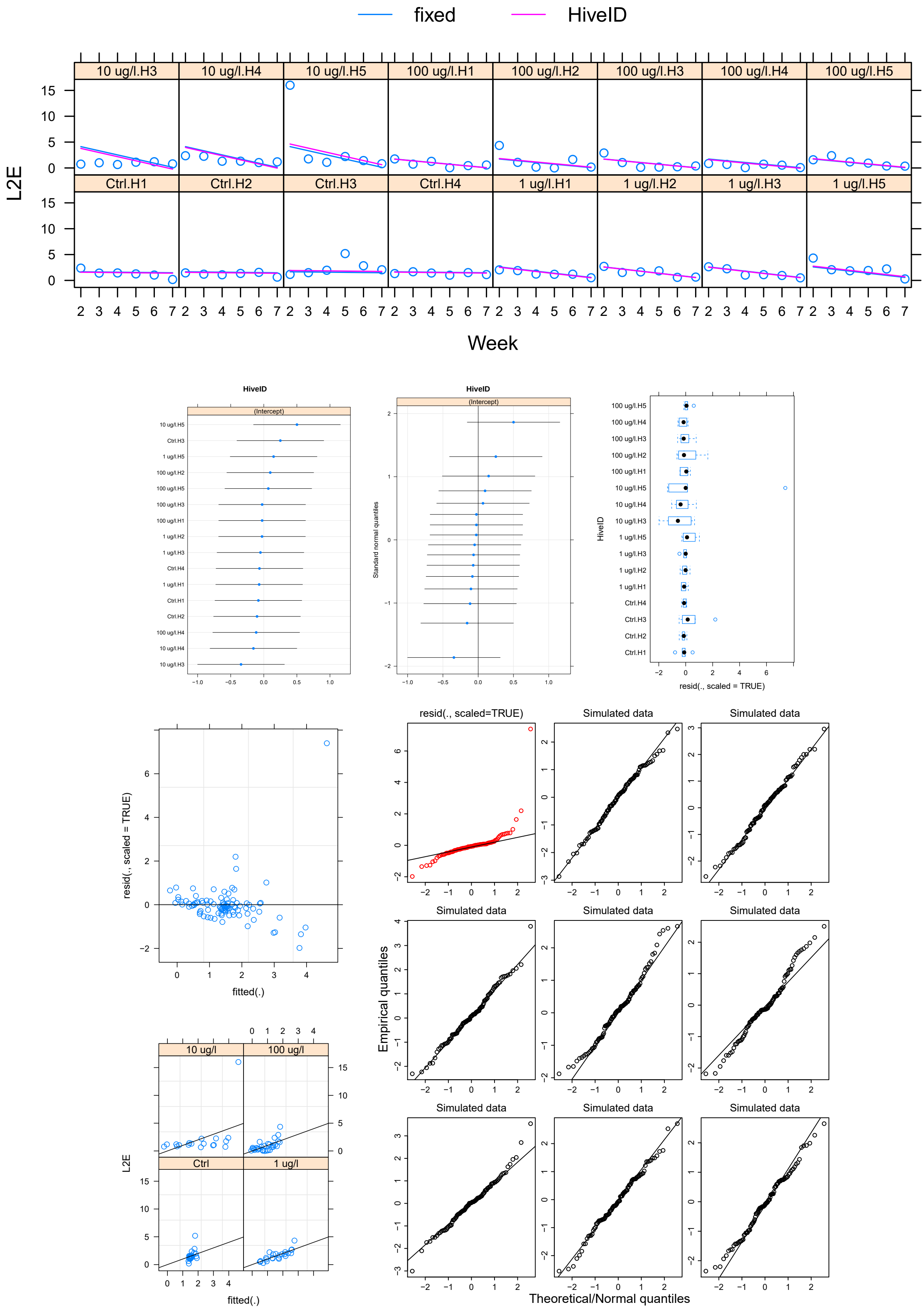

Figure 22: Diagnostic plots for the fitted linear mixed-effects model with *linear* time trends. Summarizing the findings (without explanation): the fitted model does show some indication against homoscedastic normality of the errors, so the inferential results should be interpreted with care. (File names: *Cloth-Q4_LinFit_L2EModelAugPred.pdf* and *Cloth-Q4_LinFit_L2EModelDiagX_.pdf* with $X = 1, \ldots, 6$)

2.4.5 Posthoc tests for time trend of larvae-to-eggs ratio (WITH the extreme outlier) – comparisons with the Control

Here, we compare each Clothianidin treatment group to the control group with respect to the time trends in larvae-to-eggs ratios.

```
> fx <- fixef(linfit3)

> K <- diag(length(fx))[-(1:5),]
> rownames(K) <- names(fx)[-(1:5)]
> CompWCntrl <- glht(linfit3, linfct = K) # summary(CompWCntrl)
> summary(CompWCntrl, test = adjusted(type = "Westfall"))

         Simultaneous Tests for General Linear Hypotheses

Fit: lmer(formula = L2E ~ I(Week - 2) + (1 | HiveID) + Treatment +
    I(Week - 2):Treatment, data = EL, contrasts = list(Treatment = "contr.treatment"))

Linear Hypotheses:
                                   Estimate Std. Error z value Pr(>|z|)
I(Week - 2):Treatment1 ug/l == 0    -0.3695     0.2599  -1.422    0.262
I(Week - 2):Treatment10 ug/l == 0   -0.7628     0.2807  -2.717    0.018 *
I(Week - 2):Treatment100 ug/l == 0  -0.2958     0.2466  -1.200    0.262
---
Signif. codes:  0 '***' 0.001 '**' 0.01 '*' 0.05 '.' 0.1 ' ' 1
(Adjusted p values reported -- Westfall method)
```

Summary: The linear time trend of the larvae-to-eggs ratio in the Clothianidin treatments with 10 µg/l IS significantly different from the one in the control group.

2.4.6 Capped-brood-to-larvae ratio

Here the same is done for the capped-brood-to-larvae ratio as was done in §2.4.1 for the larvae-to-eggs ratio.

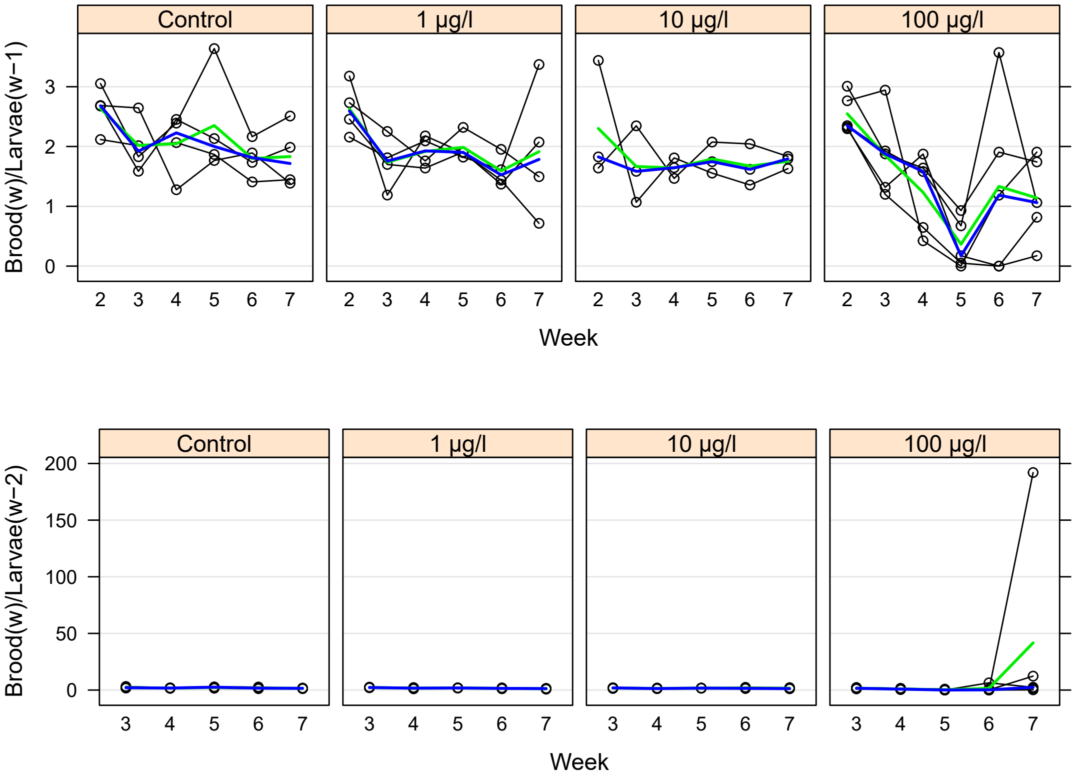

Figure 23: Differently "shifted" capped-brood-to-larvae ratio (open circles) in each hive along weeks by treatment, augmented by arithmetic means and medians across hives at each week. Values connected by a black polyline belong to the same hive; different polylines indicate different hives. The blue polylines connect the time specific median values, the green ones the respective means. (File names: *Cloth-Q4_B2L_along_Weeks_by_Treat.pdf* and *Cloth-Q4_B2L_along_Weeks_by_Treat_2.pdf*)

Table 3: Per treatment group: Mean and median capped-brood-to-larvae ratios per week (shifted by lag 1)

	**Week 2**		**Week 3**		**Week 4**		**Week 5**		**Week 6**		**Week 7**	
Treatment	Mean	Med.	Mean	Med.	Mean	Med.	Mean	Med.	Mean	Med.	Mean	Med.
Ctrl	2.63	2.68	2.02	1.92	2.05	2.23	2.35	2.00	1.80	1.81	1.83	1.72
1 ug/l	2.63	2.59	1.74	1.76	1.92	1.93	1.99	1.90	1.59	1.52	1.91	1.78
10 ug/l	2.30	1.83	1.67	1.58	1.64	1.65	1.79	1.75	1.67	1.62	1.75	1.79
100 ug/l	2.55	2.35	1.85	1.88	1.23	1.58	0.36	0.17	1.33	1.19	1.14	1.06

Table 4: Per treatment group and across weeks: Average and median of mean capped-brood-to-larvae ratios (shifted by lag 1)

	Ctrl	1 ug/l	10 ug/l	100 ug/l
Means of means	2.11	1.96	1.80	1.41
Medians of means	2.03	1.92	1.71	1.28

The following two figures display just the mean and median capped-brood-to-larvae ratio profiles (shifted by lag 1), respectively, i.e., without the raw values.

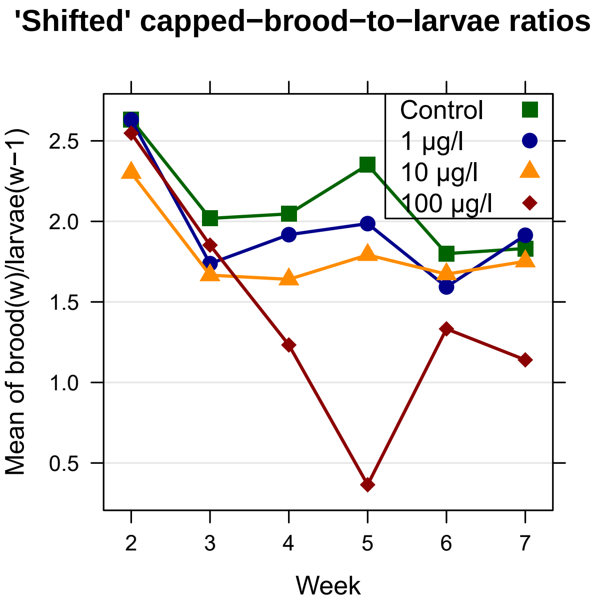

Figure 24: Means (across hives) of "shifted" capped-brood-to-larvae ratios along weeks by treatment. As eggs of week $w-1$ are the larvae of week w the numbers of larvae are shifted by one week. Note that the mean values in the control group are quite consistently the largest values and that all "low-dose"-groups (incl. Control) stay above 1.5. (File name: *Cloth-Q4_MeanB2L_along_Weeks_by_Treat.pdf*)

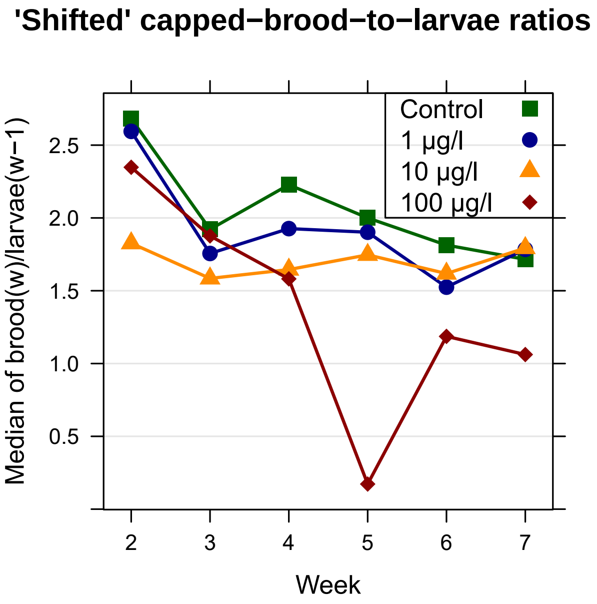

Figure 25: Medians (across hives) of "shifted" capped-brood-to-larvae ratios along weeks by treatment. As eggs of week $w-1$ are the larvae of week w the numbers of larvae are shifted by one week. Note that the median values in the control group are quite consistently the largest values and that all "low-dose"-groups (incl. Control) stay above 1.5. (File name: *Cloth-Q4_MedianB2L_along_Weeks_by_Treat.pdf*)

2.4.7 Treatment effect on capped-brood-to-larvae ratio

We proceed as before in §2.4.2; for details see there.

```
> levels(BL1$Treatment) <- olE
> linfit1 <- lmer(B2L ~ I(Week - 2) + (1 | HiveID), data = BL1)

> linfit2 <- update(linfit1, ~ . + Treatment,
+                   contrasts = list(Treatment = "contr.treatment"))
> linfit3 <- update(linfit2, ~ . + I(Week - 2):Treatment)
> print(summary(linfit3), cor = FALSE)

Linear mixed model fit by REML ['lmerMod']
Formula: B2L ~ I(Week - 2) + (1 | HiveID) + Treatment + I(Week - 2):Treatment
   Data: BL1

REML criterion at convergence: 208.4

Scaled residuals:
    Min      1Q  Median      3Q     Max 
-2.0574 -0.5292 -0.0531  0.4513  3.6949 

Random effects:
 Groups   Name        Variance Std.Dev.
 HiveID   (Intercept) 0.04714  0.2171  
 Residual             0.41624  0.6452  
Number of obs: 96, groups:  HiveID, 16

Fixed effects:
                              Estimate Std. Error t value
(Intercept)                    2.42479    0.25747   9.418
I(Week - 2)                   -0.12455    0.07711  -1.615
Treatment1 ug/l               -0.17947    0.36412  -0.493
Treatment10 ug/l              -0.43654    0.39330  -1.110
Treatment100 ug/l             -0.33678    0.34544  -0.975
I(Week - 2):Treatment1 ug/l    0.01170    0.10905   0.107
I(Week - 2):Treatment10 ug/l   0.05090    0.11779   0.432
I(Week - 2):Treatment100 ug/l -0.14593    0.10346  -1.410

>  # F-tests with approximated degrees of freedom according to Kenward-Roger:
>  #----------------------------------------------------------------------
> # (KR1 <- KRmodcomp(largeModel = linfit2, smallModel = linfit1))
> (KR2 <- KRmodcomp(largeModel = linfit3, smallModel = linfit2))

F-test with Kenward-Roger approximation; time: 0.12 sec
large : B2L ~ I(Week - 2) + (1 | HiveID) + Treatment + I(Week - 2):Treatment
small : B2L ~ I(Week - 2) + (1 | HiveID) + Treatment
         stat     ndf     ddf F.scaling p.value
Ftest  1.3626  3.0000 76.0000         1  0.2607

> (KR3 <- KRmodcomp(largeModel = linfit3, smallM = update(linfit3, ~ . - Treatment)))

F-test with Kenward-Roger approximation; time: 0.14 sec
large : B2L ~ I(Week - 2) + (1 | HiveID) + Treatment + I(Week - 2):Treatment
small : B2L ~ I(Week - 2) + (1 | HiveID) + I(Week - 2):Treatment
         stat     ndf     ddf F.scaling p.value
Ftest  0.5113  3.0000 49.4476         1  0.6764


>  # Parametric bootstrap:
>  #----------------------------------------------------------------------
```

```
> cl <- makeCluster(rep("localhost", detectCores()))
> set.seed(201712)   # For reproducibility.
>  # Relevant: p-value in row "PBtest"
> # (PB1 <- summary(PBmodcomp(largeM = linfit2, smallM = linfit1, nsim = nsim.PBmodcomp,
> #                            cl = cl)))
> (PB2 <- summary(PBmodcomp(largeM = linfit3, smallM = linfit2, nsim = nsim.PBmodcomp,
+                           cl = cl)))

Bootstrap test; time: 498.60 sec;samples: 5000; extremes: 1279;
large : B2L ~ I(Week - 2) + (1 | HiveID) + Treatment + I(Week - 2):Treatment
small : B2L ~ I(Week - 2) + (1 | HiveID) + Treatment
           stat     df    ddf p.value
LRT      4.3121 3.0000         0.2297
PBtest   4.3121                0.2559
Gamma    4.3121                0.2559
Bartlett 4.0565 3.0000         0.2554
F        1.4374 3.0000 2.9137  0.3896

> (PB3 <- summary(PBmodcomp(largeM = linfit3, smallM = update(linfit3, ~ . - Treatment),
+                           nsim = nsim.PBmodcomp, cl = cl)))

Bootstrap test; time: 510.00 sec;samples: 5000; extremes: 3548;
large : B2L ~ I(Week - 2) + (1 | HiveID) + Treatment + I(Week - 2):Treatment
small : B2L ~ I(Week - 2) + (1 | HiveID) + I(Week - 2):Treatment
           stat      df   ddf p.value
LRT      1.5730 3.00000        0.6655
PBtest   1.5730                0.7097
Gamma    1.5730                0.7116
Bartlett 1.3876 3.00000        0.7084
F        0.5243 3.00000 2.833  0.6969

> stopCluster(cl)
```

Summary: Allowing a linear time trend along weeks with interaction between time and treatment there is no significant interaction between time and treatment (p-value = 0.2607 from the Kenward-Rogers method, and p-value = 0.2559 based on parametric bootstrap).
This means in particular, that the time trends of the capped-brood-to-larvae ratio are not significantly different between the four treatment groups.

And there is no significant treatment main effect in the model with interaction (p-value = 0.6764 from the Kenward-Rogers method, and p-value = 0.7097 based on parametric bootstrap). This means, the estimated average capped-brood-to-larvae ratios at week 2 (!) are not significantly different between the four treatment groups.

Model diagnostics for the model in `linfit3`:

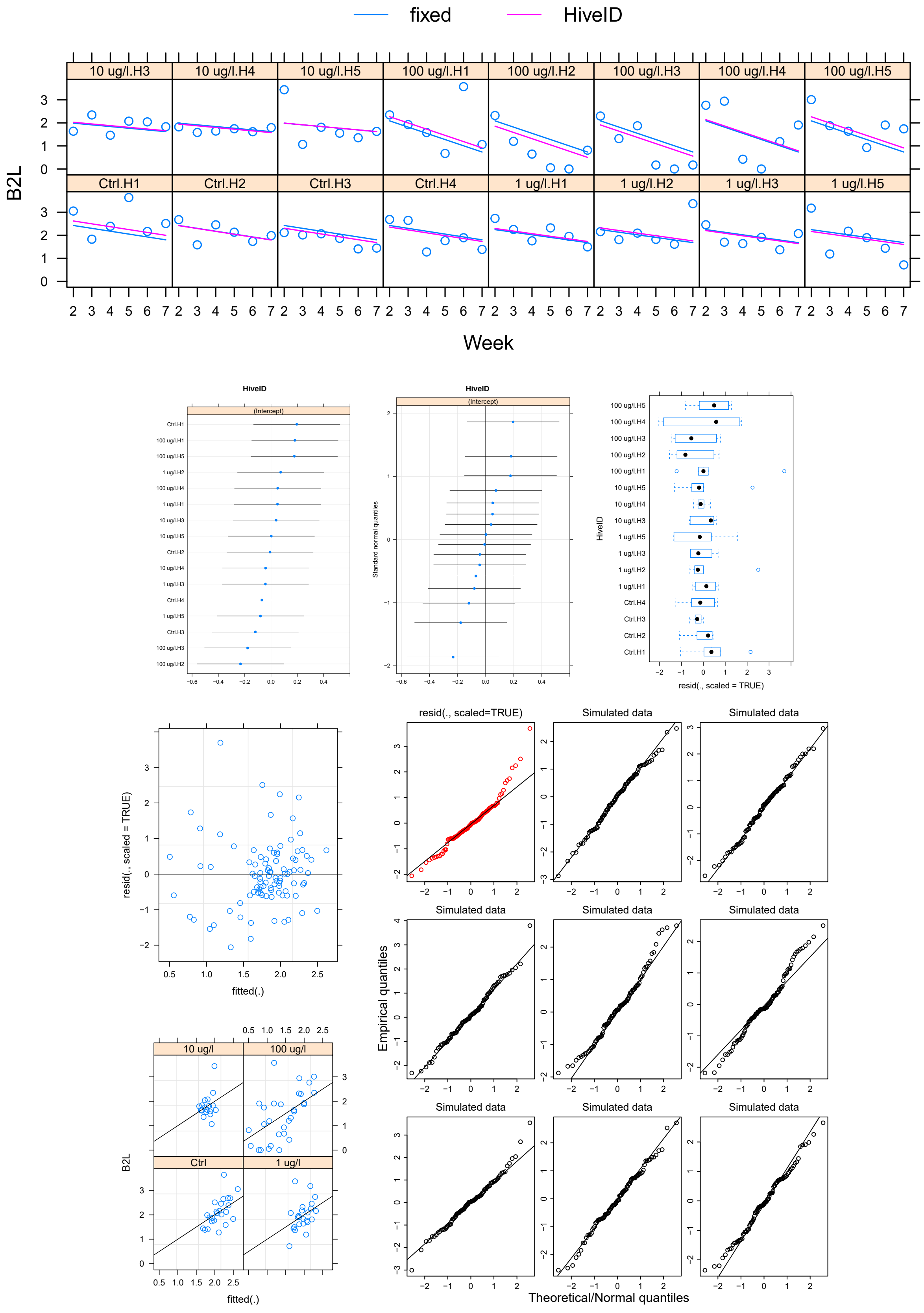

Figure 26: Diagnostic plots for the fitted linear mixed-effects model with *linear* time trends. Summarizing the findings (without explanation): the fitted model appears to fit and does not show a too serious indication against homoscedastic normality of the errors, so the inferential results are considered reliable. (File names: *Cloth-Q4_LinFit_B2LModelAugPred.pdf* and *Cloth-Q4_LinFit_B2LModelDiagX.pdf* with $X = 1, \ldots, 6$)

2.4.8 Posthoc tests for time trend of capped-brood-to-larvae ratio – comparisons with the Control

Here, we compare each Clothianidin treatment group to the control group with respect to the time trends in capped-brood-to-larvae ratios.

```
> fx <- fixef(linfit3)

> K <- diag(length(fx))[-(1:5),]
> rownames(K) <- names(fx)[-(1:5)]
> CompWCntrl <- glht(linfit3, linfct = K) # summary(CompWCntrl)
> summary(CompWCntrl, test = adjusted(type = "Westfall"))

         Simultaneous Tests for General Linear Hypotheses

Fit: lmer(formula = B2L ~ I(Week - 2) + (1 | HiveID) + Treatment +
    I(Week - 2):Treatment, data = BL1, contrasts = list(Treatment = "contr.treatment"))

Linear Hypotheses:
                                   Estimate Std. Error z value Pr(>|z|)
I(Week - 2):Treatment1 ug/l == 0     0.0117     0.1090   0.107    0.915
I(Week - 2):Treatment10 ug/l == 0    0.0509     0.1178   0.432    0.876
I(Week - 2):Treatment100 ug/l == 0  -0.1459     0.1035  -1.410    0.356
(Adjusted p values reported -- Westfall method)
```

Summary: The Clothianidin treatments do not differ significantly from the control group in their linear time trends of the capped-brood-to-larvae ratio.

3 Software & References

All graphics and statistical calculations or analyses have been created or made with the "open-source" software R version 3.6.3 (2020-02-29) [1], a programming language and environment for statistical computing and graphics, including the packages:

- **lattice** (for graphics), see *especially* [2] for reference,
- **lme4** (for linear mixed-effects models) with [3] as reference, and for whose underlying mathematical-statistical concepts [4] is a valuable source, and whose most recent details can be found at `http://lme4.r-forge.r-project.org`,
- **pbkrtest** (for testing fixed effects in mixed-effects models using *p*arametric *b*ootstrap, i.e., a simulation-based method, and the *K*enward-*R*oger-method of adjusted degrees of freedom) [5] with examples (and, e.g., [6] for further examples of applications),
- **parallel** (to support computations by parallel computing) [1],
- **multcomp** (for multiple pairwise comparisons) with [7] as main reference and [8] as even more extensive source, and
- **RColorBrewer** (for colors of particular figures) [10].

This report was generated with LaTeX, where [11] as well as [12] are relevant as references for the inclusion of R-code and its results into this report utilizing the R-function Sweave, and where [13] (for the R-package **Hmisc**) and [14] are references for creating the (few) LaTeX-tables from within R using also Sweave. This all happened in the "integrated development environment" (IDE) RStudio, Version 1.2.1335 [15]. The complete Rnw-files (R- and LaTeX-Code) of this report can be requested by email from the authors.

References

[1] R Core Team (2020). R: A language and environment for statistical computing. R Foundation for Statistical Computing, Vienna, Austria. `https://www.R-project.org/`.

[2] Sarkar, Deepayan (2008) Lattice: Multivariate Data Visualization with R. Springer, New York. ISBN 978-0-387-75968-5. `http://lmdvr.r-forge.r-project.org`.

[3] Douglas Bates, Martin Maechler, Ben Bolker, Steve Walker (2015). Fitting Linear Mixed-Effects Models Using lme4. Journal of Statistical Software, 67(1), 1-48. `http://dx.doi.org/10.18637/jss.v067.i01.`.

[4] Pinheiro, J. C., Bates, D. M. (2001). Mixed-effects models in S and S-PLUS. Corr. 2nd ed. New York, NY, Springer-Verlag.

[5] Ulrich Halekoh, Soeren Hoejsgaard (2014). A Kenward-Roger Approximation and Parametric Bootstrap Methods for Tests in Linear Mixed Models - The R Package pbkrtest. Journal of Statistical Software, 59(9), 1-30. `https://www.jstatsoft.org/v59/i09/.`.

[6] Faraway, J. J. (2016). Extending the linear model with R. 2nd ed., Chapman & Hall/CRC, Boca Raton/Florida.

[7] Torsten Hothorn, Frank Bretz and Peter Westfall (2008). Simultaneous Inference in General Parametric Models. Biometrical Journal 50(3), 346–363. `http://cran.r-project.org/web/packages/multcomp`.

[8] Bretz, F., Hothorn, T., Westfall, P. (2010). Multiple Comparisons Using R. Chapman & Hall/CRC, Boca Raton/Florida.

[9] Hsu, J. C. (1996): Multiple Comparisons. Theory and methods. Chapman & Hall/CRC, London.

[10] Erich Neuwirth (2014). RColorBrewer: ColorBrewer Palettes. R package version 1.1-2. `https://CRAN.R-project.org/package=RColorBrewer`.

[11] Leisch, F. (2002). Sweave User Manual. `http://www.statistik.lmu.de/~leisch/Sweave/`.

(There exists a much newer version of this manual as *vignette*, which can be "unearthed" by the R-command `vignette("Sweave")`, or at `https://stat.ethz.ch/R-manual/R-devel/library/utils/doc/Sweave.pdf`.)

[12] Leisch, F. (2002). Dynamic generation of statistical reports using literate data analysis. In W. Härdle and B. Rönz, editors, Compstat 2002 - Proceedings in Computational Statistics, pages 575-580. Physika Verlag, Heidelberg, Germany. ISBN 3-7908-1517-9.

[13] Frank E Harrell Jr, with contributions from Charles Dupont and many others. (2020). Hmisc: Harrell Miscellaneous. R package version 4.4-1. `https://CRAN.R-project.org/package=Hmisc`

[14] Whiting, D. (2005). Some examples of conditional typesetting using the latex() function. `http://biostat.mc.vanderbilt.edu/twiki/pub/Main/StatReport/latexFineControl.pdf`

[15] RStudio Team (2019). RStudio: Integrated Development Environment for R. RStudio, Inc., Boston, MA. URL `http://www.rstudio.com/`
